# Validated strategies for screening for eating disorders in primary health care: A scoping review with a focus on adolescents and adults

**DOI:** 10.1371/journal.pone.0347184

**Published:** 2026-08-03

**Authors:** Lisane da Silva Oliveira, Karine Lima Curvello-Silva, Priscila Ribas de Farias Costa, Isabelle de Jesus Peneluc Menezes, Patrícia Fortes Cavalcanti de Macêdo, Aline Monteiro dos Santos Ruas, Renata Alves Monteiro, Carlos Rodrigo Nascimento de Lira, Evelyn Caldas Abreu, Carla de Magalhães Cunha, Mônica Leila Portela de Santana

**Affiliations:** 1 Graduate Program in Food, Nutrition and Health, School of Nutrition, Federal University of Bahia, Salvador, Bahia, Brazil; 2 Department of Nutrition Science, School of Nutrition, Federal University of Bahia, Salvador, Bahia, Brazil; 3 Center for the Integration of Data and Knowledge for Health (CIDACS/Fiocruz – NIH – Harvard), Salvador, Brazil; 4 School of Nutrition, Federal University of Bahia, Salvador, Bahia, Brazil; 5 Municipal Health Department of Salvador, Salvador, Brazil; 6 Department of Nutrition, Faculty of Health Sciences, University of Brasilia, Brasilia, Brazil; Necmettin Erbakan Üniversitesi: Necmettin Erbakan Universitesi, TÜRKIYE

## Abstract

Eating disorders are serious, complex, and potentially chronic psychiatric conditions that affect millions of people worldwide. Primary health care plays a strategic role in the early identification and referral, helping to reduce complications, mortality, and health system burden. This scoping review aimed to map the available evidence on validated strategies for screening for eating disorders in adolescents and adults in primary care. The study followed the Preferred Reporting Items for Systematic reviews and Meta-Analyses extension for Scoping Reviews, with a protocol registered in the Open Science Framework (doi: 10.17605/OSF.IO/498FU). Searches were conducted in MEDLINE, Embase, Latin American and Caribbean Health Sciences Literature, Web of Science, PsycINFO, Cumulative Index to Nursing and Allied Health Literature and gray literature in October 2024. Data selection and extraction were performed by peer reviewers, and findings were synthesized narratively, with tables and figures. Of 2977 documents and 159 full texts assessed, 77 studies met the eligibility criteria, including 56 empirical and 21 nonempirical studies. Eleven validated screening instruments were identified, most frequently the SCOFF followed by the EDE-Q. Most studies used self-administered instruments before the consultation, with completion times ranging from 30 seconds to 15 minutes. Implementation strategies commonly involved the provision of materials and training for health professionals. Instrument robustness was primarily assessed through validity and reliability measures. The EDS-PC, EAT-26 and ADO-BED showed the highest sensitivity (100%), while the SCOFF demonstrated the highest specificity (94.4%). Feasibility was investigated through the identification of cases, team actions, professionals’ perceptions, and barriers and facilitators to implementation. Despite the potential of several instruments, robust evidence on their validity, reliability, and applicability remains limited, particularly in low-and middle-income settings, among underrepresented and adolescent’s populations. Structural and training barriers hinder the effective implementation of screening, highlighting the need for articulated actions between professionals, services, and public policies.

## 1. Introduction

Eating disorders (ED) are severe, complex, and often chronic psychiatric conditions characterized by persistent disturbances in eating patterns or eating behavior [1]. Although these conditions can affect individuals of all ages, genders, sexual orientations, ethnicities, or regions, adolescents and young women represent the highest risk group and are the third most common chronic condition among adolescents [2,3]. Although underreported, data from the 2019 *Global Burden of Disease* study estimate that approximately 55.5 million people in the worldwide lived with ED that year [4]. Overall lifetime prevalence rates range from 2.6% to 8.4% in women and 0.7% to 2.2% in men and may be underestimated, especially in view of the worsening observed during the COVID-19 pandemic [5–7]. Anorexia nervosa (AN) affects approximately 4% of women over their lifetime, whereas it affects 0.3% of men [8]. Bulimia nervosa (BN) affects approximately 3% of women and 1% of men, whereas binge eating disorder (BED) affects, on average, 1.4% of women and 0.4% of men [8,9].

ED also stands out for its high mortality rates, including increased risk of suicide, as well as social and economic impacts [10–13]. Despite the significant impacts of ED, only approximately 23% of people seek treatment from qualified professionals [14]. An average delay of more than five years is estimated between the onset of symptoms and access to appropriate care, which varies according to the type of disorder [15,16]. Screening can help reduce this interval, minimizing clinical complications, mortality, and the burden on the health system [13,17]. Screening is understood as a process aimed at the early identification, in apparently healthy populations, of people at increased risk for certain health conditions [18]. However, for ED screening to be effective, it is important to use appropriate instruments and act in strategic contexts, with primary health care (PHC) being a promising setting. It has been suggested that people with ED tend to seek this level of attention more frequently than others in the years leading up to diagnosis [19]. Although there are several screening instruments, ranging from long versions to short scales, it is essential to consider their psychometric properties, ensuring that they are validated for the target population and effective in the early identification of risk behaviors [19]. In addition, it is important to identify and understand the methodological approaches adopted in its application, since many PHC professionals report little or no training on ED and face multiple barriers during the identification process [19].

To our knowledge, six systematic reviews [20–25] on ED screening with specific objectives and covering different health care settings have been identified. Although Kalindjian et al. [13] conducted a recent scoping review, their focus was on the early detection of ED in settings other than the one proposed here, including studies conducted in schools, specialized clinics, and primary care. In contrast, our review broadens the spectrum of analysis by exploring not only the characteristics of ED screening strategies but also the methods of assessing the robustness of these strategies and their feasibility in PHC. In this way, we seek to identify gaps in existing knowledge and offer relevant contributions to clinical practice.

The aim of this review is to map the extent of the available evidence on validated strategies for ED screening in adolescents and adults assisted by PHC. Four research questions (RQs) were formulated to guide the analysis: (RQ1) In which geographic, demographic, sociocultural, and economic contexts are the validated screening strategies used? (RQ2) What are the characteristics of the validated strategies for ED screening and their application (who conducted the screening, when, how, where it was performed, the frequency of use, and the duration of application) in the context of PHC? (RQ3) What evaluation methods are used to determine the robustness of ED screening strategies? (RQ4) How can the feasibility of applying ED screening strategies in PHC be assessed, considering factors such as the proportion of patients accepting screening, case identification, actions taken by the team, the barriers and facilitators encountered during the implementation of the strategies, and the perceptions/experiences and knowledge of professionals?

## 2. Materials and methods

This scoping review followed the guidelines of the Preferred Reporting Items for Systematic reviews and Meta-Analyses extension for Scoping Reviews (PRISMA-ScR) [26] (S1 Appendix). A research protocol was developed according to the methodological recommendations of the Preferred Reporting Items for Systematic Reviews and Meta-Analyses Protocols (PRISMA-P) [27,28] and the Joanna Briggs Institute (JBI) [29], which has been registered in the Open Science Framework (https://osf.io/498fu/) and previously published by De Santana et al., 2024 [30].

### 2.1 Eligibility criteria

Table 1 presents the eligibility criteria according to the PCC model (participants, concept, context) established for this scoping review. Studies covering different health services were included when the results were presented separately by type of service. Thus, only data referring to PHC were extracted and analyzed.

**Table 1:** Inclusion and exclusion criteria based on the Participant Concept Context (PCC) framework.

	Inclusion criteria	Exclusion criteria
**Participants**	• Studies including adolescents (10–19 years) and adults (≥20 years) of any sex or gender identity.• Studies involving health care professionals working in primary health care.	• Studies including individuals under 10 years of age.• Studies conducted exclusively in primary care settings.
**Concept**	• Studies that included screening for eating disorders or disordered eating using validated instruments.• Studies assessing screening tools in primary health care settings.	• Studies that included screening for avoidant/restrictive food intake disorder (ARFID), pica, rumination disorder, or mental disorders such as depression or anxiety.• Studies that did not evaluate screening instruments.
**Context**	• Studies conducted in primary health care settings worldwide. • Studies conducted in different economic and sociocultural contexts, including ethnic and racial minority groups.	• Studies conducted in other health care settings, such as hospitals, outpatient clinics, and specialized eating disorder services.

### 2.2 Sources of information and search

To minimize any bias in the selection of studies and to identify as much relevant evidence as possible, the search was conducted in six databases [MEDLINE (PubMed), Embase, *Latin American and Caribbean Health Sciences Literature* (LILACS), Web of Science, PsycINFO, *Cumulative Index to Nursing and Allied Health Literature* (CINALH) and gray literature (*ProQuest Dissertations and Theses Database and Google Scholar*)] until October, 2024. There was no limitation on language or year of publication. The search strategy was developed with the help of a health librarian and was carried out in three stages, as recommended by Peters et al. (2020) [29]. Initially, a preliminary search was carried out in MEDLINE (PubMed) to identify words contained in the title, abstract and keywords. Additionally, controlled vocabularies Medical Subject Headings (MeSH), Embase Subject Headings (EMTREE) and Health Sciences Descriptors (DECS) were used. All the words and descriptors identified were subsequently used to construct the search strategy for MEDLINE (PubMed). This strategy has been adapted to other databases.

In addition, ten of the most relevant experts in the field, identified on the http://expertscape.com website using the term “eating disorders,” were contacted to suggest relevant studies not captured by electronic searches. Finally, a manual search of the references of the included studies was conducted. The final search strategy for MEDLINE and other databases can be found in S2 Appendix.

### 2.3 Selection of studies

Two independent reviewers performed the selection, and any disagreements were discussed with a third reviewer. A pilot trial with 50 randomly selected studies was conducted with the aim of calibrating the screening process between reviewers. The reviewers started the selection after confirming agreement, which was 75% [29].

Next, the citations retrieved from each database were exported to *Covidence* (www.covidence.org) online software, and the selection of studies began. After duplicate records were deleted, screening was conducted on the basis of the titles and abstracts of the retrieved sources. The eligibility criteria were subsequently verified by reading the full text. All excluded sources were recorded along with the reason for deletion (S3 Appendix).

### 2.4 Process of mapping data and data items

The entire data extraction process was carried out in the online software *Covidence*. The items for data extraction were adapted from JBI (2020) [29], and a pilot test was conducted with five studies randomly selected by the reviewers. The extracted data included the main characteristics of the studies, the screening strategy adopted (instrument and/or methodological approach), methods used to assess the robustness of the strategy and the feasibility of the screening strategy, among other information relevant to this review. Two pairs of independent reviewers extracted the data. Any disagreement between each pair was resolved through discussion or, if necessary, with the mediation of a third reviewer. In addition, the authors of the included studies were contacted to obtain relevant information that was not clear or was missing from the publication. In cases of nonresponse, data that could not be retrieved were marked as missing or not reported.

### 2.5 Summary of results

The synthesis of the results was conducted through a narrative approach, characterized according to the PCC model and grouped into categories according to the questions in this review. The included studies were classified as empirical (original studies) or nonempirical (reviews), and the data were organized in tables and figures. The quantitative data were analyzed via frequencies and absolute values. For the qualitative studies and mixed-methods studies, a thematic categorical analysis was carried out, identifying the main barriers and facilitators for ED screening in PHC. The analyses and figures were prepared with the support of Microsoft Office (www.office.com), CorelDrawn (www.coreldraw.com), GQIS (https://qgis.org/) and Canva (www.canva.com).

### 2.6 Differences from the protocol

During this review, a modification was made in relation to the previously published protocol (DOI 10.17605/OSF. IO/498FU). Initially, the review focused only on participants who used PHCs in which ED risk screening instruments were applied. However, we expanded the criteria to include health professionals to identify the methodological approaches used, as well as the feasibility of applying screening strategies.

## 3. Findings

### 3.1 Selection of sources of evidence

**Fig 1:**
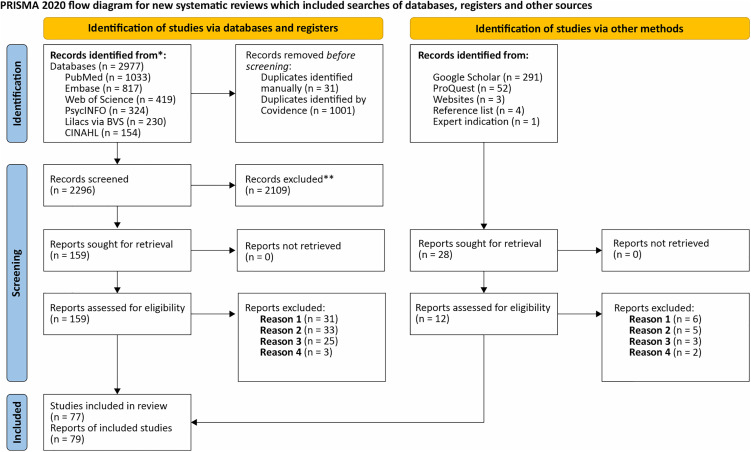
Literature search flow diagram and selection criteria. Reasons for exclusion: Reason 1: Letters, book chapters, conference abstracts, opinion articles, clinical guides, study protocols, guidelines, comments, and news. Reason 2: Articles that did not mention validated screening strategies for ED or the feasibility of their application. Reason 3: Articles conducted in primary health care settings or in generalizable settings. Reason 4: Articles that did not include adolescents and adults or primary health care professionals in the sample and those that included individuals already diagnosed with ED. Source: Page et al. *The PRISMA 2020 statement: an updated guideline for reporting systematic reviews.* BMJ 2021; 372: n71. For more information, please visit: http://www.prisma-statement.org/.

A total of 2977 documents were recovered from the six databases. After the removal of duplicates, 2296 documents were sorted by title and abstract, resulting in the selection of 159 for reading the full text. Of these, 65 studies were included, two of which had two published articles each [32–35]. In addition, searches in gray literature, manuals, *websites*, and consultations with experts resulted in 28 citations for reading the full texts, of which 12 met the inclusion criteria. Finally, 108 citations were excluded (S3 Appendix), and 77 studies were included in this review: 56 empirical [31–87] and 21 nonempirical [13,21,23,25,88–104] (Fig 1).

### 3.2 Bibliometric analysis and characteristics of evidence sources (RQ1)

**Fig 2:**
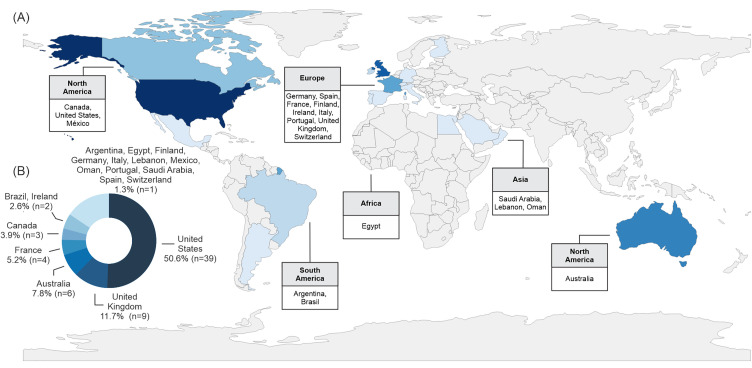
Geographic distribution of included studies. (A) Worldwide distribution of selected studies on validated screening strategies for ED across North America, South America, Europe, Oceania, Asia, and Africa (n = 77) (empirical and nonempirical studies); (B) Prevalence of included studies distributed by country (n = 77). The map was created using QGIS software. Base map data were obtained from Natural Earth (1:110m scale), which is in the public domain. Study data were extracted from the articles included in this scoping review.

**Fig 3:**
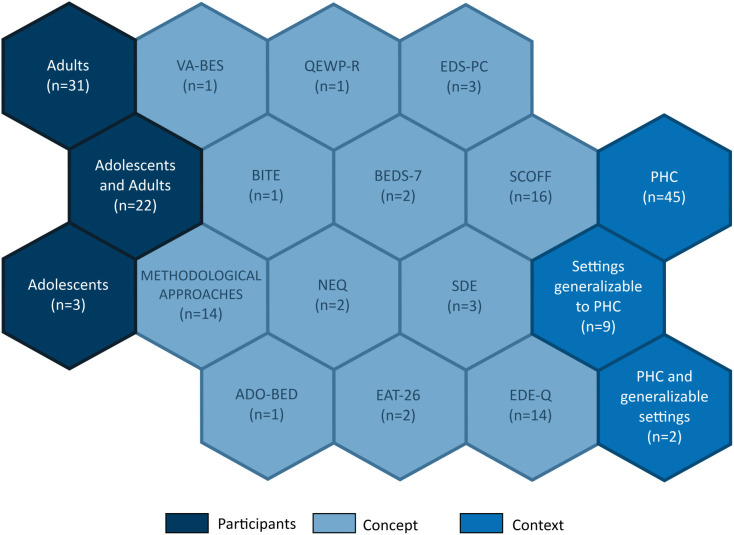
Visual representation of the results of the scoping review and the number of relevant studies by participants, concept and context. Acronyms: ADO-BED: Binge Eating Disorder Questionnaire in Adolescents; BEDS-7: Screening Instrument for Binge Eating Disorder; BITE: Edinburgh Bulimia Investigative Test; EAT-26: Eating Attitudes Test; EDE-Q: Eating Disorders Examination Questionnaire; EDS-PC: Eating Disorders Screening for Primary Care; NEQ: Questionnaire on Night Feeding; QEWPR: Questionnaire on Eating and Weight Patterns; SCOFF: Sick, Control, One Stone, Fat, Food; SDE: Screening for Disordered Feeding; VA-BES: Veterans Affairs Binge Eating Screening.

The main characteristics of the included studies are presented in Figs 2 and 3 and S1 and S2 Tables. Nineteen countries distributed on five continents were included in the studies analyzed (Fig 2). Of the 56, half (50%/ n = 28) were conducted in North America [31–35,46,50,51,59–66,68–73,75–77,82,84–87]. Europe accounted for 30.4% (n = 17) of the empirical studies [38,43,44,47–49,52,53,55–57,74,78–81,83], followed by Oceania with 7.1% (n = 4) [42,58,67,105], and South America with 5.4% (n = 3) [39,41,45]. Finally, Asia and Africa accounted for 5.4% (n = 3) [37,40,54] and 1.8% (n = 1) [36] of the empirical studies, respectively. Among the 21 nonempirical studies, 71% (n = 15) were conducted in North America [21,23,89–92,94–100,102,104], 19% (n = 4) in Europe [13,88,93,103] and 10% (n = 2) Oceania [25,101].

The included studies presented different methodological designs, with a predominance of cross-sectional designs (n = 27), narrative reviews (n = 16) and mixed-methods studies (n = 14). The studies were published between 1989 [52] and 2024 [36,37,58,63].

Among the 56 empirical studies, the sample size ranged from five [61,86] to 1,971 participants [39], and 80% of these studies adopted nonprobability sampling. In all, the reviewed studies included 14,580 participants, including PHC users (24 studies) and health professionals (25 studies), while seven included both groups. The ages of the participants who used PHC ranged from 10–64 years, and those of the health professionals ranged from 18–69 years. Most empirical studies (n = 35) included only women in their sample or showed a predominance of female participants among PHC users and professionals. One study included transgender people [46], and another explicitly reported participants’ sexual orientation [105]. In 11 studies that assessed racial self-reporting, the majority of participants, both users and professionals, identified as white/Caucasian [34,35,39,46,50,51,57,59,68,70,73,77]. On the studies including PHC users, four revealed that the majority of users were married [49,50,52,54] and had low to intermediate family income (monthly income between $1000 and $2600 USD) [37,41,45,54], whereas three studies reported that the most participants had nine or more years of education [26,27,76]. Of the studies included, 32 involved PHC professionals. Among these, 16 studies predominantly involved physicians, while other health professional categories were underrepresented [58,63,69,72,74–81,83–86]. For more details on demographic and socioeconomic characteristics, see S1 Table.

Regarding study settings, most studies (n = 45) were conducted exclusively in PHC, while nine studies in environments generalizable to PHC, such as university-affiliated services and hospital-affiliated services, and two combined both contexts. In terms of location, 31 studies were conducted in urban and metropolitan areas (S1 Table).

### 3.3 Results of individual sources of evidence and synthesis of data

#### 3.3.1 Empirical research studies.

3.3.1.1 *Characteristics of the strategies and their implementation (RQ2)*

Methodological tools and approaches. Of the 56 empirical studies, 11 different validated instruments were identified. The Sick, Control, One Stone, Fat, Food (SCOFF) questionnaire was used in 16 studies [32,33,40,48–51,53–57,60,63,64,66,67], followed by the Eating Disorder Examination Questionnaire (EDE-Q), which was used in 14 studies [32,34,37–39,41–43,45,46,50,51,67,105] (n = 14) (Fig.3). The number of items in the instruments ranged from one item in the Veterans Affairs Binge Eating Screening (VA-BES) to 33 in the Edinburgh Bulimia Investigative Test (BITE). Most of the scales analyzed use scoring systems based on dichotomous answers (yes/no), such as Questionnaire of Binge Eating Disorder in Adolescents (ADO-BED), Screening Instrument for Binge Eating Disorder (BEDS-7), Eating Disorders Screening for Primary Care (EDS-PC), SCOFF, Screening for Disordered Feeding (SDE), and VA-BES. Most of the screening was performed by researchers through self-administration of the instruments before the consultation, with completion times ranging from 30 seconds to 15 minutes. Table 2 presents the characteristics of each instrument and its application.

**Table 2:** Characteristics of the screening instruments and their application.

Instrument	No. of items (cutoff point)	Accountable	When	How	Local	Time
ADO-BED [47]	10 (NA)^a^	Researcher	After the consultation	Self-administration^b^	NR	NR
BEDS-7 [31]	7 (NA)	Healthcare professionals	During the consultation	Self-administration	Office	NR
BITE [44]	33 (≥10)	Researcher	Before the appointment	Self-administration	Waiting room	NR
EAT-26 [32,52]	26 (≥20)	Researcher [32.52]	Before the consultation [52]	Self-administration [32,52]	NR	5.15 (±0.31) min. [32]
EDE-Q [32,34,37–39,41–43,45,46,50,51,67,105]	28 (≥2.3, ≥2.8 or ≥4.0)	Trained researcher or interviewer [4,32,34,38,41,42], health care workers [37,39,43,46], receptionist [50], via mail [42,43]	Before or after the consultation [41] before the consultation [42,43], during the consultation [37,46], in a group session [34]	Self-administration [32,34,38,39,42,43,46,50,51,105], self-administration and heteroadministration in specific cases [39]), heteroadministration [37,41,45]	Waiting room [42,43], office [37], home [45,51,105], and examination room [46]	2–4 min [50] to 15.23 (±1.23) min [32]
EDS-PC [51,53]	4 (≥2)	Via mail [51], psychiatrist [53]	Before the consultation [53]	Self-administration [51], heteroadministration [53]	Home [51], waiting room [53]	NR
NEQ [35,36]	14 (≥25)	Family physician [36], researcher [35]	During weight management group sessions [35]	Self-administration [35], heteroadministration [36]	NR	NR
QEWP-R [34]	28 (NA)	Researcher	During weight management group sessions [34]	Self-administration	NR	NR
SCOFF [32,33,40,48–51,53–57,60,63,64,66,67]		Researcher [32,33,40,48,49,53,57,64], nurses [56], psychiatrist [53], ED specialists [54], receptionist and health professionals [50,63], via mail [51]	Before or after the consultation [49], before the consultation [53,57,63,64,66] and during the consultation [54,56]	Self-administration [32,33,40,48–51,53–57,63,64,66], heteroadministration [53]	Waiting room ^[53.64]^, office [54], separate room [48], quiet environment outside the waiting room [57], home [51], reception and office [63]	30s [50] to 3.98 min. (±0.12) ^[32.33]^
SDE [51,62,67]		Via mail [51], assistance from the nursing team [62]	NR	Self-administration [51]	Participant’s domicile [51]	NR
VA-BES [34]		Researchers	During weight management group sessions [34]	Self-administration	NR	NR

a: Self-administration: instrument answered directly by the participant himself, without the mediation of an evaluator.

b: Heteroadministration: instrument applied by a professional or researcher, which reads and records the participant’s answers.

c: age under thirteen years and/or comprehension difficulties in adolescents

ADO-BED: Questionnaire of Binge Eating Disorder in Adolescents; BEDS-7: Screening Instrument for Binge Eating Disorder; BITE: Edinburgh Bulimia Investigative Test; EAT-26: Eating Attitudes Test; EDE-Q: Eating Disorders Examination Questionnaire; EDS-PC: Eating Disorders Screening for Primary Care; NA: Not applicable; NR: Not Reported; NEQ: Questionnaire on Night Feeding; QEWPR: Questionnaire on Eating and Weight Patterns; SDE: Screening for Disordered Feeding

Of the total number of empirical studies, 14 investigated methodological approaches to assisting health professionals in screening for ED [58,60–67.69–72,87]. These methodologies included the implementation of screening instruments in clinical practice through the provision of materials (e.g., referral instruments and protocols) and/or training focused on the role of PHC professionals in screening.

These approaches were conducted either in person or remotely, including video presentations lasting between 10 [61] and 75 minutes [71]. Quantitative and qualitative questionnaires were administered before or after the interventions for evaluation purposes. Approaches focused on training health professionals are fundamental for enhancing and enabling screening. Among these 14 studies, eight reported improvements in knowledge [58,60,61,65,66,69–71], two increased comfort [66,70] and one improved skills of professionals [87]. One study also reported better understanding of referrals processes [66]. The participants positively evaluated the feasibility of the training for their clinical practice [58], reported satisfaction [69] and highlighted the importance of systematizing referrals [87] (Table S1).

*3.3.1.2 Methods for assessing the robustness of instruments (RQ3).* Considering the robustness of the instruments, which were evaluated on the basis of psychometric measures, Table 3 presents a summary of the main characteristics of reliability and validity. Owing to the greater number of studies that have evaluated SCOFF and EDE-Q, these instruments will be addressed with greater prominence below.

**Table 3:** Psychometric properties of ED screening instruments in PHC.

Instrument	Reliability	Se (%) (95% CI)	Sp (%) (95% CI)	PPV (%) (95% CI)	VPN (%) (95% CI)	AUC (95% CI)
ADO-BED [47]	NR	100 (NR)	27.4 (NR)	28.4 (NR)	100 (NR)	NR
EAT-26 [52]	NR	100 (NR)	91 (NR)	NR	NR	NR
EDE-Q [32,34,37–39,41–43,45,46,50,51,67,105]	α of Cronbach: 0.74- 0.96	80 (NR) – 99.3 (96.2-100)	80 (NR) – 91.7 (88.3-95.1)	44 (NR)	NR	0.85 (NR)
EDS-PC [51,53]	NR	96.6 (88.1–99.6) – 100 (90-100)	40,3 (35.0–45.8) - 71 (64-77)	22.1 (17.2–27.8)	98.5 (94.8–99.8)	0.684 (0.649–0.720)
SCOFF [32,33,40,48–51,53–57,60,63,64,66,67]	Cronbach’s α: 0.725 – 0.44 ICC: 0.97 (0.96–0.98).	65.2 (NR) - 97.7 (93.5–99.5)	72.7 (62.6-80.9) - 94.4 (86.4–98.5)	50 (NR) – 81 (74 - 86.8)	92.8 (89.2–95.5) – 99.6 (98.6–100)	0.730 (0.667–0.793) - 0.947 (NR)
SDE [51,62,67]	NR	90.5 (80.4–96.4)	57.5 (52.1–62.8)	28.4 (22.2–35.1)	97.0 (93.6–98.9)	0.740 (0.695–0.78)
VA-BES [34]	NR	88.9 (NR)	83.2 (NR)	30.8 (NR)	98.9 (NR)	NR

^a^: the minimum and maximum values of Se, Sp, PPV, NPV, AUC found in the studies

ADO-BED: Questionnaire of Binge Eating Disorder in Adolescents; AUC: Area under the ROC curve; ICC: Intraclass Correlation Coefficient; EAT-26: Eating Attitudes Test; EDE-Q: Eating Disorders Examination Questionnaire; EDS-PC: Eating Disorders Screening for Primary Care; NEQ: Questionnaire on Night Feeding; NR: Not Reported; QEWPR: Questionnaire on Eating and Weight Patterns; SDE: Screening for Disordered Feeding; If: Sensitivity; Sp: Specificity; VA-BES: VA Binge Eating Screening; NPV: Negative Predictive Value; PPV: Positive Predictive Value.

The reliability of SCOFF was determined in three studies [33,50,54], with *Cronbach’s α* ranging from 0.43–0.725 and an intraclass correlation coefficient (ICC) of 0.97. The validity of the SCOFF questionnaire was assessed in eight studies [32,33,48–51,53–55]. Of these, two analyzed the internal structure using Confirmatory Factor Analysis (CFA) and Principal Component Analysis (PCA), whereas eight focused on criterion validity, using the cutoff point = 2. The AFC demonstrated an excellent correlation between the identified factors and the overall score (r = 0.98, P < 0.001) [54]. PCA, on the other hand, concludes that SCOFF can measure both a single construct and specific dimensions [56]. In addition, only one study performed four stages of cultural adaptation (translation, back-translation, pilot study, and review of the final version by the authors of the original version) [55], three studies performed cross-cultural adaptation of the tool through two or more steps (translation, back-translation, and pilot study) [37,54,56], and one study made minor changes to the original version to adapt it to its population [50].

The reliability and validity of the EDE-Q were evaluated in two studies; however, cutoff points below those determined by the original scales of 2.3 and 2.8 were used [32,50]. In addition, two studies performed cultural adaptation through translation alone [32,39].

With respect to the other instruments, one study translated the ADO-BED [47]. The instruments with the highest sensitivity (100%) were the EDS-PC [53], EAT-26 [52] and ADO-BED [47] instruments. The highest specificity (94.4%) and PPV (positive predictive value) (81%) were presented by SCOFF. The ADO-BED presented the highest NPV (negative predictive value), with 100% [47]. With respect to the discriminative capacity, as evaluated by the AUC (area under the curve), the SCOFF presented a value of 0.947 [33], and the EDS-PC presented a value of 0.684 [51].

3.3.1.3 *Feasibility of screening (RQ4)*

*Screening acceptance rate, case identification, and actions taken by staff.* Of the 56 empirical studies, 16 reported the acceptance rate of the screening instrument [38,40,44,45,47,49–52,54–57,62,63,105], ranging from 14.1% for the SDE [62] to 100% for the EDE-Q [38] and the SCOFF [40,54,55,63]. The prevalence of ED behavior ranged from 0.2% in pregnant women with an EDE-Q global score ≥ 4 (41) to 55.4% in adolescents and adults with a SCOFF score ≥2 [40]. Among the empirical studies, 12 that analyzed the actions of health teams after positive screening for ED reported that most conducted clinical interviews [32,33,39,42,105] or referred patients for care [62,63,66,67]. Other results are presented in Table S1.

*Perception, experience and knowledge of professionals about ED screening.* Professionals’ perceptions of ED screening were assessed in 15 studies [57,59,60,62–66,69,73,77,79,82,85,87]. Of these, seven studies reported positive evaluations of the screening and the instruments used, highlighting its ease, usefulness, efficacy, and practicality [57,59,62–66,77]. Five studies investigated the confidence of professionals as responsible for screening [59,60,66,79,85], with rates ranging from 25% [79] to 54% [60] among professionals. The perception of comfort in performing screening was analyzed in four studies [57,64,69,87], with the proportion of participants who reported it ranging from 33% [64] to 100% [69]. Only one study addressed the perceived ability of professionals in ED screening, indicating that the majority (62.2%) considered their competence low or very low to perform the procedures [87]. Two studies evaluated the perceptions of professionals in relation to universal screening, that is, for all individuals who sought care regardless of the main complaint. In these studies, 40% [87] to 54% [73] of professionals recognized the importance of adopting this strategy in primary care.

The experience of professionals in screening was investigated in 11 studies [59,60,64,69,72,76,80–82,86,87] (Table S1). The proportion of PHC professionals who reported being screened for ED ranged from 12.1% [59] to 43.0% [60]. The frequency of screening varied between studies, being predominantly rare or nonexistent in three of them [64,81,87]. In four other studies [61,65,79,82], most professionals reported not using screening instruments in their routine. The screening practices used varied according to gender and professional category. In one study, female professionals showed a greater tendency to adopt the screening questionnaire to the detriment of the evaluation of clinical signs [80]. Similarly, another study reported that nurses perform more thorough evaluations than physicians do [75].

Four studies [61,66,82,83] evaluated professionals’ knowledge about ED screening in PHC. In general, knowledge was classified as low/nonexistent [61,83] to moderate [66]. With respect to knowledge of the instruments, the levels were also low, ranging from none [61,83] to 24% [82] of the professionals reporting some knowledge.

*Barriers and facilitators for ED screening in PHC.* The barriers faced in ED screening by professionals were evaluated in 19 studies [57,59,61,64–68,73,74,77,78,81–87]. Most studies indicated insufficient time to perform screening (n = 14) [57,59,61,65–67,73,74,77,83–86]. Limited knowledge about ED and its screening was reported in 11 studies [59,61,64,68,73,74,77,78,82–84], whereas challenges in the referral of positive cases were highlighted in six studies.

Facilitators of ED screening by professionals were evaluated in four studies [65,67,73,87]. All studies reported the availability of a screening instrument as a facilitating factor, being considered a comfortable means of starting conversations on the topic [67]. Additionally, in two studies [67,73], the presence of a structured referral was reported. The presence of questions in the instrument aligned with the scope of the medical professional, as did specific training for the ED and the existence of a multidisciplinary team for treatment, which were considered potent facilitators in one study (Table 4 and S1 Table) [73]. No studies were found with patients’ self-reports on barriers and facilitators.

**Table 4:** Main barriers and facilitators to screening reported by professionals.

	Emerging topics*	Studies reporting the theme, n (%)
**Barriers (n = 19)**	Insufficient time	14 (73.7)
	Communication issues and lack of trust	12 (63.2)
	Limited knowledge	11 (57.9)
	High workload and competing priorities	9 (47.4)
	Inadequate training; Lack of experience; Uncertainty about effectiveness; Lack of motivation	9 (47.4)
	Lack of systematic screening; Absence of specific tools; Financial, administrative, and geographical constraints; Unclear role; Fear of overstepping scope	8 (42.1)
	Referral challenges	6 (31,6)
	Sociocultural issues; Weight management; Low patient demand	6 (31,6)
	Feelings of powerlessness and discomfort	3 (15,8)
	
**Facilitators (n = 4)**	Screening tools;	4 (100)
	In-scope questions; Training on ED; Multidisciplinary team	3 (75)
	Structured referral process	2 (50)

*Frequencies are represented as n (%) based on 23 studies reporting barriers (n = 19) and facilitators (n = 4). Studies could report more than one barrier or facilitator; therefore, categories are not mutually exclusive.

#### 3.3.2 Nonempirical research studies.

A total of 21 reviews were included, of which 16 were narratives, three were systematic, one was systematic with meta-analysis, and one was a scoping review. Narrative reviews recommended the use of SCOFF [89,94–97,99,100,102,104], but highlighted its limitation in detecting all cases of ED, especially outside AN and BN [89,94,101]. In this sense, the QEWPR-5 and BEDS-7 are considered useful instruments, as they encompass the diagnosis of ED [89–92,94,100,104]. The EDE-Q [90,92,100,102,104], EAT-26 [94,99], EDDS (Diagnostic Scale of Eating Disorders) [94,104] and ESP-PC [90,95,100] were also cited as applicable to PHC.

Reviews also emphasize the importance of screening for ED in adolescents, recommending annual forms such as *Bright Futures* [98,102] and *Guidelines for Adolescent Preventive Services* [98]. Instruments such as the ADO-BED [102] and the *Ottawa Disordered Eating Screen for Youth* (ODES-Y) [97] have been indicated to screen for ED in adolescents with obesity and atypical AN, respectively. Some narrative reviews have proposed strategies to qualify screening, such as training PHC professionals and the use of tools [88,101]. These initiatives aim to overcome common barriers to the early detection and intervention of ED, as reported in four reviews [88,92,100,101].

Systematic reviews [21,23,25,103] highlighted the relevance of the instruments, with an emphasis on SCOFF, which has been widely evaluated for its high sensitivity and specificity. In addition, these reviews identified gaps in the effectiveness of currently available instruments, as they fail to detect all ED owing to population diversity and symptom overlap.

A scoping review [13] highlighted the role of family physicians, dentists, and gynecologists in the early detection of ED in PHC. Interventions aimed at training health professionals have been effective in improving the detection of ED. Details on the main results of the nonempirical research studies are presented in S2 Table.

## 4. Discussion

### Summary of evidence

This scoping review offers a comprehensive synthesis of the scientific literature on ED screening strategies in adolescents and adults in PHC, bringing together 56 empirical and 21 nonempirical studies published between 1989 and 2024. Several validated instruments were identified, with emphasis on the SCOFF and the EDE-Q, which are used in various geographical, sociocultural, and economic contexts, although with a predominance of studies in North America and in white and female populations. The psychometric properties generally indicated good indicators of sensitivity and specificity. In addition, methodological approaches such as the capacity building of health professionals are important for improving knowledge, confidence, and skills in the implementation of ED screening. On the other hand, barriers such as insufficient time in consultations, lack of knowledge and difficulties in referral flows still limit the implementation of screening. The reported facilitators involved the provision of simple instruments, structured protocols, and multiprofessional support. The results reinforce PHC as a strategic setting for the early detection of ED, especially in contexts of high demand and limited access to specialized services.

In general, the literature reveals a complex panorama of ED screening strategies in PHC. Although instruments such as SCOFF are valued for their practicality and quick administration, they may have limitations in fully capturing the entire spectrum of ED [25,101]. Thus, its use may be appropriate as an initial stage of screening and should be complemented by more detailed instruments, such as the EDE-Q, which is considered the gold standard [50]. Despite its ability to be extended, the EDE-Q has good sensitivity and specificity and is more suitable for more in-depth and subsequent investigations [50]. Notably, the diversity of symptomatic manifestations of ED makes it difficult to use a single instrument capable of covering the entire spectrum of these conditions. In this context, the use of screening strategies should be understood as initial support, without replacing qualified clinical listening.

Even so, these tools have limitations in terms of diagnostic accuracy, being subject to false positives and missed cases [21]. While false positives may lead to unnecessary referrals, false negatives may delay identification and timely access to care. Additionally, another critical issue concerns the cultural biases present in screening instruments, which tend to privilege cultural stereotypes of gender, race, and social class, for example, the profile of white women from the middle and upper class background while a neglecting populations experiencing greater social vulnerability, such as individuals in situations of food insecurity [106].

Overall, both the reliability of SCOFF and EDE-Q were considered optimal (Cronbach’s alpha > 0.70) [107], although one study reported low reliability for SCOFF, with a Cronbach’s alpha of 0.44 [50]. In addition, the sensitivity values, the most important characteristic of the screening instruments, were considered satisfactory (>80%) [108]. With respect to discriminative ability, as assessed by the AUC, SCOFF showed excellent performance (AUC = 0.947), whereas EDS-PC demonstrated reasonable ability (AUC = 0.684) [108]. Notably, SCOFF was the only instrument with studies evaluating both content and construct validity [54,56], of which two specifically analyzed these aspects. With respect to ED screening by means of a cutoff bridge of the instruments, the use of different values for the EDE-Q can generate variations in the identification of potential ED cases. In the original version of the instrument, the cutoff point for the global scale was ≥ 4 [109]; however, *Mond* et al*.* (2004) [110] proposed a cutoff point of 2.3 for a community sample of adult women. *Mond* et al*.* (2008) [50] suggested a cutoff point of 2.8 in a sample of PHC users. Such differences reflect efforts to adapt the instrument to the particularities of different population contexts.

Another important aspect concerns the cross-cultural adaptation of the instruments, which is essential for ensuring their applicability in different sociocultural contexts [110]. Considering that ED manifests itself differently across age and cultural groups, it is all the more necessary to carry out appropriate adaptations, respecting all stages of the adaptation process. Borsa et al. (2022) [111] proposed six steps for the cross-cultural adaptation process: (1) translation into the target language, (2) synthesis of versions, (3) evaluation by expert judges, (4) evaluation by the target audience, (5) reverse translation, and (6) pilot study. However, many studies have not covered all these stages, often limiting themselves to the stages of translation and back-translation, which can compromise the validity and cultural adaptation of the instruments used. Furthermore, the SCOFF was the only instrument whose internal structure was evaluated through confirmatory factor analysis and principal component analysis [54,56].

With respect to the feasibility of screening strategies in PHC, the presence of facilitators was identified, such as the adoption of instruments for the identification of ED. However, there is considerable heterogeneity in the perceptions, knowledge and experience of professionals in regarding screening practices. Gender differences in attitudes toward the use of screening questionnaires were observed in the study by Boulé McSherry (2002). The findings suggest that female professionals were more likely than their male counterparts to adopt screening strategies based on standardized instruments [80]. However, the factors underlying these differences remain unclear. Similar findings have been reported in other areas of mental health-related screening. A study on substance use screening during pregnancy found that female obstetricians/gynecologists were more likely to believe in the effectiveness of screening and to adopt standardized screening tools in clinical practice [112].

Several barriers were identified, including the limited time of consultations, lack of knowledge about ED, and the absence of structured referral flows for positive cases. In this sense, the importance of training for the proper management of instruments and for the referral of cases is highlighted, as well as the creation of lines of care that begin in PHC and involve various levels of health care. In addition to overcoming these barriers by professionals, ensuring that individuals with ED referred to specialized services have access to appropriate treatment and that these services have the necessary structure to welcome them is essential. Therefore, screening alone is insufficient to guarantee the quality of mental health care.

Among the main strengths of this review, the following stand out: (I) the breadth and scope of the bibliographic search, conducted in several databases, and complemented by a search in the gray literature, manual analysis of references and consultation with specialists, without geographical or temporal restrictions; (II) the rigorous, transparent and systematic application of the scoping review methodology; and (III) the adoption of comprehensive inclusion criteria, which allowed the incorporation of previously published empirical and nonempirical studies on the subject. Another highlight is that the review protocol was registered with OSF and previously published [30], which reinforces the methodological rigor and transparency of the process.

### Limitations and gaps identified

Despite these important contributions, this review has several limitations. As this is a scoping review, we have not conducted a quality assessment on the basis of risk of bias, and no critical quality assessment has been conducted. In addition, the results of a scoping review are descriptive, and it is not possible to answer more in-depth questions about specific aspects. However, considering the methodological rigor adopted here, we believe that our results provide important information on ED screening in PHC and that ours is the most comprehensive scoping review of which we are aware to date, integrating a wide range of studies and methodological designs.

The results demonstrate important gaps in the literature. For example, most of the studies included were carried out in North America and in high-income countries, with populations predominantly white women, limiting the applicability of the results to other sociodemographic groups. It is necessary to carry out more detailed investigations on screening strategies in adolescents and adults from different sociocultural contexts, such as men, black populations and LGBTQIAPN+ individuals, as well as those belonging to lower economic classes. In addition, in studies involving professionals, there was little representation of professional categories other than physicians, who can also perform screening in PHC and favor the strengthening of the team and decentralization of actions.

Our results also reveal a lack of research evaluating the psychometric properties of screening instruments in the context of PHC. Although the evidence cited eleven validated instruments, only seven had psychometric properties analyzed in PHC. A gap was also observed in the evaluation of these properties in exclusive samples of adolescents. Only one study evaluated SCOFF and EDE-Q in this population, with sensitivities of 91.6% and 99.3%, respectively [32]. These data reinforce the need for investigations that consider this population exclusively in the validation of screening instruments.

However, the findings of this scoping review point to the feasibility and relevance of conducting future systematic reviews, especially those aimed at evaluating the psychometric properties of EDs screening instruments in primary health care.

## Conclusion

This scoping review provides a comprehensive overview of validated ED screening strategies in PHC, identifying the available instruments, their application characteristics, psychometric properties, methodological approaches, and aspects related to feasibility. Despite the potential of some instruments for use in PHC, there is still a lack of robust evidence on their validity, reliability, and applicability in different contexts, especially in adolescent populations. Although there are advances, structural and training barriers hinder the effective implementation of screening, which highlights the need for articulated actions between professionals, services and public policies.

Although this review does not allow for direct clinical recommendations, the findings provide important insights to guide future practice and research. Among these contributions, the potential use of brief, self-administered instruments—such as the SCOFF and the EDE-Q—is noteworthy, particularly in high-demand, resource-limited primary care settings. The adoption of strategies that involve professional training, integration of digital tools, and well-defined referral pathways may enhance the early detection of ED.

A research agenda is therefore recommended, focusing on: (i) cross-cultural validation in diverse population groups; (ii) evaluation of the acceptability and applicability of the instruments across different clinical contexts; and (iii) development of training interventions for interdisciplinary teams. Emphasis is placed on the importance of incorporating eating disorder screening as a qualified and ongoing practice in primary care, grounded in validated instruments that are sensitive to local specificities and integrated with mental health care networks.

Future investigations that consider greater population diversity, both among users and professionals, are essential to consolidate screening as a qualified part of mental health care in primary care.

## Supporting information

S1 Appendix Preferred reporting items for systematic reviews and meta-analyses extension for scoping reviews (PRISMA-ScR) checklist. (PDF)

S2 Appendix Search strategies. Search strategies in the five databases, gray literature, and registries (October 2024).
(PDF)

S3 Appendix Excluded studies and reasons for exclusion (n = 108). (PDF)

S1 Table Characteristics of the empirical studies included in the review (n = 56). (PDF)

S2 Table Characteristics of the nonempirical studies included in the review (n = 21). (PDF)
