# Supplementary material for: Validated strategies for screening for eating disorders in primary health care: A scoping review with a focus on adolescents and adults
Source: PLoS One. 2026 Aug 3;21(8):e0347184. doi: 10.1371/journal.pone.0347184 (PMC13432121; doi:10.1371/journal.pone.0347184)
Supplement: S2 Appendix — Search strategies in the five databases, gray literature, and registries (October 2024). (PDF) [file pone.0347184.s002.pdf]

S2 Appendix. Search strategies. Search strategies in the five databases, gray literature, and registries (October 2024).

Databases, gray literature, and registries	Search strategy
**PUBMED (n=1033)**	(((((((((("Feeding and Eating Disorders"[Mesh]) OR ("Feeding and Eating Disorders"[Title/Abstract] OR "Eating and Feeding Disorders"[Title/Abstract] OR "Feeding Disorders"[Title/Abstract] OR "Feeding Disorder"[Title/Abstract] OR "Eating Disorders"[Title/Abstract] OR "Eating Disorder"[Title/Abstract] OR ("Anorexia Nervosa"[Mesh])) OR ("Anorexia Nervosa"[Title/Abstract])) OR ("Binge-Eating Disorder"[Mesh])) OR ("Binge Eating Disorder"[Title/Abstract] OR "Binge-Eating Disorders"[Title/Abstract])) OR (("Bulimia Nervosa"[Mesh]) AND ("Bulimia Nervosa"[Title/Abstract]))) OR ("Night Eating Syndrome"[Mesh])) OR ("Night Eating Syndromes"[Title/Abstract]) OR ("purging disorder") OR ("Other Specified Feeding and Eating Disorder"[Title/Abstract])) OR ("Eating Disorders Not Otherwise Specified"[Title/Abstract]))) AND (((("Primary Health Care"[Mesh]) OR ("Primary Healthcare"[Title/Abstract] OR "Primary Care"[Title/Abstract] OR "Community Health Centers"[Title/Abstract] OR "Community Health Centers"[Title/Abstract] OR "National Health Strategies"[Title/Abstract] OR "Basic Health Services"[Title/Abstract] OR "Unified Health System"[Title/Abstract])) OR ("Preventive Health Services"[Mesh])) OR ("Preventive Health Care"[Title/Abstract] OR "Preventive Health"[Title/Abstract] OR "Preventive Health Service"[Title/Abstract] OR "Preventive Health Programs"[Title/Abstract] OR "Preventive Health Program"[Title/Abstract] OR "Preventive Programs"[Title/Abstract] OR "Preventive Program"[Title/Abstract]))) AND (("identification" OR "instrument" OR "instruments" OR "questionnaire" OR "questionnaires" OR "screen" OR "screening" OR "measure" OR "measures"))
**CINAHL (n=154)**	("Feeding and Eating Disorders" OR "Eating and Feeding Disorders" OR "Feeding Disorders" OR "Feeding Disorder" OR "Eating Disorders" OR "Eating Disorder" OR "Anorexia Nervosa" OR "Binge-Eating Disorder" OR "Binge-Eating Disorders" OR "Bulimia Nervosa" OR "Night Eating Syndrome" OR "Night Eating Syndromes" OR "purging disorder" OR "Other Specified Feeding and Eating Disorder" OR "Eating Disorders Not Otherwise Specified") AND ("Primary Health Care" OR "Primary Healthcare" OR "Primary Care" OR "Community Health Centers" OR "Community Health Centers" OR "National Health Strategies" OR "Basic Health Services" OR "Unified Health System" OR "Preventive Health Services" OR "Preventive Health Care" OR "Preventive Health" OR "Preventive Health Service" OR "Preventive Health Programs" OR "Preventive Health Program "OR "Preventive Programs" OR "Preventive Program") AND ("identification" OR "instrument" OR "instruments" OR "questionnaire" OR "questionnaires" OR "screen" OR "screening" OR "measure" OR "measures")
**Embase (n=817)**	('feeding and eating disorders'/exp OR 'feeding and eating disorders' OR 'eating and feeding disorders' OR 'feeding disorders' OR 'feeding disorder'/exp OR 'feeding disorder' OR 'eating disorders'/exp OR 'eating disorders' OR 'eating disorder'/exp OR 'eating disorder' OR 'anorexia nervosa'/exp OR 'anorexia nervosa' OR 'binge-eating disorder'/exp OR 'binge-eating disorder' OR 'bulimia nervosa'/exp OR 'bulimia nervosa' OR 'night eating syndrome'/exp OR 'night eating syndrome' OR 'night eating syndromes' OR 'purging disorder'/exp OR 'purging disorder' OR 'other specified feeding and eating disorder' OR 'eating disorders not otherwise specified') AND ('primary health care'/de OR 'primary health care' OR 'primary healthcare'/de OR 'primary healthcare' OR 'primary care'/de OR 'primary care' OR 'community health centers'/de OR 'community health centers' OR 'national health strategies' OR 'basic health services' OR 'unified health system'/de OR 'unified health system' OR 'preventive health services'/de OR 'preventive health services' OR 'preventive health care'/de OR 'preventive health care' OR 'preventive health' OR 'preventive health service'/de OR 'preventive health service' OR 'preventive health programs' OR 'preventive health program'/de OR 'preventive health program' OR 'preventive programs' OR 'preventive program') AND ('identification'/de OR 'identification' OR

	'instrument'/de OR 'instrument' OR 'instruments'/de OR 'instruments' OR 'questionnaire'/de OR 'questionnaire' OR 'questionnaires'/de OR 'questionnaires' OR 'screen'/de OR 'screen' OR 'screening'/de OR 'screening' OR 'measure' OR 'measures')
**PsyINFO (n=324)**	((((IndexTermsFilt: ("Identification")) *OR* (IndexTermsFilt: ("Questionnaire")) *OR* (IndexTermsFilt: ("Screening")) *OR* (IndexTermsFilt: ("Screening Tests")))) *OR* (((Any Field: ("identification")) *OR* (Any Field: ("instrument")) *OR* (Any Field: ("instruments")) *OR* (Any Field: ("questionnaire")) *OR* (Any Field: ("questionnaires")) *OR* (Any Field: ("screen")) *OR* (Any Field: ("screening")) *OR* (Any Field: ("measure")) *OR* (Any Field: ("measures"))))) *AND* ((((IndexTermsFilt: ("Primary Health Care")) *OR* (IndexTermsFilt: ("Preventive Health Services")))) *OR* (((Any Field: ("Primary Healthcare")) *OR* (Any Field: ("Primary Care")) *OR* (Any Field: ("Community Health Centers")) *OR* (Any Field: ("Community Health Centers")) *OR* (Any Field: ("National Health Strategies")) *OR* (Any Field: ("Basic Health Services")) *OR* (Any Field: ("Unified Health System")) *OR* (Any Field: ("Preventive Health Care")) *OR* (Any Field: ("Preventive Health")) *OR* (Any Field: ("Preventive Health Service")) *OR* (Any Field: ("Preventive Health Programs")) *OR* (Any Field: ("Preventive Health Program ") *OR* Any Field: ("Preventive Programs")) *OR* (Any Field: ("Preventive Program"))))) *AND* ((((IndexTermsFilt: ("Feeding Disorders")) *OR* (IndexTermsFilt: ("Eating Disorders")) *OR* (IndexTermsFilt: ("Anorexia Nervosa")))) *OR* (((Any Field: ("Feeding and Eating Disorders")) *OR* (Any Field: ("Eating and Feeding Disorders")) *OR* (Any Field: ("Feeding Disorder")) *OR* (Any Field: ("Eating Disorder")) *OR* (Any Field: ("Binge-Eating Disorder")) *OR* (Any Field: ("Binge-Eating Disorders")) *OR* (Any Field: ("Bulimia Nervosa")) *OR* (Any Field: ("Night Eating Syndrome")) *OR* (Any Field: ("Night Eating Syndromes")) *OR* (Any Field: ("purging disorder")) *OR* (Any Field: ("Other Specified Feeding and Eating Disorder")) *OR* (Any Field: ("Eating Disorders Not Otherwise Specified")))))
**LILACS (n=230)**	(MH: "Feeding and Eating Disorders" OR (Transtornos da Alimentação e da Ingestão de Alimentos) OR (Trastornos de Alimentación y de la Ingestión de Alimentos) OR (Feeding and Eating Disorders) OR (Transtornos da Alimentação) OR (Transtornos da Ingestão de Alimentos) OR MH: F03.400$ OR MH: SP6.990$) AND (MH: "Primary Health Care" OR (Primary Health Care) OR (Atención Primaria de Salud) OR (Atenção Primária à Saúde) OR (Atendimento Básico) OR (Atendimento Primário) OR (Atendimento Primário de Saúde) OR (Atenção Básica) OR (Atenção Básica de Saúde) OR (Atenção Básica à Saúde) OR (Atenção Primária) OR (Atenção Primária de Saúde) OR (Atenção Primária em Saúde) OR (Cuidado Primário de Saúde) OR (Cuidado de Saúde Primário) OR (Cuidados Primários) OR (Cuidados Primários de Saúde) OR (Cuidados Primários à Saúde) OR (Cuidados de Saúde Primários) OR (Primeiro Nível de Assistência) OR (Primeiro Nível de Atendimento) OR (Primeiro Nível de Atenção) OR (Primeiro Nível de Atenção à Saúde) OR (Primeiro Nível de Cuidado) OR (Primeiro Nível de Cuidados) OR MH: N04.590.233.727$ OR SP2.630.121$) AND ((identification) OR (instrument) OR (instruments) OR (questionnaire) OR (questionnaires) OR (screen) OR (screening) OR (measure) OR (measures) OR (instrumento) OR (rastreio) OR (medidas) OR (triagem) OR (rastreo) OR (triage))
**Web of Science (n=419)**	TS=("Feeding and Eating Disorders" OR "Eating and Feeding Disorders" OR "Feeding Disorders" OR "Feeding Disorder" OR "Eating Disorders" OR "Eating Disorder" OR "Anorexia Nervosa" OR "Binge-Eating Disorder" OR "Bulimia Nervosa" OR "Night Eating Syndrome" OR "Night Eating Syndromes" OR "purging disorder" OR "Other Specified Feeding and Eating Disorder" OR "Eating Disorders Not Otherwise Specified") AND TS=("Primary Health Care" OR "Primary Healthcare" OR "Primary Care" OR "Community Health Centers" OR "Community Health Centers" OR "National Health Strategies" OR "Basic Health Services" OR "Unified Health System" OR "Preventive Health Services" OR "Preventive Health

	Care" OR "Preventive Health" OR "Preventive Health Service" OR "Preventive Health Programs" OR "Preventive Health Program" OR "Preventive Programs" OR "Preventive Program") AND TS=("identification" OR "instrument" OR "instruments" OR "questionnaire" OR "questionnaires" OR "screen" OR "screening" OR "measure" OR "measures")
**Google Scholar (291 results)**	"eating disorders" AND "primary healthcare" AND screen
**ProQuest (52 results)**	(((("Feeding and Eating Disorders" OR "Eating and Feeding Disorders" OR "Feeding Disorders" OR "Feeding Disorder" OR "Eating Disorders" OR "Eating Disorder" OR "Anorexia Nervosa" OR "Binge-Eating Disorder" OR "Binge-Eating Disorders" OR "Bulimia Nervosa" OR "Night Eating Syndrome" OR "Night Eating Syndromes" OR "purging disorder" OR "Other Specified Feeding and Eating Disorder" OR "Eating Disorders Not Otherwise Specified") AND ("Primary Health Care" OR "Primary Healthcare" OR "Primary Care" OR "Community Health Centers" OR "Community Health Centers" OR "National Health Strategies" OR "Basic Health Services" OR "Unified Health System" OR "Preventive Health Services" OR "Preventive Health Care" OR "Preventive Health" OR "Preventive Health Service" OR "Preventive Health Programs" OR "Preventive Health Program "OR "Preventive Programs" OR "Preventive Program") AND ("identification" OR "instrument" OR "instruments" OR "questionnaire" OR "questionnaires" OR "screen" OR "screening" OR "measure" OR "measures"))))
