# Supplementary material for: Validated strategies for screening for eating disorders in primary health care: A scoping review with a focus on adolescents and adults
Source: PLoS One. 2026 Aug 3;21(8):e0347184. doi: 10.1371/journal.pone.0347184 (PMC13432121; doi:10.1371/journal.pone.0347184)
Supplement: S3 Appendix — (PDF) [file pone.0347184.s003.pdf]

S3 Appendix. Excluded studies and reasons for exclusion (n=108).

	Author/Year	Razão para exclusão
(1)	Al Mestaka et al., 2023	4
(2)	Allen et al., 2011	3
(3)	Andersen, 1999	1
(4)	Anstine, Grinenko, 2000	3
(5)	Ayerbe-García et al., 2003	1
(6)	Barnes et al., 2011	4
(7)	Belsky et al., 2023	1
(8)	Bjørnelv, 2004	1
(9)	Blesa, 2003	2
(10)	Brown et al., 2016	1
(11)	Bryant et al., 2021	3
(12)	Bryant et al., 2024	3
(13)	Bursten et al., 1996	2
(14)	Burton et al., 2016	2
(15)	Bye, 2020	3
(16)	Cardona, 2016	3
(17)	Chacko et al., 2015	2
(18)	Chamay-Weber et al., 2013	1
(19)	Chevinsky, Wadden, Chao, 2020	3
(20)	Coope et al., 2024	2
(21)	Cousins et al., 2015	2
(22)	Currin, Waller, Schmidt, 2009	2
(23)	d'Emden et al., 2015	1
(24)	Davidson et al., 2022	1
(25)	de la Torre et al., 2006	2
(26)	DerMarderosian, Hall, 2011	2
(27)	Doherty, McNamee, 2015	1

(28)	Dörsam et al., 2022	3
(29)	Duarte, Pinto-Gouveia, Ferreira, 2015	3
(30)	Garcia et al., 2009	1
(31)	Duffin, 2006	1
(32)	Endacott et al., 2006	2
(33)	Fang, 2021	1
(34)	Feigel-Guiller, 2017	1
(35)	Feltner et al., 2022	1
(36)	Franklin, 2020	2
(37)	Freund et al., 1993	2
(38)	Garcia et al., 2010	3
(39)	Gerges, Obeid, Hallit, 2022	3
(40)	Gil Canalda et al., 2003	2
(41)	Goebel-Fabbri et al., 2009	3
(42)	Goode et al., 2023	2
(43)	Goodyear-Smith et al., 2008	2
(44)	Gowey, 2015	3
(45)	Green et al., 2008	2
(46)	Guarda, Redgrave, 2004	2
(47)	Hague, 2010	1
(48)	Hamm, 1993	1
(49)	Hay et al., 2020	3
(50)	Herman et al., 2016	3
(51)	House et al., 2022	3
(52)	House, Loud, Shubkin, 2013	2
(53)	Ivancic et al., 2021	2

(54)	Jacobi, Abascal, Taylor, 2004	2
(55)	Jasik, 2014	2
(56)	Jin, 2022	1
(57)	Johnson et al., 2002	2
(58)	Johnson, 1982	2
(59)	Julián Viñals et al., 2007	1
(60)	Kagan, Melrose, 2003	1
(61)	Kenkre, 2013	1
(62)	King et al., 2023	2
(63)	Kondo, Sokol, 2006	2
(64)	Kons, Essayli, Shook, 2024	2
(65)	Kuttler, 2010	1
(66)	Lange, 2016	4
(67)	Larrañaga, Garcia-Soidan, Garcia-Mayor, 2013	1
(68)	Leti et al., 2020	3
(69)	Levine, 2024	3
(70)	Liengme, Michaud, 2022	2
(71)	Linsenmeyer et al., 2024	3
(72)	Luck et al., 2003	1
(73)	Markowitz et al., 2010	3
(74)	Mauhourat, Gicquel, Mignot, 2022	2
(75)	McBride, 2011	1
(76)	Morgan, Reid, Lacey, 1999	3
(77)	Nagata, Golden, 2022	1

(78)	Nicolau et al., 2015	2
(79)	Nunes et al., 2012	2
(80)	Parker, Lyons, Bonner, 2005	3
(81)	Parmar et al., 2021	1
(82)	Peat, Feltner, 2022	1
(83)	Petito, Iuso, Bellomo, 2013	1
(84)	Powers, Santana, 2002	2
(85)	Prnjak et al., 2020	3
(86)	Pursey et al., 2020	3
(87)	Raphael, 1996	1
(88)	Read, McComiskey, 2021	4
(89)	Resch, 2003	2
(90)	Robinson, Boachie, Lafrance, 2013	2
(91)	Rodríguez-Santos et al., 2012	1
(92)	Russon et al., 2019	2
(93)	Rynkiewicz et al., 2022	1
(94)	Seferovic et al., 2019	1
(95)	Segura-García et al., 2015	3
(96)	Silva et al., 2012	3
(97)	Slimane Zrafi et al., 2022	4
(98)	Smith, Harrop, 2024	3
(99)	Spitzer, Kroenke, Williams, 1999	2
(100)	Stevens, 1999	1
(101)	Stidham et al., 2010	1

(102)	Swarna Nantha et al., 2016	2
(103)	Swarna Nantha et al., 2020	2
(104)	Sweeney, 2016	1
(105)	Uehara, Oshima, 2014	1
(106)	Vander Wal et al., 2005	3
(107)	Walsh, Wheat, Freund, 2000	2
(108)	Zuijdwijk et al., 2014	1

1. Articles in the form of letters, book chapters, conference abstracts, opinion pieces, clinical guidelines, study protocols, recommendations, commentaries, and news items.

2. Articles that did not mention validated screening strategies for eating disorders (ED) or the feasibility of their implementation.

3. Articles not conducted in the context of primary health care or in generalizable settings.

4. Articles that did not include adolescents and adults or primary health care professionals in the sample, as well as those that included individuals already diagnosed with ED.

References

1. Al Mestaka, N., Alneyadi, A., AlAhbabi, A., AlMatrushi, A., AlSaadi, R., & Alketbi, L. B. (2023). Prevalence of probable eating disorders and associated risk factors in children and adolescents aged 5-16 years in Al Ain City, United Arab Emirates: observational case-control study. *Journal of eating disorders*, *11*(1), 114. https://doi.org/10.1186/s40337-023-00840-w
2. Allen, K. L., Fursland, A., Watson, H., & Byrne, S. M. (2011). Eating disorder diagnoses in general practice settings: comparison with structured clinical interview and self-report questionnaires. *Journal of mental health (Abingdon, England)*, *20*(3), 270–280. https://doi.org/10.3109/09638237.2011.562259
3. Andersen, A. E. (1999). The diagnosis and treatment of eating disorders in primary care medicine. In P. S. Mehler & A. E. Andersen (Eds.), *Eating disorders: A guide to medical care and complications* (pp. 1–26). Johns Hopkins University Press.
4. Anstine, D., & Grinenko, D. (2000). Rapid screening for disordered eating in college-aged females in the primary care setting. *The Journal of adolescent health : official publication of the Society for Adolescent Medicine*, *26*(5), 338–342. https://doi.org/10.1016/s1054-139x(99)00120-2
5. Ayerbe-García, M. L., González-López, L., López-Larrayoz, E., Ezquerra-Gadea, J. El test SCOFF: una posible herramienta de cribado de los trastornos de comportamiento alimentario en atención primaria, *Atención Primaria,* Volume 32, Issue 5, 2003, Pages 315-316, doi.org/10.1016/S0212-6567(03)74544-2.

6. Barnes, R. D., Masheb, R. M., White, M. A., & Grilo, C. M. (2011). Comparison of methods for identifying and assessing obese patients with binge eating disorder in primary care settings. *The International journal of eating disorders*, *44*(2), 157–163. https://doi.org/10.1002/eat.20802

7. Belsky, Natasha Ann et al. 53. Improving Standardized Eating Disorder Screening in an Adolescent Primary Care Setting. *Journal of Adolescent Health*, Volume 72, Issue 3, S34

8. Bjørnelv, S. (2016). Spiseforstyrrelser i allmennpraksis. *Tidsskr Nor Lægeforen* nr. 18, 2004; 124: 2372–5

9. Galdón Blesa, M. P., López-Torres, J. D., David García, Y., Gómez Honrubia, M. C., Escobar Rabadán, F., & Martínez Ramírez, M. (2003). Detección de bulimia nerviosa en las consultas de Atención Primaria [Detection of bulimia nervosa in primary health care consultations]. *Actas espanolas de psiquiatria*, *31*(3), 129–132.

10. Brown, Amanda Joelle; Schebendach, Janet; Walsh, B. Timothy. Handbook of assessment and treatment of eating disorders. 2016;():23-43. Arlington, VA, US American Psychiatric Publishing, Inc. 2016. Ref ID: 2015-55278-002

11. Bryant, E., Miskovic-Wheatley, J., Touyz, S. W., Crosby, R. D., Koreshe, E., & Maguire, S. (2021). Identification of high risk and early stage eating disorders: first validation of a digital screening tool. *Journal of eating disorders*, *9*(1), 109. https://doi.org/10.1186/s40337-021-00464-y

12. Bryant, E., Spielman, K., Burton, A. L., Ong, S. H., Livney, J., Corry, S., & Maguire, S. (2024). Identifying eating disorders at the earliest opportunity: Testing the reliability of an online eating disorder screener (IOI-S) in primary care and youth mental health settings. *Early intervention in psychiatry*, *18*(6), 446–454. https://doi.org/10.1111/eip.13486

13. Bursten, M. S., Gabel, L. L., Brose, J. A., & Monk, J. S. (1996). Detecting and treating bulimia nervosa: how involved are family physicians?. *The Journal of the American Board of Family Practice*, *9*(4), 241–248.

14. Burton, A. L., Abbott, M. J., Modini, M., & Touyz, S. (2016). Psychometric evaluation of self-report measures of binge-eating symptoms and related psychopathology: A systematic review of the literature. *The International journal of eating disorders*, *49*(2), 123–140. https://doi.org/10.1002/eat.22453

15. Bye, A. (Author). Identification and management of eating disorders in pregnancy: a multi-method study of maternity and health visiting services. 29 Jun 2020. University College London. Student thesis: Doctoral Thesis › Doctor of Philosophy

16. Cardona, Genevieve Rae. Initial testing of the Risk Assessment of Eating Disorders (RAED) tool for use in primary care of Hispanic women. 2016;(10149998):92 United States -- Arizona The University of Arizona 2016

17. Chacko, S. A., Chiodi, S. N., & Wee, C. C. (2015). Recognizing disordered eating in primary care patients with obesity. *Preventive medicine*, *72*, 89–94. https://doi.org/10.1016/j.ypmed.2014.12.024

18. Chamay-Weber, C., Kruseman, M., Di Capua, D., Suringar, V., Farpour-Lambert, N., Lanza, L., Narring, F., & Haller, D. M. (2013). Screening obese adolescents for binge eating disorder: The ADO-BEDS auto-questionnaire. *Swiss Medical Weekly, 143*, 17S.

19. Chevinsky, J. D., Wadden, T. A., & Chao, A. M. (2020). Binge Eating Disorder in Patients with Type 2 Diabetes: Diagnostic and Management Challenges. *Diabetes, metabolic syndrome and obesity : targets and therapy*, *13*, 1117–1131. https://doi.org/10.2147/DMSO.S213379

20. Coop, A., Clark, A., Morgan, J., Reid, F., & Lacey, J. H. (2024). The use and misuse of the SCOFF screening measure over two decades: a systematic literature review. *Eating and weight disorders : EWD*, *29*(1), 29. https://doi.org/10.1007/s40519-024-01656-6

21. Cousins, A., Freizinger, M., Duffy, M. E., Gregas, M., & Wolfe, B. E. (2015). Self-report of eating disorder symptoms among women with and without infertility. *Journal of obstetric, gynecologic, and neonatal nursing : JOGNN*, *44*(3), 380–388. https://doi.org/10.1111/1552-6909.12573

22. Currin, L., Waller, G., & Schmidt, U. (2009). Primary care physicians' knowledge of and attitudes toward the eating disorders: do they affect clinical actions?. *The International journal of eating disorders*, *42*(5), 453–458. https://doi.org/10.1002/eat.20636

23. d'Emden, H., McDermott, B., Gibbons, K., Harris, M., & Cotterill, A. (2015). Choosing a screening tool to assess disordered eating in adolescents with type 1 diabetes mellitus. *Journal of diabetes and its complications*, *29*(1), 2–4. https://doi.org/10.1016/j.jdiacomp.2014.09.008

24. US Preventive Services Task Force, Davidson, K. W., Barry, M. J., Mangione, C. M., Cabana, M., Chelmow, D., Coker, T. R., Davis, E. M., Donahue, K. E., Jaén, C. R., Kubik, M., Li, L., Ogedegbe, G., Pbert, L., Ruiz, J. M., Silverstein, M., Stevermer, J., & Wong, J. B. (2022). Screening for Eating Disorders in Adolescents and Adults: US Preventive Services Task Force Recommendation Statement. *JAMA*, *327*(11), 1061–1067. https://doi.org/10.1001/jama.2022.1806

25. Miján de la Torre, A., Pérez-García, A., Martín de la Torre, E., & de Mateo Silleras, B. (2006). Is an integral nutritional approach to eating disorders feasible in primary care?. *The British journal of nutrition*, *96 Suppl 1*, S82–S85. https://doi.org/10.1079/bjn20061706

26. DerMarderosian, D., & Hall, A. (2011). Early identification of eating disorders in primary care pediatrics. *Medicine & Health, Rhode Island*, *94*(7), 197–199.

27. Doherty, S., & McNamee, L. (2015). General practitioner knowledge, skills, and attitudes to eating disorders. *BMC Proceedings*, 9(Suppl 1), A33. https://doi.org/10.1186/1753-6561-9-S1-A33

28. Dörsam, A. F., Bye, A., Graf, J., Howard, L. M., Throm, J. K., Müller, M., Wallwiener, S., Zipfel, S., Micali, N., & Giel, K. E. (2022). Screening instruments for eating disorders in pregnancy: Current evidence, challenges, and future directions. *The International journal of eating disorders*, *55*(9), 1208–1218. https://doi.org/10.1002/eat.23780

29. Duarte, C., Pinto-Gouveia, J., & Ferreira, C. (2015). Expanding binge eating assessment: Validity and screening value of the Binge Eating Scale in women from the general population. *Eating behaviors*, *18*, 41–47. https://doi.org/10.1016/j.eatbeh.2015.03.007

30. Garcia, F. D., Grigioni, S., Houy-Durand, E., Allais, E., Merygnac, G., Thibaut, F., & Déchelotte, P. (2009). Translation of SCOFF questionnaire and validation of this French version for the screening of eating disorders in a student population. *European Psychiatry*, 24(Suppl 1), S745. https://doi.org/10.1016/S0924-9338(09)70758-X

31. Duffin, C. (2006). Call for better identification of eating disorders in primary care. *Nursing Standard*: News, 21(14–16), 10.

32. Endacott, R., Kidd, S., Deacon-Crouch, M., Judd, F., Menzel, M., & Cornett, M. (2006). Developing new services for eating disorders: an evaluation study. *Australasian psychiatry : bulletin of Royal Australian and New Zealand College of Psychiatrists*, *14*(1), 57–62. https://doi.org/10.1080/j.1440-1665.2006.02246.x

33. Fang, C. (2021). Improving binge eating disorder screening in primary care settings (Master's thesis, Boston University School of Medicine). Boston University.

34. Feigel-Guiller, B., Baron, S., Rocher, B., & Krempf, M. (2017). Dépistage des troubles du comportement alimentaire chez l'adolescent diabétique de type 1 [Screening for disturbed eating behavior in adolescents with type 1 diabetes]. *Archives de pediatrie : organe officiel de la Societe francaise de pediatrie*, *24*(7), 695–696. https://doi.org/10.1016/j.arcped.2017.04.008

35. Feltner, C., Peat, C., Reddy, S., Riley, S., Berkman, N., Middleton, J. C., Balio, C., Coker-Schwimmer, M., & Jonas, D. E. (2022). Screening for eating disorders in adolescents and adults: An evidence review for the U.S. Preventive Services Task Force. Rockville, MD: Agency for Healthcare Research and Quality (US).

36. Franklin, D. R. (2020). A clinical training on eating disorders for community healthcare providers and stakeholders (Doctoral dissertation, Capella University). ProQuest LLC.

37. Freund, K. M., Graham, S. M., Lesky, L. G., & Moskowitz, M. A. (1993). Detection of bulimia in a primary care setting. *Journal of general internal medicine*, *8*(5), 236–242. https://doi.org/10.1007/BF02600088

38. Garcia, F. D., Grigioni, S., Chelali, S., Meyrignac, G., Thibaut, F., & Dechelotte, P. (2010). Validation of the French version of SCOFF questionnaire for screening of eating disorders among adults. *The world journal of biological psychiatry : the official journal of the World Federation of Societies of Biological Psychiatry*, *11*(7), 888–893. https://doi.org/10.3109/15622975.2010.483251

39. Gerges, S., Obeid, S., & Hallit, S. (2022). Initial psychometric properties of an Arabic version of the disordered eating attitudes in pregnancy scale (A-DEAPS) among Lebanese pregnant women. *Journal of Eating Disorders, 10,* 175. https://doi.org/10.1186/s40337-022-00710-x

40. Gil Canalda, M. I., Candela Villanueva, J. P., & Cecilia Rodríguez, M. (2003). Atención primaria y trastornos de la alimentación: nuestra actitud frente a ellos (I) [Primary care and eating disorders: our attitude towards them (I)]. *Atencion primaria*, *31*(3), 178–180. https://doi.org/10.1016/s0212-6567(03)70679-9

41. Goebel-Fabbri A. E. (2009). Disturbed eating behaviors and eating disorders in type 1 diabetes: clinical significance and treatment recommendations. *Current diabetes reports*, *9*(2), 133–139. https://doi.org/10.1007/s11892-009-0023-8

42. Goode, R. W., Bardone-Cone, A., Wilhoit-Reeves, S., Williams, L., Malian, H., Coan, D., Noem, T., & Tate, D. F. (2023). Creating an appetite awareness and lifestyle modification intervention for Black women at risk for binge eating disorder: A pilot open trial. *Clinical obesity*, *13*(6), e12613. https://doi.org/10.1111/cob.12613

43. Goodyear-Smith, F., Coupe, N. M., Arroll, B., Elley, C. R., Sullivan, S., & McGill, A. T. (2008). Case finding of lifestyle and mental health disorders in primary care: validation of the 'CHAT' tool. *The British journal of general practice : the journal of the Royal College of General Practitioners*, *58*(546), 26–31. https://doi.org/10.3399/bjgp08X263785

44. Gowey, M. A. (2015). Executive function and binge eating in overweight and obese youth (Doctoral dissertation, University of Florida). University of Florida.

45. Green, H., Johnston, O., Cabrini, S., Fornai, G., & Kendrick, T. (2008). General practitioner attitudes towards referral of eating-disordered patients: a vignette study based on the theory of planned behaviour. *Mental health in family medicine*, *5*(4), 213–218.

46. Guarda, A. S., & Redgrave, G. W. (2004). Eating disorders: Detection, assessment, and treatment in primary care. *Behavioral Medicine*, 4(9), October.

47. Hague A. L. (2010). Eating disorders: screening in the dental office. *Journal of the American Dental Association (1939)*, *141*(6), 675–678. https://doi.org/10.14219/jada.archive.2010.0257

48. Hamm R. M. (1993). Two-question screen for bulimia. *Journal of general internal medicine*, *8*(12), 715–716. https://doi.org/10.1007/BF02598301

49. Hay, P., Ghabrial, B., Mannan, H., Conti, J., Gonzalez-Chica, D., Stocks, N., Heriseanu, A., & Touyz, S. (2020). General practitioner and mental healthcare use in a community sample of people with diagnostic threshold symptoms of bulimia nervosa, binge-eating disorder, and other eating disorders. *The International journal of eating disorders*, *53*(1), 61–68. https://doi.org/10.1002/eat.23174

50. Herman, B. K., Deal, L. S., DiBenedetti, D. B., Nelson, L., Fehnel, S. E., & Brown, T. M. (2016). Development of the 7-Item Binge-Eating Disorder Screener (BEDS-7). *The primary care companion for CNS disorders*, *18*(2), 10.4088/PCC.15m01896. https://doi.org/10.4088/PCC.15m01896

51. House, E. T., Lister, N. B., Seidler, A. L., Li, H., Ong, W. Y., McMaster, C. M., Paxton, S. J., & Jebeile, H. (2022). Identifying eating disorders in adolescents and adults with overweight or obesity: A systematic review of screening questionnaires. *The International journal of eating disorders*, *55*(9), 1171–1193. https://doi.org/10.1002/eat.23769

52. House, S., Loud, K., & Shubkin, C. (2013). Female athlete triad for the primary care pediatrician. *Current opinion in pediatrics*, *25*(6), 755–761. https://doi.org/10.1097/MOP.0000000000000033

53. Ivancic, L., Maguire, S., Miskovic-Wheatley, J., Harrison, C., & Nassar, N. (2021). Prevalence and management of people with eating disorders presenting to primary care: A national study. *The*

Australian and New Zealand journal of psychiatry, *55*(11), 1089–1100. https://doi.org/10.1177/0004867421998752

54. Jacobi, C., Abascal, L., & Taylor, C. B. (2004). Screening for eating disorders and high-risk behavior: caution. *The International journal of eating disorders*, *36*(3), 280–295. https://doi.org/10.1002/eat.20048
55. Jasik C. B. (2014). Body image and health: eating disorders and obesity. *Primary care*, *41*(3), 519–537. https://doi.org/10.1016/j.pop.2014.05.003
56. Jin J. Screening for Eating Disorders in Adolescents and Adults. *JAMA.* 2022;327(11):1100. doi:10.1001/jama.2022.2428
57. Johnson, J. G., Harris, E. S., Spitzer, R. L., & Williams, J. B. (2002). The patient health questionnaire for adolescents: validation of an instrument for the assessment of mental disorders among adolescent primary care patients. *The Journal of adolescent health : official publication of the Society for Adolescent Medicine*, *30*(3), 196–204. https://doi.org/10.1016/s1054-139x(01)00333-04
58. Johnson, M. (1982). Anorexia nervosa: Framework for early identification and intervention. *Issues in Mental Health Nursing, 4*(2), 87–99. https://doi.org/10.3109/01612848209141046
59. Julián Viñals, R., Ramírez Arrizabalaga, R., Melgar Borrego, A. B., & Gómez Lumbreras, A. (2007). Detección precoz, en atención primaria, de trastornos de la conducta alimentaria en los adolescentes [Early detection in primary care of food conduct disorder in adolescents]. *Atencion primaria*, *39*(6), 330–331. https://doi.org/10.1157/1310629
60. Kagan, S., & Melrose, C. (2003). The SCOFF questionnaire was less sensitive but more specific than the ESP for detecting eating disorders. *Evidence-based nursing*, *6*(4), 118. https://doi.org/10.1136/ebn.6.4.118
61. Kenkre, J. (2013). Review: The effectiveness of a brief eating disorder training programme in medical settings. *Journal of Research in Nursing*, 18 (6), 559–560. https://doi.org/10.1177/1744987112452209
62. King, P. R., Buchholz, L. J., Tauriello, S., & Wray, L. O. (2023). Qualitative exploration of factors influencing women veterans' disordered eating symptoms and treatment preferences in VHA primary care. *Families, systems & health : the journal of collaborative family healthcare*, *41*(2), 214–221. https://doi.org/10.1037/fsh0000740
63. Kondo, D. G., & Sokol, M. S. (2006). Eating disorders in primary care. A guide to identification and treatment. *Postgraduate medicine*, *119*(3), 59–65. https://doi.org/10.1080/00325481.2006.11446052
64. Kons, K., Essayli, J., & Shook, J. (2024). Comparing the knowledge, attitudes, and practices of pediatric and family medicine clinicians toward atypical anorexia nervosa versus anorexia nervosa. *The International journal of eating disorders*, *57*(4), 993–1001. https://doi.org/10.1002/eat.24095
65. Kuttler, M. E. (2010). Eating disorders. *The Journal of the American Dental Association,* 141(9), 1054.
66. Lange, N. J. (2016). Eating disorder screening, evaluation, and referral in the primary care setting (Doctoral dissertation, North Dakota State University). North Dakota State University Graduate School.
67. Larrañaga, A., Garcia-Soidan, J., & Garcia-Mayor, R. V. (2013). Eating disorders and food anomalous behaviour in type 2 diabetes adults. *Diabetologia,* 56, S460. https://doi.org/10.1007/s00125-013-3012-
68. Leti, M. M., Pop, A. L., Garner, D. M., & Dobrescu, I. (2020). Eating disorders in children and adolescents: An updated review on screening methods. Preprints.
69. Levine M. P. (2024). Prevention of eating disorders: 2023 in review. *Eating disorders*, *32*(3), 223–246. https://doi.org/10.1080/10640266.2024.2345995
70. Liengme, N., & Michaud, P.-A. (2002). Psychosocial screening in adolescence: A challenge and a responsibility for primary care physicians. *Médecine et Hygiène*, 60(2413), 2102–2109.
71. Linsenmeyer, W., Stiles, D., Garwood, S., Giedinghagen, A., Lewis, C., & Strand, G. (2024). The Sick, Control, One Stone, Fat, Food (SCOFF) is a Valid Eating Disorder Questionnaire to Use With Transgender Youth. *Clinical pediatrics*, *63*(7), 971–976. https://doi.org/10.1177/00099228231200754

72. Luck, A. J., Morgan, J. F., Reid, F., & Wilson, S. A. (2003). A simple 5 item questionnaire accurately detected eating disorders in women in primary care. *Evidence-Based Medicine*, 8(3), 90. https://doi.org/10.1136/ebm.8.3.90

73. Markowitz, J. T., Butler, D. A., Volkening, L. K., Antisdel, J. E., Anderson, B. J., & Laffel, L. M. (2010). Brief screening tool for disordered eating in diabetes: internal consistency and external validity in a contemporary sample of pediatric patients with type 1 diabetes. *Diabetes care*, *33*(3), 495–500. https://doi.org/10.2337/dc09-1890

74. Mauhourat, J., Gicquel, L., & Mignot, S. (2022). Anorexiclic: A diagnosis and management support tool of patients who are suffering from eating disorders for general practitioners. *EXERCER* - La Revue Francophone de Médecine Générale, (185), 312–316.

75. McBride, D. L. (2011). New screening guidelines for eating disorders. *Journal of Pediatric Nursing, 26,* 377–378.

76. Morgan, J. F., Reid, F., & Lacey, J. H. (1999). The SCOFF questionnaire: assessment of a new screening tool for eating disorders. *BMJ (Clinical research ed.)*, *319*(7223), 1467–1468. https://doi.org/10.1136/bmj.319.7223.1467

77. Nagata, J. M., & Golden, N. H. (2022). New US Preventive Services Task Force Recommendations on Screening for Eating Disorders. *JAMA internal medicine*, *182*(5), 471–473. https://doi.org/10.1001/jamainternmed.2022.0121

78. Nicolau, J., Simó, R., Sanchís, P., Ayala, L., Fortuny, R., Zubillaga, I., & Masmiquel, L. (2015). Eating disorders are frequent among type 2 diabetic patients and are associated with worse metabolic and psychological outcomes: results from a cross-sectional study in primary and secondary care settings. *Acta diabetologica*, *52*(6), 1037–1044. https://doi.org/10.1007/s00592-015-0742-z

79. Nunes, M. A., Pinheiro, A. P., Camey, S. A., & Schmidt, M. I. (2012). Binge eating during pregnancy and birth outcomes: a cohort study in a disadvantaged population in Brazil. *The International journal of eating disorders*, *45*(7), 827–831. https://doi.org/10.1002/eat.2202

80. Parker, S. C., Lyons, J., & Bonner, J. (2005). Eating disorders in graduate students: exploring the SCOFF questionnaire as a simple screening tool. *Journal of American college health : J of ACH*, *54*(2), 103–107. https://doi.org/10.3200/JACH.54.2.103-107

81. Parmar, D. D., Alabaster, A., Vance, S., Jr, Ritterman Weintraub, M. L., & Lau, J. S. (2021). Disordered Eating, Body Image Dissatisfaction, and Associated Healthcare Utilization Patterns for Sexual Minority Youth. *The Journal of adolescent health : official publication of the Society for Adolescent Medicine*, *69*(3), 470–476. https://doi.org/10.1016/j.jadohealth.2021.02.002

82. Peat, C. M., & Feltner, C. (2022). Addressing eating disorders in primary care: Understanding screening recommendations and opportunities to improve care. *The International journal of eating disorders*, *55*(9), 1202–1207. https://doi.org/10.1002/eat.23786

83. Petito, A., Iuso, S., & Bellomo, A. (2013). Screening for eating disorders by primary care in students of degree of physical education and sports. *Psychotherapy and Psychosomatics, 82*(Suppl. 1), 84–85. https://doi.org/10.1159/000354142

84. Powers, P. S., & Santana, C. A. (2002). Eating disorders: a guide for the primary care physician. *Primary care*, *29*(1), 81–vii. https://doi.org/10.1016/s0095-4543(03)00075-7

85. Prnjak, K., Mitchison, D., Griffiths, S., Mond, J., Gideon, N., Serpell, L., & Hay, P. (2020). Further development of the 12-item EDE-QS: identifying a cut-off for screening purposes. *BMC psychiatry*, *20*(1), 146. https://doi.org/10.1186/s12888-020-02565-5

86. Pursey, K. M., Hart, M., Jenkins, L., McEvoy, M., & Smart, C. E. (2020). Screening and identification of disordered eating in people with type 1 diabetes: A systematic review. *Journal of diabetes and its complications*, *34*(4), 107522. https://doi.org/10.1016/j.jdiacomp.2020.107522

87. Raphael F. The prevention of eating disorders. In: Kendrick T, Tylee A, Freeling P, eds. *The Prevention of Mental Illness in Primary Care*. Cambridge University Press; 1996:207-22 1

88. Read, A. K., & McComiskey, C. A. (2021). The implementation and evaluation of the SCOFF (Sick, Control, One Stone, Fat, Food) eating disorder screening tool for children and adolescents. *Pediatric Nursing, 47*(3), 124–132.

89. Resch, M. (2003). [Employment and effectiveness of the BITE questionnaire in screening for eating disorders]. *Orvosi Hetilap*, 144(46), 2277–2281.

90. Robinson, A. L., Boachie, A., & Lafrance, G. A. (2013). "I Want Help!": Psychologists' and physicians' competence, barriers, and needs in the management of eating disorders in children and adolescents in Canada. *Canadian Psychology* / Psychologie canadienne, 54*(3), 160–166.

91. Rodríguez-Santos, L., Leal, F. J. V., Fuentes, M. I. R., Herráiz, M. A. G., Mateos, I. F., & Gutiérrez, M. A. (2012). Detection, assessment, and monitoring of eating disorders in primary care settings. *Mental Health and Family Medicine Working Together*, 11–17. Medimond

92. Russon, J., Mensinger, J., Herres, J., Shearer, A., Vaughan, K., Wang, S. B., & Diamond, G. S. (2019). Identifying Risk Factors for Disordered Eating among Female Youth in Primary Care. *Child psychiatry and human development*, *50*(5), 727–737. https://doi.org/10.1007/s10578-019-00875-8

93. Rynkiewicz, A., Dembiński, Ł., Koletzko, B., Michaud, P. A., Hadjipanayis, A., Grossman, Z., Korslund, K., King, B. H., Treasure, J., Peregud-Pogorzelski, J., Del Torso, S., Valiulis, A., & Mazur, A. (2022). Adolescents With Eating Disorders in Pediatric Practice - The European Academy of Paediatrics Recommendations. *Frontiers in pediatrics*, *10*, 806399. https://doi.org/10.3389/fped.2022.806399

94. Seferovic, A., Dianes, G. N., Juan, B., Larsen, D., Oyler, V., & Ragoza, Y. (2019). What is the best screening tool for eating disorders in the primary care setting? *Evidence-Based Practice,* 22(3), 12. https://doi.org/10.1097/EBP.0000000000000182

95. Segura-García, C., Aloi, M., Rania, M., Ciambrone, P., Palmieri, A., Pugliese, V., Ruiz Moruno, A. J., & De Fazio, P. (2015). Ability of EDI-2 and EDI-3 to correctly identify patients and subjects at risk for eating disorders. *Eating behaviors*, *19*, 20–23. https://doi.org/10.1016/j.eatbeh.2015.06.010

96. Silva, J. R., Behar, R., Cordella, P., Ortiz, M., Jaramillo, K., Alvarado, R., & Jorquera, M. J. (2012). Estudio multicéntrico para la validación de la versión en español del Eating Disorder Diagnostic Scale [Validation of the Spanish version of the Eating Disorders Diagnostic Scale]. *Revista medica de Chile*, *140*(12), 1562–1570. https://doi.org/10.4067/S0034-98872012001200007

97. Slimane Zrafi, B., Omezzine Gniwa, R., Abdelkafi Koubaa, A., Bouali, W., & Sriha Belguith, A. (2022). Eating disorders among women in primary care (Monastir, Tunisia). *La Tunisie medicale*, *100*(3), 209–216.

98. Smith, E. K., & Harrop, E. N. (2024). "That's not at all what I needed" trans adults' perspectives on trans-affirming primary care and eating disorders. *Social science & medicine (1982)*, *348*, 116836. https://doi.org/10.1016/j.socscimed.2024.116836

99. Spitzer, R. L., Kroenke, K., & Williams, J. B. (1999). Validation and utility of a self-report version of PRIME-MD: the PHQ primary care study. Primary Care Evaluation of Mental Disorders. Patient Health Questionnaire. *JAMA*, *282*(18), 1737–1744. https://doi.org/10.1001/jama.282.18.1737

100. Stevens, S., Savageau, J., Luckmann, R., & Sattler, A. (1999). Screening for eating disorders: A survey of primary care physicians. *Journal of Adolescent Health*, 24(2), 96.

101. Stidham Hall, K., O'Connell White, K., Rickert, V., Reame, N., & Westhoff, C. L. (2010). Do depressed mood, psychological stress, and eating disordered symptoms increase the risk for oral contraceptive discontinuation in young minority women? *Contraception,* 82 (2), 186. https://doi.org/10.1016/j.contraception.2010.04.035

102. Swarna Nantha, Y., Abd Patah, N. A., & Ponnusamy Pillai, M. (2016). Preliminary validation of the Malay Yale Food Addiction Scale: Factor structure and item analysis in an obese population. *Clinical nutrition ESPEN*, *16*, 42–47. https://doi.org/10.1016/j.clnesp.2016.08.001

103. Swarna Nantha, Y., Kalasivan, A., Ponnusamy Pillai, M., Suppiah, P., Md Sharif, S., Krishnan, S. G., Samy Pullay, S., & Osman, N. A. (2020). The validation of the Malay Yale Food Addiction Scale 2.0:

factor structure, item analysis and model fit. *Public health nutrition*, *23*(3), 402–409. https://doi.org/10.1017/S1368980019002684

104.Sweeney, C. (2016). Screening for child and adolescent eating disorders in primary care settings. Fairleigh Dickinson University.

105. Uehara, T., & Oshima, K. (2014). Utility of one-item self-rating to screen for eating disorders: An investigation of Japanese university students. *Asian Journal of Psychiatry, 7*, 97–98. https://doi.org/10.1016/j.ajp.2013.08.003

106. Vander Wal, J. S., Waller, S. M., Klurfeld, D. M., McBurney, M. I., & Dhurandhar, N. V. (2005). Night eating syndrome: evaluation of two screening instruments. *Eating behaviors*, *6*(1), 63–73. https://doi.org/10.1016/j.eatbeh.2004.04.004

107. Walsh, J. M., Wheat, M. E., & Freund, K. (2000). Detection, evaluation, and treatment of eating disorders the role of the primary care physician. *Journal of general internal medicine*, *15*(8), 577–590. https://doi.org/10.1046/j.1525-1497.2000.02439.x

108.Zuijdwijk, C. S., Pardy, S. A., Dowden, J. J., Dominic, A. M., Bridger, T., & Newhook, L. A. (2014). The mSCOFF for screening disordered eating in pediatric type 1 diabetes. *Diabetes care*, *37*(2), e26–e27. https://doi.org/10.2337/dc13-1637
