# Supplementary material for: Validated strategies for screening for eating disorders in primary health care: A scoping review with a focus on adolescents and adults
Source: PLoS One. 2026 Aug 3;21(8):e0347184. doi: 10.1371/journal.pone.0347184 (PMC13432121; doi:10.1371/journal.pone.0347184)
Supplement: S1 Table — (PDF) [file pone.0347184.s004.pdf]

S1 Table. Characteristics of the empirical studies included in the review (n=56).

Author, year (country)	Study design Aim	Place of realization	Sampling: Sample size % Sample at risk (n)	Age (years) % female sex	Instrument (version) or methodological approach	Main results for the research questions
Al Hamimi, et al., 2024 (Oman)	Cross-sectional<br>Identify the significant sociodemographic factors associated with ED in Oman	Primary care antenatal clinics	Non probabilistic:<br>634<br>1.6% (n = 10)	MD: 30.7 (SD: 5.18)<br>100%	EDE-Q (Arabic version)	**RQ1:** 66.1% (n=419) of the sample had higher education and 82% (n=520) reported having a good family income.<br>**RQ2:** The screening was conducted by a family physician, through heteroadministration, during the consultation in the office. The questionnaire was translated into Arabic and a pilot test was performed.
Aoun et al., 2015 (Lebanon)	Cross-sectional<br>To evaluate the validity of the Arabic version of the SCOFF (A-SCOFF) for the detection of ED in primary health care settings.	Primary Health Care Units	Random:<br>123<br>42.3% (n=52)	Range: 15-55<br>100%	A-SCOFF (Arabic version validation)	**RQ1:** 91.1% (n=112) of the sample were Lebanese, 63.4% married (n=78), 78% Christian (n=96) and 60.2% (n=74) had relatively low monthly income (<1000 US$).<br>**RQ2**: The screening was conducted by an ED specialist, through self-administration, during the consultation, in the office. Translation, back-translation, and pilot testing of the instrument were performed.<br>**RQ3:** Structure validity: The principal component analysis generated 1 factor that explains 31% of the variability in the 5 questions (Kaiser–Meyer–Olkin value = 0.587 > 0.5; Bartlett sphericity test P = 0.007), which demonstrates the adequacy of the principal component analysis. This factor showed an excellent correlation with the A-SCOFF score, calculated as the sum of the 5 questions (r = 0.98, P < 0.001). Except for question 3, which had the lowest factorial weight (0.30), and question 2 (0.75), which had the highest, the similarity in the magnitude of the weights indicates a similar importance of the other questions. Criterion validity (compared to MINI): Sp: 80.0% (95%CI: 64.1-90.0%); Se: 72.7% (95%CI: 62.6-80.9%) AUC: 0.7951. Reliability: Cronbach's α: 0.43.<br>**RQ4:** 100% of the recruited subjects accepted the screening.
Aouad et al., 2024 (Australia)	Mixed Method<br>To evaluate the acceptability, feasibility, efficacy, and applicability of a *microlearning* program to train general practitioners on ED	Primary care	No probabilistic:<br>51<br>NA	Range: 25-60<br>NR	Microlearning	**RQ1:** The e-learning program consists of short, focused, spaced interactive lessons with vignettes with case series and questions, on ED, including tracking, designed by experts and researchers using the Qstream platform. Quantitative and qualitative responses revealed that the program was found to be effective in increasing knowledge, confidence, willingness, and skills in ED screening. Qualitative feedback highlighted the benefit of microlearning flexibility to train general practitioners to work with complex health presentations, specifically ED.
Backe, 2001 (Germany)	Cross-sectional<br>To assess the prevalence of ED in patients of a primary care gynecologist.	Primary Care Gynecologist Clinic	No probabilistic:<br>386<br>17% (n=66)	MD:34.7<br>100%	BITE (German version a)	**RQ1:** 47.1% (n=229) of the sample was married and 47.5% (n=231) were employed.<br>**RQ2:** The screening was conducted by the researcher, through self-administration, before the consultation, in the waiting room.<br>**RQ4**: 77.2% of the individuals recruited accepted the screening.

Baudet et al., 2003 (France)	Cohort<br>To analyze the value of screening with the SCOFF score in family medicine clinics with women who consult for reasons unrelated to body image, weight, or dietary concerns.	Primary Care Clinics	Non probabilistic: 143<br>21% (n=30)	MD: 32.9(SD: 9.2) 100%	SCOFF (French version validated[b])	**RQ1:** 72% (n=103) of the sample was married.<br>**RQ2:** The screening was conducted by the researcher, through self-administration, before or after the consultation.<br>**RQ3:** Criterion validity (compared to the DSM IV diagnostic interview): Sp: 65.2%, Se: 87.5%, AUC: 0.849.<br>**RQ4:** 95.3% of the recruited individuals accepted the screening.
Boulé; Mcsherry, 2002 (Canada)	Cross-sectionalTo assess the attitudes and behaviors of family physicians toward patients with ED, and to identify their ongoing learning needs.	Family Medicine Clinics	Non probabilistic: 20 family physicians<br>IN	≥ 25<br>36,1%	IN	**RQ4:** Regarding the experience with screening, 9.3% reported never having it, 24.9% doing it frequently, 25.4% routinely and 40.5% only when clinically indicated. Male physicians more frequently reported never performing screening or performing it only in the face of clinical suspicions (p = 0.02). Female physicians, on the other hand, were more likely to perform frequent or routine screenings, especially at periodic health checkups. They also demonstrated lower confidence in the presence of clinical signs and symptoms as the sole criterion for screening.
Chamay-Weber et al., 2017 (Switzerland)	Cohort<br>To investigate the performance of a questionnaire in predicting the diagnosis of binge eating, and to identify obese adolescents ED risk for BED during the consultation.	Department of Children and Adolescents of the University Hospitals of Geneva (environment generalizable to PHC)	Non probabilistic: 94<br>14.28%[d] (n=12)	Median: 14th; Range: 11-18<br>59.6%	ADO-BED (French version c validation)	**RQ2:** The screening was conducted by the researchers, through self-administration, after the consultation.<br>**RQ3:** Criterion validity (compared to SCID): If: 100% (for item 01 or 02) Variation between items: 33.3% (item 6) - 100% (item 01 or 02) Sp: 27.4% (for item 01 or 02) Variation between items: 20.8% (item 3d) - 87% (item 4).<br>**RQ4:** More than 90% of the eligible sample agreed to undergo screening.
Cotton, Ball, Robinson, 2003 (UK)	Cross-sectional<br>Validate the questions of the SCOFF clinical prediction rule in primary care patients and in a higher-risk population composed of university students; to evaluate the screening potential of 5 useful questions derived from other studies (ESP); compare the two instruments.	Primary Care Practice and University of London	Non probabilistic: 96<br>NR	MD: 37<br>Range: 22-64 to 77%	SCOFF (original version validated in clinical population[f])<br><br>ESP (creation and validation)	**RQ2:** The screening was conducted by the psychiatrist, through heteroadministration, while they were waiting in the waiting room (before the consultation).<br>**RQ3:** Criterion validity (compared to Q-EDD): SCOFF: Sp: 78% (95% CI 62-93); Se: 88% (95% CI 84-93%); ESP: Se: 100% (95% CI 90-100%); Sp: 71 (95% CI 64-77%).
Desocio et al., 2007 (USA)	Mixed method<br>Examine the methods used in primary care to screen for ED and identify opportunities to improve the early identification of these disorders.	Primary care and generalizable environment	Non probabilistic: 5 pediatricians<br>IN	NR	NA	**RQ4:** Clinical interviews were the primary tool for identifying ED, but only 50% of medical forms included questions about vomiting and exercise. Barriers to screening include professionals' reluctance to discuss risk factors, lack of time, delay in recognizing signs by adolescents and parents, need for a "discovery" process incompatible with short consultations, lack of confidence in interviews, inadequate referrals, limitations imposed by health insurance, excessive focus on obesity, absence of specific questionnaires, overload of services, and concealment of information by patients.
Diamond Allen, 2023 (USA)	Mixed method<br>Improve the knowledge, confidence, perception of importance, and screening of ED by professionals at	UCHC	Non probabilistic: 14 NA doctors and nurses	NR	Implementation of SCOFF (original version validated in clinical population[f])	**RQ2:** 15-min educational session on ED addressed tracking and referral, with materials distributed in person and by email (SCOFF and referral flowcharts).<br>**RQ4:** 46% of providers did not feel confident in screening, 43% did not perform it and 29% did not use any tools. Among those who adopted SCOFF, half reviewed or delivered

	UCHC.					it directly to patients; 7% had assistant medical support. The use occurred via electronic medical records (36%), printed version (28%) or both (7%).
Dorflinger, Ruser; Masheb, 2017 (USA)[i]	Cohort<br>To examine the validity, sensitivity, and specificity of a single-item tracking measure for binge eating in overweight/obese individuals, easily applied in primary care	Connecticut *Veterans Health System* (environment generalizable to PHC)	Non probabilistic: 116<br>22.41% (=26)	MD:61.66 (SD =8.73)<br>11,2%	VA-BES (creation and validation)<br>QEWP-R - (original version[g]<br>EDE-Q (original version validated in English[h])	**RQ1:** 93.1% of the sample was non-Hispanic;<br>**RQ2:** The screening was conducted by the researcher, through self-administration, during the weight management group sessions.<br>**RQ3:** Criterion validity of the VA-BES (compared to the QEWPR-R) Sp: 88.9%; Se: 83.2%.
Dorflinger, Ruser; Masheb, 2017 (USA)[i]	Cohort<br>To investigate Night Eating Syndrome (NES) among veterans seeking treatment for weight management, and the relationships between NES, weight, insomnia, disordered eating behaviors, and psychological variables.	Connecticut Veterans Health System (environment generalizable to PHC)	Non probabilistic: 110<br>11% (n=12.1)	MD: 61.6 (SD = 8.5)<br>10%	NEQ (original validated English version[J])	**RQ1:** Approximately 3/4 of the sample were white/Caucasian.<br>**RQ2:** The screening was conducted by the researcher, through self-administration, during the weight management group sessions.
Dukovic, 2021 (USA)	Mixed method<br>Improve the screening and recognition of EDs in the population that has recently reached adulthood and, consequently, their outcomes.	Seattle University Student Health Center (environment generalizable to PHC)	Non probabilistic: 115 students and 4 nurses 5.2% (n=6)	NR	Implementation of SCOFF (original version validated in clinical population[f])	**RQ2:** 1-hour educational session addressed tracking and referral of EDs, with materials such as SCOFF and referral algorithm available. Professionals were encouraged to apply screening before the consultation, through self-administration. The training has increased confidence, comfort, and knowledge about tools and routing.<br>**RQ4:** After positive screening, they followed the referral protocol. All of them found SCOFF easy, useful, and recommend its regular use. The main barrier was time. On a Likert scale (0-5), the average knowledge about ED screening was 3.33 (SD = 0.94).
Flahavan, 2006 (Ireland)	Cross-sectional<br>Identify current trends and deficiencies in the screening, diagnosis, and management of EDs in primary care in Ireland.	Primary care	Non probabilistic: 91 physicians<br>IN	NR	IN	**RQ4:** Regarding the experience of the professionals, only 17.6% (n=16) perform routine screening for ED and the absence of a systematic screening process was identified as a barrier.
Garcia-Campayo et al., 2005 (Spain)	Cross-sectional<br>To evaluate the validity and clinical utility of the Spanish version of the SCOFF for the detection of EDs in primary care settings.	Six primary care centers	Non probabilistic: 203<br>NR	MD: 29.2 (SD: 7.9)<br>Range 15 - 53<br>100%	SCOFF (Validation of the original version in Spanish)	**RQ2:** The screening was conducted through self-administration, lasting approximately three minutes. Translation, back-translation, pilot test and review by the final authors were performed.<br>**RQ3:** Criterion validity (compared to SCAN): Sp: 97.7% (95% CI 93.5–99.5), Se: 94.4% (95% CI 86.4–98.5), AUC: 0.947; Reliability: intraclass correlation coefficient: 0.97 (95% CI, 0.96–0.98).<br>**RQ4:** 100% of the recruited subjects accepted the screening.
Giuffrida, 2020 (USA)	Cross-sectional<br>Develop an enhanced, evidence-based screening tool to effectively detect binge eating in primary care.	Primary care office in internal medicine and pediatrics	Non probabilistic: 100<br>17% (n=17)	Range: 18-65 to 67%	BEDS-7 - (original version validated in English[K])	**RQ2:** The screening was conducted by the health professional, through self-administration, during the consultation, in the office.

Gooding et al., 2017 (USA)	Quasiexperimental<br>To evaluate the ability of two educational interventions to increase ED screening in pediatric primary care practice, hypothesized that the "active learning" group would have an increase in documented screening after the intervention.	Pediatric primary care practice	Non probabilistic: 303<br>IN	NR<br>72,2%	Training: Active Learning vs Print Learning	**RQ2:** Professionals were divided into two groups: active learning (interactive lecture and online education about the AED Guide) and printed learning (reading the AED Guide). Before the intervention, 4.5% of the patients had screening. Afterwards, the active group reached 22% and the printed group 5.7%, with a significant difference. The active group also showed greater knowledge, comfort in the diagnosis and satisfaction with the training.<br>**RQ4:** Regarding the perception and experience of the professionals, the self-reported screening rates ranged from 45.6% to 65.9%, while the documented rates ranged from 4.5% to 4.7%, showing a large discrepancy. In high-risk groups, self-reported rates exceeded 80%, and documented rates were between 3.2% and 14.3%. More than 98% of professionals reported asking about diet and exercise at routine pediatric visits.
Gurney; Halmi, 2001 (USA)	Quasiexperimental<br>Evaluate the effectiveness of an ED curriculum designed to address the lack of knowledge among primary care professionals.	Six medical clinics Community	Non probabilistic: 9 social workers<br>IN	NR<br>100%	Intensive training brief "eating disorder curriculum"	**RQ2:** The training took place in 4 weeks, 1x/week, lasting 75 minutes, addressing ED, risk factors and assessment. There was a 22% increase in professionals who screened more than 20% of new patients for ED, and a significant improvement in knowledge about evaluation and treatment. After training, 4 of the 7 social workers identified patients with ED, compared to only 1 in the previous 12 months.
Hariri, 2023 (Arabia welcomes)	Cross-sectional<br>To investigate whether adolescents who visited health centers with their parents in Mecca, Saudi Arabia, were at high risk for EDs.	Eight primary health care centers	Random: 428<br>55.2% (n=237)	Median: 20.0<br>Range: 15-24 to 72.2%	A-SCOFF (Arabic version validated[l])	**RQ2:** The screening was conducted by the researcher, through an online self-administration questionnaire via QR code *(query code).*<br>**RQ4:** 100% of the recruited subjects accepted the screening.
Hautala, et al., 2009 (Finland)	Cross-sectional<br>To assess the feasibility of the Finnish version of the SCOFF questionnaire in screening for symptoms of EDs among adolescents	School health service (environment generalizable to PHC)	Non problabilistic: 1891 students and 14 school nurses<br>21% (n=204)	Students:<br>Range: 14-16A<br>54,3%<br><br>Nurses:<br>Range: 35-57A<br>100%	SCOFF (Finnish version validation)	**RQ2:** The screening was conducted by the nurses, through self-administration, during the health assessment. Translation and back-translation were performed.<br>**RQ3:** The validity of the SCOFF was evaluated by confirmatory factor analysis. Tetraicoric correlations between items were lower among younger participants (S1: 0.026–0.457) and boys (S12: 0.095–0.515), and higher among older adolescents (S2: 0.264–0.787) and girls (S12: 0.340–0.627). The factor loadings ranged from 0.36 to 0.98 in the M1 model and from 0.39 to 0.99 in the M2 model, with correlations between factors above 0.70. Both models showed a good fit to the data from S1 and S2, with the performance of M2 being slightly higher than that of M1 in S2. For girls, M2 fit better than M1, while among boys there was no difference, suggesting an effect of age on the factorial structure of the instrument.<br>**RQ4:** Of the students, 69.7% returned the completed questionnaire. Among those who self-reported ED symptoms in the SCOFF, 81% were not identified in health examinations without the use of a specific questionnaire.
Hay; Marley; Lemar, 1998 (Australia)	Cross-sectional<br>To investigate the prevalence and characterize patients with bulimic disorders who attended a large family medicine practice in South Australia; explore the type and quality of help sought.	General clinics linked to the university (environment generalizable to PHC)	Non probabilistic: 500<br>12.6% (n=63)	MD: 35A (SD: 10.7);<br>Range: 16-59A<br>100%	EDE-Q (original version validated in English[h])	**RQ2:** The screening was conducted by the researcher, through self-administration, before the consultations, in the waiting room.<br>**RQ4:** Responses were evaluated and those who were at risk (n=63) were invited for an interview (EDE) within two weeks.

Hay; Loukas; Phillpott, 2005 (Australia)	Cross-sectional<br>To assess the prevalence, characteristics of EDs, weight, comorbidities, exercise patterns, sexual orientation, and access to help services in a larger sample of men in general practice, and to compare the findings with a similar study conducted by Hay with women in 1995.	02 general clinics	Random:<br>383<br>19.5% (n=75)	MD: 41 (SD: 11.8)<br>Range: 19 - 64<br>0%	EDE-Q (original version validated in English[h])	**RQ1:** 35% of the sample was married and 66% was heterosexual.<br>**RQ2:** The questionnaire was sent by mail, and was self-administered at the subject's home.<br>**RQ4:** 77% (n=383) of the individuals returned the completed questionnaire. Those considered at risk for ED were invited to an interview (EDE) (n=75).
Henderson, 2024 (USA)	Mixed method<br>Develop a practice change to universally implement an evidence-based screening tool for adolescents aged 13-18 years in a pediatric primary care clinic.	Pediatric primary care clinic linked to the university (environment generalizable to PHC)	Non probabilistic:<br>20 users and 58 physicians<br>30% (n=6)	Range: 13 -18<br>NR	Implementation of SCOFF (original version validated in clinical population[f])	**RQ1:** A resource package was made available to the professionals that included the SCOFF, a report on the tool and a treatment and referral protocol with a flowchart. In addition, they were encouraged to conduct the screening, self-administered, starting at the reception and ending in the office. The screening time ranged from 1 min for negative cases to up to 5 min for positive.<br>**RQ4:** 100% of adolescents accepted SCOFF screening. After the positive result, the professionals made the referral, according to the established protocol. The SCOFF tool was user-friendly, effective, fast, and user-friendly for both patients and professionals.
Herman et al., 2017 (USA)	Cohort<br>To assess clinicians' knowledge and attitudes about BEDS-7 and the value and ease of use of BEDS-7 in clinical practice.	Primary Care and Psychiatry [e]	Random:<br>122<br>NA	NR<br>30,3%	BEDS-7<br>(original validated version in English[K])	**RQ1:** 66.4% of the sample of professionals (n=81) were white.<br>**RQ4:** Approximately 32% of adult professionals have used BEDS-7, finding it valuable and easy to use. Most applied it to 1-9 patients, with about half showing positive results. Among the professionals with positive cases (n=29), 44.8% underwent additional evaluations and 65.5% diagnosed BED. Many indicated that they would continue to use it. Forgetting the availability of the scale, prioritizing other patient health issues and lack of time were pointed out as barriers.
Herscovic; Bay; Kovalskys, 2005 (Argentina)	Cross-sectional<br>To investigate the prevalence of clinical ED and to assess the presence and severity of unhealthy eating habits and attitudes in a random population sample of adolescents aged 10 to 19 years in primary care.	Primary care	Non probabilistic:<br>1971<br>19.2% (n=380)	Range: 10-19<br>62,5%	EDEQ-4 (original validated English version[h])	**RQ1:** All were white and predominantly from the middle and lower-middle classes.<br>**RQ2:** The screening was conducted by the pediatrician, through self-administration, except in individuals <13 years old or with difficulty in understanding (heteroadministration).<br>**RQ4**: Positive cases were scheduled for an interview with pediatricians, where they were administered EDE.
Johnston et al., 2007 (UK)	Mixed method<br>To evaluate the feasibility of screening for disordered eating behaviors in primary care: proportion of patients who accept screening, number of cases identified, actions of professionals and their perceptions.	Two primary care clinics	Non probabilistic:<br>111 users and 11 professionals<br>16% (n=18)	MD: 26.0; Range: 16-35<br>100%	SCOFF (original version validated in clinical population[f])	**RQ1:** Most patients were white (96.2%) and had a paid job (62.9%).<br>**RQ2:** The screening was conducted by the researcher, through self-administration, before the consultation, in a quiet area.<br>**RQ4:** 46% of eligible individuals have accepted screening. Only 11% of the positive cases registered intention to treat or refer, and only four patients had documented medical notes. Screening was considered acceptable and useful, but it was not routine in consultations. The SCOFF was well accepted, but there was variation in the comfort of the professionals in addressing the theme. Barriers included: cultural emphasis on thinness, doubts about screening effectiveness, timing, financial and administrative constraints, uncertainty about the benefits of early diagnosis, lack of appropriate referrals, limited expertise, and patient resistance.

Keane et al., 2017 (Ireland)	Cross-sectional<br>To present the prevalence of eating behaviors characteristic of ED and to establish norms for EDE-Q among university students in Ireland.	University primary health care service (generalizable environment to PHC)	Non probabilistic: 190<br>5.28% (n=10)	MD: 21.9 (SD: 4.2)<br>Range: 18 - 30<br>100%	EDE-Q (6.0 m version)	**RQ2:** The screening was conducted by the health professional, through self-administration before the consultations, in the waiting room.
King, 1989 (United Kingdom)	CohortAnswer the following questions: (a) What is the prevalence and clinical profile of EDs in patients in general practice? (b) What associated psychological characteristics may be indicative for the physician? (c) Can the EAC be useful as a tracking tool? (d) What is the natural history of ED in the community? (e) Can doctors recognize and manage these disorders?	4 general clinics	Non probabilistic: 720<br>10.5% (n=76)	Range: 16 - 35<br>74,2%	EAT-26 - (original version validated in English[n])	**RQ1:** 46.1% (n=332) of the sample was married.<br>**RQ2:** The screening was conducted by the researcher, through self-administration, before the consultation.<br>**RQ3:** Criterion validity (compared to *the Eating Interview*): Se: 100% Sp: 91%. RQ4: 96% of the sample accepted screening. The entire at-risk sample was approached for semi structured interviews (CIS *and Eating Interview*) within one to two weeks after screening. After the interview, there were eight referrals to a psychiatrist and seven psychotropic treatments by the family doctor.
Klein et al., 2001 (USA)	Quasiexperimental<br>Evaluate the implementation of GAPS in Community Mental Health Centers (CMHCs).	Five CMHCs	Non probabilistic: 81 professionals and 649 users<br>NA	NR	Training for GAPS implementation	**RQ2:** Three professionals per center have been trained to implement GAPS. Physician-self-reported screening increased from 57% to 76%, and patient reporting rose from 11% to 28% ($p < 0.001$). Documentation in medical records increased from 0.3% to 75% ($p < 0.0001$).<br>**RQ4:** Professionals' self-reported screening rates were 57% and documented 0.3%.
Lebow et al., 2021 (USA)	Mixed method<br>Investigate in depth the perspective of primary care professionals on the challenges in identifying and managing ED.	Six primary care clinics	Non probabilistic: Quantity:60<br>Qualitative: 16<br>NA	NR	NA	**RQ4:** The professionals demonstrated moderate confidence in the ED assessment, with a mean of 5.53 on a 10-point scale (SD 2.09). Barriers included: lack of time, perception of ED as less frequent problems, focus on other health issues, lack of validated and effective screening tools for pediatrics, and difficulties in implementing universal screening in short visits.
Linville et al., 2010 (USA)	Mixed method<br>Investigate the prior training, needs, and screening and intervention practices of health professionals.	Primary care	Random: Quantitative: 183<br>Qualitative: 12<br>NA	Range: 26 - 40 (50% of participants)<br>70%	NA	**RQ1:** 100% of the professionals in the qualitative sample were Caucasian.<br>**RQ4**: 54% of providers agreed with universal ED screening in all patients. The qualitative analysis showed the barriers and facilitators of screening that include: difficulties in treatment, lack of options, limited time, feeling of powerlessness and discomfort, insecurity in the questions, lack of knowledge and training, sociocultural myths, weighing management and lack of knowledge of the role in the multidisciplinary team, and as facilitators: questions appropriate to the role of the provider, brief screening tools, training and continuing education in ED, collaborative forums, multidisciplinary team, and referral sites.
Linville et al., 2012 (USA)	Quasiexperimental (mixed method)<br>To investigate the longitudinal effectiveness of a brief training on ED on the perception of knowledge, skills, and attitudes of primary care	Ten primary care clinics	No probabilistic: Quantity: 45<br>Qualitative: 5<br>NA	NR<br>62%	Implementation of the EDQ Screen tool - (adapted from the original version)	**RQ2**: The 60 - 75 min educational presentation addressed TA, including screening, with the provision of a resource package that included a referral list and the *EDQ Screen tool* (16-item adaptation of the EDDS).<br>**RQ4:** Regarding the frequency of screenings, among the 53% (n=24) of the professionals who responded, 12.5% (n=3) reported never performing them, 37.5% (n=9) rarely, 33.3%

	professionals in relation to screening and intervention.					(n=8) sometimes and 16.7% (n=4) frequently. The ability to conduct ED screening was considered low or very low by 62% of the respondents. In addition, 40% agreed that universal screening is appropriate, regardless of the patient's complaint. The qualitative analysis highlighted as barriers the overload of medical practice, the limited time, the bureaucracy involved and the need for multiple evaluations. As a facilitator, the regular performance of screening through the EDQ Screen tool was mentioned.
Linville, Brown, O'NEIL, 2012 (USA)	Cross-sectional<br>To investigate the perception of health professionals in the USA about their knowledge, skills, and needs in ED screening and intervention.	Primary care	Random:<br>260<br>IN	Range: 41 - 60 (60% of participants)<br>76.8%	IN	**RQ1:** 87% of professionals were white or Caucasian.<br>**RQ4:** Regarding barriers to ED screening, 68% of professionals reported not prioritizing it due to other clinical demands. All participants indicated a lack of specific training in ED, expressing a feeling of unpreparedness to deal with these cases. In addition, 41% pointed out that the training received was insufficient to carry out adequate screenings. Only 4.94% considered ED screening to be a complex task.
Lovey, 2023 (USA)	Cross-sectional<br>Create a new instrument to investigate behavioral factors that influence whether professionals evaluate eating disorders; validate and test its reliability.	Primary Care and Psychiatry [e]	Non probabilistic:<br>173<br>IN	Range: 41 to 50a (majority)<br>80.9%	IN	**RQ1:** 72.2% of the professionals declared themselves white.<br>**RQ4:** 12.1% of the professionals (n=21) reported that they perform the screening, 46.2% agreed or strongly agreed that evaluating the BED was satisfactory, 22.5% agreed that other professionals expect them to perform the BED screening, and 34.1% felt confident in evaluating the BED. Barriers were identified as missing screening tools, inadequate education, unfamiliar resources, lack of time, and the need for more training.
Luck et al., 2002 (UK)	Cross-sectional<br>To evaluate the use of the SCOFF questionnaire in primary care.	Two general practices	Non probabilistic:<br>341<br>13.2% (n=45)[d]	Range: 18 -20<br>to 100%	SCOFF (Validation of the original version in primary care)	**RQ2:** The screening was conducted by the researcher, through self-administration, in a separate room, lasting 2 minutes.<br>**RQ3**: Criterion validity (compared to DSM IV diagnostic interview): Sp: 84.6% (95% CI 54.6 to 98.1%), Se: 89.6% (CI: 86.3 to 92.9%)
Lévêque; Boyer; Casterà, 2022 (France)	Qualitative<br>To explore the representations of general practitioners about ED, the barriers to addressing the topic in practice, and to understand the underreporting of these disorders.	Primary care outpatient clinics	Non probabilistic:<br>23<br>NA	NR	NA	**RQ4:** The detection of TA was mainly visual, based on silhouette or weight considered abnormal. Barriers included uncertainty about how to approach the topic, lack of knowledge of screening tools, time, feeling of lack of communication and psychology skills, and fear of altering the doctor–patient relationship. In addition, general practitioners were unaware of screening tools, such as SCOFF-F, validated in France.
Maguen et al., 2018 (USA)	Cross-sectional<br>Develop a screening tool for ED in primary care with greater precision and potential for generalization than the existing ones.	Veterans Health Administration (USA) medical center (environment generalizable to PHC)	Non probabilistic<br>402<br>15.6% (n=63) by the EDE-Q and 51% (n=205) by the SDE	MD: 49 - 51.7 (SD: 12.5 - 13.1) 100%	SCOFF (original version validated in clinical population[f])<br>SDE (Creation and Validation)<br>EDS-PC (original validated English version[p])<br>EDE-Q (original version validated in English[h])	**RQ1:** 60% (n=241) were Caucasian, 38.5% (n=155) were Caucasian Separate (a) or divorced or widowed and - 24.88% (n=100)<br>**RQ2:** The instruments were sent by mail, being self-administered at the subject's home.<br>**RQ3**: Criterion validity (compared to EDE-Q): SCOFF: Sp: 66.1% (95% CI 53.0–77.7%); Se: 79.9% (95% CI 75.2–84.0%); AUC: 0.730 (95% CI: 0.667–0.793)/SDE: Sp: 90.5% (95% CI 80.4–96.4); Se: 57.5% (95% CI 52.1–62.8%); AUC: 0.740 (0.695–0.785)/EDS-PC: Se: 96.6% (95% CI 88.1–99.6%); Sp: 40.3% (95% CI: 35.0–45.8%); AUC: 0.684 (95% CI 0.649–0.720).<br>**RQ4:** 99% accepted the tracking by returning the envelopes.
Malson et al., 2022 (UK)	Qualitative<br>To offer a multiperspective qualitative analysis on the experiences and views of three key	Primary care	No probabilistic:<br>41<br>NA	NR<br>90%	NA	**RQ4:** The following barriers to ED screening have been described: high workloads; consultation time for complex problems (10 min); unfamiliarity and low knowledge; lack of resources to manage; absence of training; difficult communication with young patients.

	interest groups (general practitioners)					
Maradiegue et al., 1996 (USA)	Cross-sectional<br>To determine whether there are differences in the nutritional assessment for the detection of EDs when performed by nurses, physician assistants, or physicians.	Three family medicine clinics	Random:<br>13<br>NA	NR	NA	**RQ4:** The experiments showed that nursing professionals perform more complete screening than physicians (p = 0.01), but without significant difference compared to physician assistants (p = 0.49). The analysis of variance confirmed this superiority of the nurses' screening skills in relation to the physicians (p = 0.01).
McClure, 2020 (USA)	Mixed method<br>Improve the early identification and management of atypical eating disorders through: 1) the use of an evidence-based tool for screening and early diagnosis in adolescents at risk of or with symptoms of BED; 2) appropriate use of evidence-based clinical management pathways.	Family Medicine Care Clinic	No probabilistic: 7 users and 4 professionals<br>71.4% (n=5)	Users<br>MD: 16a; Range: 14- 18 to 43%	Implementation of screening *(Binge Eating Disorder Screening Tool*[q])	**RQ2:** The 20-minute educational presentation addressed the role of professionals in the early identification of ED. The intervention lasted 8 weeks, focusing on the use of a screening tool and case management algorithm. The resource package included educational websites and assessment tools and management algorithms. There was a 9% increase in the knowledge and awareness of professionals after the intervention. Professionals were encouraged to conduct screening through heteroadmnistration during the consultation.<br>**RQ4:** Only one patient with a positive result was scheduled for four-week follow-up, while the others for annual follow-up. No patient was referred to psychotherapists or nutritionists. None of the nurses used the tool, despite considering it useful. Barriers identified: shame of adolescents and time to apply the tool. Facilitators: Professionals found the tool useful for behavioral screening.
Mond et al., 2008 (USA)	Cross-sectional<br>To compare the validity of the EDE-Q and SCOFF in screening the most common ED in a primary care sample in the USA.	Primary Care	Non probabilistic:<br>257<br>17.5% (n=45)	MD: 27.6 (SD: 6.50)<br>Range:18 - 40a 100%	SCOFF (adapted version from the original version[f])<br><br>EDE-Q (reduced version - 22 r items)	**RQ1:** 87.9% of the sample was Caucasian, 45.6% were married, 38.5% were full-time employed.<br>**RQ2:** The screening was conducted by the receptionist, through self-administration, lasting 30 seconds (SCOFF) and 2 to 4 min (EDE-Q). Minor changes have been made to the original version of SCOFF to accommodate the U.S. population.<br>**RQ3:** Criterion validity (compared to EDE): SCOFF: Sp: 72%; Se: 73% AUC: 0.80/EDE-Q: Sp 80%; SE 80%/AUC: 0.85/Reliability: SCOFF: Cronbach's α: 0.44/EDE-Q: Cronbach's α: 0.96.<br>**RQ4:** 51.4% of the individuals returned the completed forms.
Nicholas, 2023 (USA)	Cross-sectional<br>Identify needs and strategies to improve access to diagnosis and treatment of ED through a state board in a resource-poor region.	Primary Care	Non probabilistic:<br>33<br>IN	NR	NA	**RQ4:** Among the participating physicians, 69.7% reported screening for ED, with clinical interviews being the most commonly used method. Approximately 34.8% relied on written self-report as a screening strategy, and 60.6% stated that they felt comfortable conducting this process. The barriers reported included uncertainties regarding screening, lack of knowledge about ED, doubts related to treatment and referral, and discomfort when addressing symptoms with patients. Although 93.9% of the 33 physicians received some type of formal education on screening, only 24% reported using specific tools on a regular basis, while the others demonstrated limited knowledge about how and when to apply them.

Noble, 2023 (USA)	Mixed method<br>Increase professionals' knowledge of ED, screening, and SCOFF use, through the implementation of an educational presentation for pediatric primary care professionals.	Pediatric primary care office	Non probabilistic: 5 IN	NR	Evidence-based educational presentation (training)	**RQ2:** 10-minute educational video on ED, screening, and SCOFF, based on AAP recommendations. Knowledge about ED screening increased from 33% in the pretest to 82% in the posttest, especially about the SCOFF.<br>**RQ4:** 100% of participants do not use a screening tool. About knowledge, in the pretest (before the intervention). 20% were correct about the frequency of screening, 60% about who should be screened and 40% about the next steps when suspecting a case, all of whom were wrong with the SCOFF score.<br>Barriers identified: time, remembering the SCOFF questions and scoring, patients' reluctance to answer, and forgetting the tool.
Nowaskie et al., 2021 (USA)	Cross-sectional<br>To assess the relationship between gender identity, gender-affirming interventions (such as hormone therapy and surgeries), and symptoms of EDs.	Outpatient Gender Health Program in Primary Care	Non probabilistic: 166<br>23.9% (n=40)	MD: 31.11 (SD: 13.05)/52.4% transgender woman	EDE-Q (version 6.0 s)	**RQ1:** 71.1% (n=118) were Caucasian/white.<br>**RQ2:** The screening was conducted by the health professional, through self-administration, during the consultation, in the examination room.
Nunes et al., 2014 (Brazil)	Cohort<br>Assess ED symptoms from the pregestational to postpartum period.	Basic health units	Non probabilistic: 427<br>2.5% (n=11)	Range: 13-42<br>100%	EDE-Q (validated and adapted version in Portuguese[t])	**RQ1:** 64% of the sample had a low level of education (0-8 years of schooling), 60% were unemployed and 70% had a low family income (1-3 minimum wages)<br>**RQ2**: The screening was conducted by researchers or trained interviewers, through heteroadministration, before or after the consultations, in a space available by the health center. The instrument was adapted to Brazilian Portuguese due to variations in grammatical structure and word meanings.
Pérez Martin et al., 2021 (Mexico)[u]	Cross-sectional<br>Knowing the percentage of adolescents at risk of developing an ED; to identify the prevalence of risk for anorexia, bulimia and ED not specified according to age, sex, health center, nationality and family data; to determine the percentage with subclinical forms and to study the feasibility of using early detection tools in primary care.	Five health centers	Random: 291<br>12.4% (n=36) by SCOFF<br>4.5% (n=13) by EAT-26 and<br>12% (n=35) EDE-Q 6.0	MD: 16.3 (SD: 1.6)<br>Range: 14 - 19<br>57,4%	SCOFF (Spanish version v)<br>EAT-26 (Spanish version validated[w])<br>EDE-Q 6.0 (Spanish version validated[x])	**RQ2:** The screening was conducted by the researcher, through self-administration, with a duration of 3.98 (±0.12) min (SCOFF), 5.15 (±0.31) min (EAT-26), 15.23 (±1.23) min (EDE-Q).<br>**RQ3:** Criterion validity (compared to EDE-12): SCOFF Sp: 91.6% (95%CI: 87.2-95.8), Se: 91% (95%CI: 87.5-94.5)/EAT-26 Sp: 25% (95%CI: 20.8-30.1), Se: 96.4% (95%CI: 94-98.7)/EDE-Q 6.0 Sp: 99.3% (95%CI: 96.2-100), Se: 91.7% (95%CI: 88.3-95.1) Reliability α of Cronbach ≥ 0.74<br>**RQ4:** Those with positive results were invited to an in-person consultation to perform EDE-12, conducted by a specialized family physician
Pérez Martin et al., 2022 (Mexico)[u]	Cross-sectional<br>To evaluate the psychometric properties of the SCOFF questionnaire as an instrument for rapid and reliable detection of ED in a primary care unit.	Five health centers	Random: 291<br>12.4% (n=36)	MD: 16.3 (SD: 1.6); Range: 14 - 19<br>57,4%	SCOFF (Spanish version v)	**RQ2:** The screening was conducted by the researcher, through self-administration, with a duration of 3.98 (±0.12) min.<br>**RQ3:** Criterion validity (compared to EDE-12): Sp: 91.6% (95% CI: 71.8–100), Se: 91% (95% CI: 87.5–94.5), AUC: 0.9099 (95% CI: 0.8082–1.0117), α-Combrach*'s reliability:* 0.725<br>**RQ4:** Those with positive results were invited to an in-person consultation to perform EDE-12, conducted by a specialized family physician
Raffoul et al., 2022 (USA)	Experimental<br>Develop and test the effectiveness of an online training to improve comfort, knowledge, and behaviors related to ED screening among	Primary Care	Random: 84<br>NA	Range: 18 to 69a<br>92.8% (n=78)	Training: Spaced education continued *versus* no spaced education	**RQ1:** 81.9% (n=68) of the sample was white.<br>**RQ2:** Professionals were divided into two groups: spaced education continued with asynchronous video (1 h) on screening and referral and spaced education prompts (questions) via email every two days and no spaced education (video only). There were significant improvements in the knowledge and comfort of all professionals in relation to

	pediatricians in the US.					screening and referral. However, participants in spaced education reported significantly higher comfort in screening for BN ($p < 0.01$) and BED ($p < 0.01$) compared to participants who did not have spaced education.
Robinson; Boachie; Lafrance, 2012 (Canada)	Cross-sectional<br>Describe the key practices of primary care clinicians in Ontario, Canada, on screening, assessment, and treatment of ED in children and adolescents.	Primary Care	Non probabilistic: 153<br>NA	NR	IN	**RQ4**: Approximately 62% of professionals reported frequently including questions about eating behaviors in their clinical evaluations, while only 16% reported consistently screening for ED. Only 5% indicated that they never perform screenings. A difference in the emphasis of the identified symptoms was observed: psychologists reported greater recognition of psychological symptoms, while physicians more frequently highlighted physical symptoms.
Reid, Williams; Hammersel ey, 2010	Qualitative<br>To describe the results of qualitative interviews with general practitioners about their experiences and opinions about patients with eating disorders.	Primary Care (including university health service)	Non probabilistic 20<br>NA	NR	NR	**RQ4:** Physicians have shown a preference for referring their patients with suspected ED to specialized services. However, long waiting times in these care networks often led primary care providers to try to manage cases on their own. The main barriers reported included the lack of experience and knowledge to identify EDs, the difficulty of patients in recognizing or accepting that they have a problematic relationship with diet — which can lead them to hide the symptoms — in addition to the frequent presence of comorbidities that hinder the identification and appropriate management of these disorders.
Sayed Ahmed et al., 2024 (Egypt)	Cross-sectional<br>To explore the prevalence and factors associated with SEN, insomnia, and psychological distress in people with obesity, in order to plan comprehensive interventions.	Five primary health care centers	Non probabilistic: 386<br>22% (n=85)	Range: 18-65A NR	NEQ (Arabic Validated Version[y])	**RQ2:** The screening was conducted by the family doctor, through heteroadministration (interview)
Silva, 2012 (Portugal)	Cross-sectional<br>To analyze women with normal weight and overweight/obesity in terms of eating behaviors, physical exercise, and psychological well-being.	Family Health Unit	Non probabilistic: 294<br>NR	≥ 40A<br>100%	EDE-Q (validated and adapted version in Portuguese[t])	**RQ2:** The screening was conducted by the researcher, through self-administration.<br>**RQ4:** 100% of the sample filled out the questionnaire
Soares et al., 2009 (Brazil)	Cross-sectional<br>To investigate the prevalence of inappropriate eating behaviors and associated factors among pregnant women in primary care.	18 primary care units	Random: 712<br>0.6% (n=4)	MD: 24.7 (SD = 6.4)<br>Range: 13–42<br>100%	EDE-Q (validated and adapted version in Portuguese[t])	**RQ1:** 49.4% of the sample (n=352) had a low level of education (5-8 years of schooling), 59% (n=420) did not work/study, 52% (n=370) had an income of 1.01-3.00 minimum wages.<br>**RQ2:** The screening was conducted by a trained interviewer, through heteroadministration, at the subject's home, lasting 15 min. The instrument was adapted to Brazilian Portuguese due to variations in grammatical structure and word meanings.<br>**RQ4:** 91.3% of the sample completed the interview.
Suder, 2011 (USA)	Mixed method<br>To assess the acceptability of primary care professionals to	Primary Care Clinic	Non probabilistic: 100 users and 6	Patients: >18<br>100%	Implementation of SCOFF (original version validated in	**RQ1:** The resource package made available to professionals included SCOFF, an algorithm for ED, summarized NICE guidelines, and a link to the screening survey. The screening was conducted by the coinvestigator, through self-administration, before the

	implement an ED screening tool.		professionals<br>3% (n=3)	Professionals:<br>>21<br>67%	clinical population[f])	consultation.<br>**RQ4:** None of the professionals evaluated performed routine screening for EDs, and only two considered it useful to continue using the SCOFF instrument. None of the participants reported that the tool effectively contributed to the diagnosis. Among the reasons given for low adherence, the excess of screening instruments available, the perception of low prevalence of ED in the community served, and the lack of knowledge on how to identify these disorders stood out.
Tan; Spector-Hill, 2021 (UK)	Cross-sectional<br>Understand the level of knowledge and confidence of health professionals in Wales about clinical presentations of ED with T1D, its identification and treatment.	Primary care, diabetes and ED services [e]	Non probabilistic:<br>44<br>NA	NR	NA	**RQ4:** Regarding the level of confidence of professionals in identifying ED in people with DM1, 13.6% (n=6) were "not at all confident", 61.4% (n=27) "not very confident", and 25% (n=11) "very confident". None declared themselves "very confident". Regarding the use of screening instruments, 90.9% (n=40) did not use any, while 9.1% (n=4) used the SCOFF questionnaire.
Thom, 2021 (USA)	Mixed method<br>To investigate the perception of primary care professionals in a rural clinic about the implementation of ED symptom screening in female adolescents aged 11 to 19 years.	Primary Care	Non probabilistic:<br>81 users and 14 professionals<br>25.9%[d] (n=23)	Users: Range: 11-19 to 100%	Implementation of the SDE	**RQ2:** The SDE tool was initially explained by e-mail and complemented with face-to-face training, which addressed ED, prevalence, screening, and the use of the instrument. A form was also delivered to record the referrals, and the screening was conducted with the support of the nursing team.<br>**RQ4:** Only 14.1% of the patient sample completed the screening. Those with a positive result were referred to a counselor, with a referral rate of 17.4% (n=4). Among the professionals, 85% (n=12) considered the SDE tool easy to use, and 71% (n=10) believed that it is effective in identifying disordered eating patterns. However, 28% (n=4) remained neutral regarding its accuracy and 42% (n=6) were indifferent to the benefits of the tool.
TSE et al., 2022 (Canada)	Qualitative<br>Identify the learning needs and challenges faced by Canadian family physicians and residents in the care of patients with ED.	Department of Family Medicine of a medical school (environment generalizable to PHC)	Non probabilistic:1 1<br>NA	NR<br>72,7%	NA	**RQ4:** Barriers identified in screening: Communication failures, need for strategies to identify patients with suspected ED, hesitancy to address the topic, lack of knowledge about screening criteria, difficulty identifying disordered patterns without typical signs, uncertainty about signs and symptoms, not considering warning signs such as bariatric surgery, limited consultation time, and challenges in referral due to financial and financial barriers Geographical. Facilitators identified: Knowledge of different screening strategies.
Wade et al., 2022 (Australia)	Mixed Method<br>Investigate an approach to increase ED screening and its impact on referrals for treatment.	Family doctors' offices	Non probabilistic:<br>73<br>NA	NR	3-element tracking initiative (SCEDAT protocol; Promotion of the use of SDE[z] (original version) + training	**RQ2:** Professionals were reminded of the SCEDAT protocol for individuals at risk for ED, promoted the use of SDE on medical desktops (with variation in the tools used), and provided written information on screening and training. Referrals increased from 2.8 to 8.2 patients/month, with team assignment within 48 hours and evaluation within 2 weeks.<br>**RQ4:** Barriers to screening: discomfort when addressing eating habits, limited time and absence of referral flow. Facilitators in screening: SDE facilitates conversations and there is a basic routing model.

Acronyms: AAP (American Academy of Pediatrics), PHC (Primary Health Care), ABED (BED in Adolescents), AED (Academy for ED), AUC (Area Under the ROC Curve), AFC (Confirmatory Factor Analysis), CMHCs (Community and Migrant Health Centers), ED (Eating Disorder), EDE (Eating Disorder Examination), EDEQ (Eating Disorder Examination Questionnaire), ESP (Screening for Primary Care ED), EAT (Eating Attitudes Test), GAPS (Guidelines for Preventive Services in Adolescents), GP (General Practitioner), NA (Not Applicable), NES (Night Eating Syndrome), NR (Not Reported), Q-EDD (Questionnaire for Diagnosis of ED), QEWP-R (Dietary and Weight Patterns Questionnaire), SCAN (Schedule for Clinical Evaluation in Neuropsychiatry), SCID (Structured Clinical Interview for DSM Disorders), Sp (Sensitivity), Se (Specificity), UCHC (United Community Health Center), MINI (Mini International Neuro psychiatric Interview), SCEDAT (Costa del Sol Eating Disorder Access Project).

[a] BITE German translated version: Schmidt UH, Treasure JL. Overcoming Bulimia: A Self-Help Program. Translated by Thiels C. Weinheim: Beltz, 2000; Thiels C, Garthe R. Prevalence of Eating Disorders Among Students. Nervenarzt 2000; 71: 552-558.

[b] Validated French version of SCOFF: Garcia FD, Grigioni S, Allais E, Houy-Durand E, Thibaut F, Dechelotte P. Detection of eating disorders in patients: validity and reliability of the French version of the SCOFF questionnaire. Clin Nutr 2011; 30(2):178e81; Garcia FD, Grigioni S, Chelali S, Meyrignac G, Thibaut F, Dechelotte P. Validation of the French version of SCOFF questionnaire for screening of eating disorders among adults. World J Biol Psychiatry 2010; 11(7):888e93

[c] Original version validated in French of the ADO-BED: Carrard I, Kruseman M, Di Capua D, Suringar V, Chamay Weber C. Dépistage de l'hyperphagie boulimique chez les adolescents : validité faciale d'un questionnaire développé en français [Validity of a French screening questionnaire for binge eating disorders in adolescents]. Arch Pediatr. 2013 Oct; 20(10):1105-12. French. doi: 10.1016/j.arcped.2013.06.033. Epub 2013 Aug 8. PMID: 23932877.

[d] Own calculation.

[e] Results were collected only from the primary care setting.

[f] Original validated version of the SCOFF: Luck AJ, Morgan JF, Reid F et al. The SCOFF questionnaire and clinical interview for eating disorders in general practice: comparative study. BMJ 2002; 325: 755–756.; Morgan J, Reid F, Lacey JH. The SCOFF questionnaire: assessment of a new screening tool for eating disorders. BMJ 1999; 319: 1467–146

[g] Validated original version of the QEWPR-R: Spitzer, R. L., Yanovski, S. Z., & Marcus, M. D. (1993). The questionnaire on eating and weight patterns-revised (QEWP-R). State Psychiatric Institute: New York, New York

[h] Original validated version of EDE-Q: Fairburn CG, Cooper Z. The eating disorder examination. In: Fairburn CG, Wilson GT, editors. Binge eating: nature, assessment, and treatment. 12th edition New York: Guilford Press; 1993. p. 317–60; Fairburn, C. G., & Beglin, S. J. (1994). The assessment of eating disorders: Interview or self-report questionnaire? International Journal of Eating Disorders, 16, 363–370

[i] Study with two publications (articles)

[J] Validated original version of NEQ: Allison, K. C., Engel, S. G., Crosby, R. D., de Zwaan, M., O'Reardon, J. P., Wonderlich, S. A., et al. (2008). Evaluation of diagnostic criteria for night eating syndrome using item response theory analysis. Eat Behav, 9, 398e407.

[K] Original validated version of BEDS-7: Herman BK, Deal LS, DiBenedetti DB, Nelson L, Fehnel SE, Brown TM. Development of the 7-Item Binge-Eating Disorder Screener (BEDS-7). Prim Care Companion CNS Disord. April 28, 2016; 18(2):10.4088/PCC.15m01896. doi: 10.4088/PCC.15m01896. PMID: 27486542; PMCID: PMC4956427.

[l] Validated Arabic version of SCOFF: AOUN, A. et al. Validation of the Arabic version of the SCOFF questionnaire for the screening of eating disorders. Eastern Mediterranean Health Journal, v. 21, n. 5, 2015;

[m] Validated version of EDE-Q 6.0: Fairburn C, Cooper Z, O'Connor M (2008). Cognitive behavior therapy and eating disorders. Appendix A: Eating disorder examination, pp. 268–269. The Guilford Press: New York.

[n] Original validated English version of EAT-26: Garner, D. M., Olmsted, M. P., Bohr. Y. & Garfinkel, P. E. (1982). The Eating Attitudes Test: psychometric features and clinical correlates. Psychological Medicine 12, 871-878.

[o] EDQ Screen tool: Stice E, Telch CF and Rizvi SL (2000) Development and validation of the Eating Disorder Diagnostic Scale: A brief self-report measure of anorexia, bulimia, and binge-eating disorder. Psychological Assessment 12(2): 123–131.

[p] Validated original version of EDS-PC: COTTON, M.A.; BALL, C.; ROBINSON, P. Four simple questions can help screen for eating disorders. Journal of general internal medicine, v.18, no. 1, p: 53–56, 2003

[q] Binge Eating Disorder Screening Tool (modified from the Eating Disorders Screen for Primary Care and SCOFF screening tools - Cotton, 2003 and from the full article: "Taking Back Control in Binge Eating Disorder" by Sara Weekly, MD The Carlat Child Psychiatry Report, Volume 7, Number 4, May/June 2016)

[r] Short version of EDE-Q: Mond, J. M., Hay, P. J., Rodgers, B., & Owen, C. (2006b). Eating Disorder Examination Questionnaire (EDE-Q): Norms for young adult women. Behavior Research and Therapy, 44, 53–62

[s] Validated version of EDE-Q 6.0 : Fairburn C, Cooper Z, O'Connor M (2008). Cognitive behavior therapy and eating disorders. Appendix A: Eating disorder examination, pp. 268–269. The Guilford Press: New York.

[t] Validated and adapted version of the EDE-Q in Portuguese: Machado, P. P. P. (2007). Eating Questionnaire: EDE-Q5.2. Psychology Research Centre. Braga: University of Minho; Machado BF, Machado PP, Klein J, Gonc ̧alves S. The Eating Disorder Examination Questionnaire: Reliability and Norms for Portuguese Adolescent Girls. Barcelona, 2006

[u] Study with two publications (articles)

[v] Validated Spanish version of SCOFF: Garcia-Campayo J, Sanz-Carrillo C, Ibañez JA, et al. Validation of the Spanish version of the SCOFF questionnaire for the screening of eating disorders in primary care. J Psychosom Res 2005; 59:51–55

[w] Validated Spanish version of the EAT: Gayou-Esteva U, Ribeiro-Toral R. Eating disorders identification of risk cases among students from Querétaro. Rev Mex Trastor Aliment. 2014; 5:115--23.

[x] Validated Spanish version of EDE-Q: Peláez-Fernández MA, Javier Labrador F, Raich RM. Validation of eating disorder examination questionnaire (EDE-Q)– Spanish version–for screening eating disorders. Span J Psychol. 2012; 15:817--24

[y] Validated Arabic version of NEQ: Elsadek AM, Hamid MS, Allison KC. Psychometric characteristics of the night eating question naire in a Middle East population. Int J Eat Disord. 2014; 47(6):660–5. doi: 10. 1002/eat.22285.

[z] Original validated version of the SDE: Maguen, S., et al. Screen for Disordered Eating: Improving the accuracy of eating disorder screening in primary care. General hospital psychiatry, v. 50, p: 20–25, 2018
