# Supplementary material for: Validated strategies for screening for eating disorders in primary health care: A scoping review with a focus on adolescents and adults
Source: PLoS One. 2026 Aug 3;21(8):e0347184. doi: 10.1371/journal.pone.0347184 (PMC13432121; doi:10.1371/journal.pone.0347184)
Supplement: S2 Table — (PDF) [file pone.0347184.s005.pdf]

## S2 Table. Characteristics of the nonempirical studies included in the review (n=21).

| Author, Year (Magazine) | Study design | Ain | Main results for the research questions |
|---|---|---|---|
| Bryant et al 2022 (Journal of Eating Disorders) | Systematic review | Identify and summarize population screening approaches and evidence relevant to clinical assessment and ED diagnosis to date, with a focus on Western health systems that can inform health policy and translational research in the Australian context. | Evidence highlights the importance of ED screening in PHC, which is essential for early identification. However, current tools may fail to detect all DSM-5 EDs, due to population diversity and symptom overlap. Risk groups include individuals with rapid weight gain, college students, and people with type I diabetes. The review points to gaps in screening and diagnosis, reinforcing the need for research to improve early detection, reduce barriers, and improve access to care, especially for vulnerable populations. |
| Buchholz et al., 2017 (Journal of Clinical Psychology in Medical Settings) | Narrative review | Provide a brief overview of ED and practical guidance for psychologists working in integrated primary care settings to facilitate the identification and management of these conditions. | The review presents an ED screening algorithm in PHC for psychologists and specific screening strategies for physicians, nutritionists, and dietitians. Physicians should investigate patients with abnormal electrolyte levels or cardiac symptoms, while dietitians and dietitians should evaluate eating habits and identify binge eating or compensatory behaviors in cases of unexplained weight loss or gain. Screening should use validated instruments, including: SCOFF (5 items, <1 min, detects AN and BN, high sensitivity/specificity: 0.85/0.90, but false positives of 12.5%; cutoff point: 2; less valid for individuals aged 30-40 years and with a BMI ≥ 27.5); QEWP-5 (19 items, 5 min, detects BED, sensitivity/specificity: 0.74/0.35); EDE-Q (34 items, 5 min, sensitivity/specificity according to TA - AN: 0.80/0.98; BN: 0.79/0.92; BED: 0.37/0.95, cutoff point: 2.7; best for young women with normal weight, brief version of 12 items available); EDDS (22 items, potential use in PHC, but more research is needed). The review highlights the importance of strategies adapted to different professionals and the need for further studies to optimize screening in PHC. |
| Cadwallader et al., 2016 (Eating and weight disorders) | Systematic review | To describe the effect (clinical outcomes and care trajectory) of ED screening among patients in primary care settings. | The review points out as screening methods self-administered questionnaires, such as EAT-26 for AN, BULIT-R and EDE-RQ4 for BN, in addition to telephone interviews based on the DSM-IV. After screening, there was an increase in the frequency of general consultations, referrals to psychiatry and prescriptions of antidepressants, especially among elderly individuals. |
| Chew et al., 2022 (Journal of Pediatric Health Care) | Narrative review | Describe the etiology and risk factors for the most common EDs; indicate the diagnostic criteria for AN, BN, and periodic BED according to the Diagnostic and Statistical Manual of Mental Disorders, 5th Edition (DSM-5); and outline the ED screening process. | Primary care providers play an important role in the early detection of ED, using screenings during annual or physical exams. Tools such as SCOFF and EDE-Q are valid and appropriate for screening, while ADO-BED is used for adolescents with obesity. Positive responses indicate the need for further evaluation. Available resources include *the Eating Attitudes Test, Eating Disorder Inventory-3,* and organizations such as *the Academy for Eating Disorders.* |
| Feltner et al., 2022 (JAMA) | Systematic review | To review the evidence on screening for ED in adolescents and adults to inform the US *Preventive Services Task Force.* | Five studies evaluated the accuracy of screening tests in primary care: VA-BES (1 study), SCOFF (4 studies), EDS-PC (2 studies) and SDE (1 study). The SCOFF, for cutoff point 02, showed a sensitivity of 84% and specificity of 80% in adults, and sensitivity of 73% and specificity of 78% in adolescents. The EDS-PC had a sensitivity of 97-100% and a specificity of 40-71%. The VA-BES had a sensitivity of 89% and a specificity of 69%. EDS had a sensitivity of 91% and specificity of 58%. While useful, the tests have limitations due to the number of studies, and potential harms include false positives that |

| | | | |
|---|---|---|---|
| | | | lead to unnecessary referrals |
| Hay, Hart, Wade, 2022 (International Journal of Eating Disorder) | Narrative Review/Forum | This forum presents the current state of research on screening and identification of people with ED in the community and in primary care, taking a long-term perspective that highlights the slow progress in instrument development and the impact on policy and practice. | Tools such as SCOFF are useful but insufficient to detect all ED in primary care. Barriers to screening include limited consultation time, stigma, low training of mental health professionals, and discomfort in treating these cases. Screening may increase consultations and referrals, but there is no clear evidence of improvement in clinical outcomes. It is essential to ensure appropriate treatment after referral. Discussion about food should be encouraged, timely treatment ensured, and access to resources and specialists increased. Education should combat stigma and prejudice associated with ED. The Guidelines recommend attention to adolescents, but many cases are neglected. Screening can be biased toward cultural stereotypes, ignoring other vulnerable populations. Online resources can improve results. Programs like *Mental Health First Aid* help reduce stigma. The inclusion of ED in formal mental health screening in primary care is a promising approach. |
| Kalindjian et al., 2021 (Eating and Weight Disorders) | Scoping Review | Analyze the current scientific data on the early detection of ED, which is one of the facets of secondary prevention. | General practitioners are essential in the early detection of ED, since patients with ED attend appointments more often. However, the detection rate, especially of BN, is lower than expected, due to stigma. Factors that increase detection include young physicians, women, contraceptive prescription, and nearby specialized centers. Primary care dentists and gynecologists also play an important role, but their knowledge of ED is variable. *E-learning* can improve the confidence of these professionals in the management of ED. |
| Kornstein et al., 2016 (The Primary Care Companion for CNS Disorders) | Narrative review | Review the clinical skills needed to recognize, diagnose, and manage ED in primary care. | Primary care professionals often involved in weight management are well positioned to identify BED, especially in patients with diabetes. Tools such as EDE-Q and QEWP-R can help identify BED. Short instruments, such as the EDS-PC, BEDS-7, and EDA-5, are useful in primary care, and the BEDS-7 is specific for BED. However, the time constraints of professionals can make it difficult to implement these tools. Specialists must develop more efficient tools for these professionals. |
| Kornstein, 2017 (The Journal of Clinical Psychiatry) | Narrative review | This review addresses general aspects related to ED, such as diagnostic criteria, epidemiology, burden of disease, underdiagnosis, undertreatment, and barriers to the recognition of EDs | Screening for BED in PHC faces challenges, such as underdiagnosis in male patients, since ED is more common in women. To overcome these barriers, it is essential to use screening tools, as many patients do not talk openly about their eating habits. Screening should be considered for patients who are overweight, obese, have mood or anxiety disorders, substance abuse, and impulse control problems. Some patient-administered screening tools include: the 16-item BES, the 7-item BEDS-7, the 28-item EDE-Q, the 18-item ELOCS, and the 26-item QEWP-5. The SCOFF with 5 items can be administered by the clinician or patient and the tools administered by the clinician: the EDE with 28 items, the ESP with 5 items, the EDA-5 with 5 items, the and the YBOCS-BE with 10 items. The ideal tool is brief, easy to administer, and based on self-reporting. A positive score indicates the need for further evaluation |
| Kutz et al., 2020 (Journal of General Internal Medicine) | Systematic review | To evaluate the diagnostic accuracy of the SCOFF (*Sick, Control, One, Fat, Food) questionnaire* for TA according to the DSM-5 in the general population. | All validation studies in primary care were included in this review. In subgroup analyses, 12 studies showed that SCOFF had a pooled sensitivity of 0.90 and specificity of 0.86 in medical settings, including PHC. Another 12 studies with adults revealed a sensitivity of 0.84 and a specificity of 0.86, while in children, adolescents and young adults, the sensitivity was 0.86 and a specificity of 0.79. Accuracy was higher in case–control studies with interviews as the reference standard and a higher percentage of women. Although sensitive for young women at risk for AN and BN, SCOFF psychometrics are unknown for all DSM-5 ED diagnoses and diverse populations. There is insufficient evidence to recommend SCOFF for large-scale screening. Meta-analysis suggests that it is useful for young women, but a new tool needs to be developed for the full range of DSM-5 TA in heterogeneous samples. |

| | | | |
|---|---|---|---|
| Lemly et al., 2022 (Primary care companion for central nervous system disorders) | Narrative review | This review includes updated screening and treatment guidelines, as well as relevant studies on the care of adult patients with ED, providing concise guidance for primary care physicians on the outpatient management of these patients | Primary care professionals are most often on the front lines of ED screening and detection. Short tools such as SCOFF and EAT-26 are used to assess ED, with SCOFF being highly scalable and ideal for screening at annual visits or for patients with worrying signs. The EAT-26, although longer, offers detailed information. However, there is a lack of preparation among primary care physicians to identify risk behaviors or clinical complications related to ED. |
| Martin & Ammerman, 2002 (The Nursing clinics of North America) | Narrative review | The focus of this article is on the screening, identification, and evaluation of ED in adolescents in primary care, as well as the approach to treatment and follow-up of the adolescent at risk or diagnosed with an eating disorder. | Adolescent primary care providers often use evidence-based screening forms, such as *Bright Futures* or GAPS, which recommend annual screening for ED. They may also develop their own forms with appropriate questions, which are especially important for the evaluation of BN, as many patients will be normal weight or overweight. |
| Mills, Hyam, Schmidt, 2023 (Adolescent Health, Medicine and Therapeutics) | Narrative review | To synthesize the literature on barriers and facilitators to early intervention in ED, considering factors related to the patient, the clinician, the services and the health system. | Primary care is essential for the early detection, initial management, and referral of ED. Despite their importance, primary care physicians often face challenges due to limited clinical training on ED, which leaves them unprepared to diagnose and manage these disorders. A lack of empathy and connection can result in negative experiences for patients, while empathetic professionals can make it easier for them to seek help. The presence of comorbidities and atypical forms, such as atypical anorexia and ARFID, can make identification difficult, aggravated by stereotypes such as the "SWAG" profile (thin, white, upper-class, and young). Digital tools, such as SCOFF and IOI-S, help with tracking and routing. Specific training for general practitioners is being developed to fill gaps and improve the early identification of ED. |
| Montano et al., 2016 (Postgraduate Medicine) | Narrative review | Highlight ways in which clinicians can identify individuals who may have ED, and emphasize the need for short, validated tools to aid in diagnosis, enabling appropriate treatment. | Due to the limited time in PHC, scales help in the diagnosis of ED. The EDE-Q and the QEWPR correlate well with the EDE in the identification of eating disorders in obese individuals, suggesting that they are accessible alternatives in PHC. Tools such as EDA-5, EDS-PC, and BEDS-7 can also be useful. Including questions about weight gain history may help, as obese individuals with eating eating have more weight cycling. |
| Parpia, Spettigue, Norris 2023 (Canadian Family Physician) | Narrative review | To address the screening, diagnosis, and treatment of adolescents with AN and atypical AN in primary care. | Primary care providers are ideal for identifying adolescents at risk for ED and initiating treatment. The SCOFF questionnaire (5 items) has high sensitivity for AN. The ODES-Y (2 items) is accurate to detect disordered eating behaviors, and a positive result in young people, where a positive result (yes for both questions) on screening should lead to more questions about intake, concern about weight and shape, and eating disorder symptoms. |
| Pritts & Susman, 2003 (American Family Physician) | Narrative review | This review focuses on the recognition and diagnosis of ED in primary care. | Patients at high risk for ED should be screened routinely. The SCOFF questionnaire is a promising tool. Questions such as "How many diets have you gone on in the last year?", "Do you think you should go on a diet?", "Are you dissatisfied with your body?" and "Does your weight affect your self-image?" can also be helpful, requiring further investigation if there are positive answers. |
| Sangvai, 2016 (Primary care clinics in office practice) | Narrative review | This article focuses on outpatient diagnosis and management of ED in adolescent and adult populations. | Brief tools such as SCOFF or ESP are more practical in primary care. Due to the low sensitivity of the SCOFF (53.7%), it should be complemented with questions about family history of ED, affective disorders, or activities that promote thinness. ESP, with 5 short questions, detects ED; two or more abnormal responses indicate positive screening, requiring further evaluation for diagnosis according to the DSM-5. |

| | | | |
|---|---|---|---|
| Saules et al., 2015 (Journal of clinical outcomes management) | Narrative review | Describe the epidemiology, clinical features, clinical course, medical complications, and treatment of binge eating disorder. | The EDE-Q may underestimate the frequency of binge eating episodes and overestimate the severity of the pathology. The QEWP-R has been revised to reflect DSM-5 criteria. BES is considered efficient. EDDS does not evaluate in an equivalent manner on African American and Caucasian clients. One study indicated readability and comprehension problems with most measures of BED. |
| Scrandis, 2023 (The Nurse Practitioner) | Narrative review | This review covers topics related to eating disorders, such as neurobiology, risk factors, assessment (including screening), and treatment | Nurses can use questions based on the TCA criteria to assess binge eating patterns, such as eating without hunger or in secret, with negative feelings about eating. In case there are concerns, additional tracking options are available. The widely used SCOFF is effective for identifying eating symptoms, but it has limitations outside of AN and BN. BEDS-7, with 100% sensitivity and 39% specificity, is specific for BED. The APA recommends supplementing the SCOFF with the first BEDS-7 question about episodes of overeating in the past 3 months. |
| Sim et al., 2010 (Mayo Clinic proceedings) | Narrative review | This review addresses practical issues faced by primary care physicians in managing these conditions and other central aspects in the care of complex patients with medical and psychiatric comorbidities. | The review suggests screening tests for ED in primary care. For AN and BN, SCOFF or EDDS is recommended, and SCOFF is brief and with high sensitivity. For the diagnosis, two or more affirmative items warrant investigation, especially if there is substantial weight loss or self-induced vomiting. For BED, the useful EAT, EDDS, and QEWP-R are indicated in community and clinical samples. There is no recommendation for screening for NEQ. Questions about body and weight should accompany the primary care professional interview. |
| Walter, Anna, 2019 (Pharmacology onLine) | Narrative review | The role of the primary care physician in the diagnosis and treatment of ED ranges from health promotion and prevention (primary, secondary and tertiary), through early diagnosis with the identification of new cases, assessment of severity, treatment of mild and moderate cases, referral to a specialist, management of possible complications and follow-up. | Universal screening is not recommended due to low prevalence, but it would be useful to collect information about diet and body in the initial history. If ED is suspected, the primary care physician should administer screening questionnaires. The use of a self-administered questionnaire can be seen as a barrier to the therapeutic relationship. Instead, the doctor should know the questionnaires to ask the right questions during the consultation. SCOFF, created in the United Kingdom to raise suspicions of ED, has been validated only in women in primary care, and further studies are needed before its use. |

Acronyms: ADO-BED (Binge Eating Disorder in Adolescents), AN (Anorexia Nervosa), APA (American Psychiatric Association), BED (Binge Eating Disorder), BES (Binge Eating Scale), BEDS-7 (7-Item Questionnaire for Binge Eating Disorder), BN (Bulimia Nervosa), BULIT-R (Revised Bulimia Test), DSM-5 (Diagnostic and Statistical Manual of Mental Disorders, 5th Edition), EDA-5 (Eating Disorders Assessment for the DSM-5), TA (Eating Disorder), EDDS (Eating Disorders Diagnostic Scale), EDE (Eating Disorder Exam), EDE-Q (Eating Disorder Exam Questionnaire), EDE-RQ4 (Eating Disorder Exam, 4 Questions), EAT-26 (Eating Attitudes Test), ELOCS (Loss of Eating Control Scale), ESP (Eating Disorders Screening Questionnaire in Primary Care), GAPS (Guidelines for Preventive Services for Adolescents), IOI-S (InsideOut Institute Questionnaire), NEQ (Night Eating Questionnaire), ODES-Y (Ottawa Disordered Eating Questionnaire for Youth), PHC (Primary Health Care), QEWP-5 (Dietary and Weight Patterns Questionnaire – 5 items), QEWP-R (Eating and Weight Patterns Questionnaire – Revised), YBOCS-BE (Yale-Brown Obsessive-Compulsive Disorder Scale Modified to Binge Eating).
