# Supplementary material for: Validated strategies for screening for eating disorders in primary health care: A scoping review with a focus on adolescents and adults
Source: PLoS One. 2026 Aug 3;21(8):e0347184. doi: 10.1371/journal.pone.0347184 (PMC13432121; doi:10.1371/journal.pone.0347184)
Supplement: Attachment — Submitted filename: Peer Review PLoS One.pdf [file pone.0347184.s007.pdf]

To the authors,

This review is sure to be helpful to ED researchers, medical educators, and practicing health professionals. I was not a first-round reviewer but appreciate your edits in response to Reviewer 1. My recommendation is for a minor revision, primarily focused on consistency and clarity in how descriptions of the studies are framed throughout the paper, as it is difficult to track within the scope of how many total studies were included, once in the thick of the paper.

Specific examples of where clarity can be improved:

"Among the 32 studies that included PHC professionals, sixteen predominantly involved physicians, while other health professional categories were underrepresented. For more details on demographic and socioeconomic characteristics, see Table S1. Forty-five studies were conducted exclusively in PHC, nine studies in environments generalizable to PHC, such as university-affiliated services and hospital-affiliated services, and two combined both contexts. Of these, 31 studies were conducted in urban and metropolitan areas." - **Needs better contextualization within the total number of studies included, rather than the number of studies that included professionals within the total number of studies.**

General language example: Of the X total of studies that met inclusion criteria, Y mentioned ____. Of these, ___ percent or ___ number had these traits.

Table 4 - Table 4 - Main barriers and facilitators to screening reported by professionals – **Can enhance clarity by nesting frequency within total number of relevant articles.**

Line 228: "Four studies revealed that the majority of users were married and had low to intermediate family income (monthly income between $1000 and $2600 USD) whereas three studies reported that the most participants had nine or more years of education"

Minor edits:

Line 98 "What are the characteristics of the validated strategies for at screening and their application…" - **The language "for at" is unclear and potentially a typo.**

Make sure all "eating disorders" are abbreviated to ED (e.g., line 393)

Line 327 - "Female professionals showed a greater tendency to adopt the screening questionnaire…" **Can add existing citations that this is consistent with literature showing that males are less likely to screen.**

Conceptual Explanations:

While I can infer as a reviewer, please provide a rationale for excluding ARFID.

Other Comments:

Lines 401 to 408 can use another review and tighter editing.

Line 401: “Even so, these tools have limitations in terms of diagnostic accuracy, <u>being subject to false positives and, therefore, generating unnecessary referrals</u>” - **This feels a bit narrow and not robustly supported. I recommend rephrasing to “being subject to both false positives and missed capture” if keeping.**

“In addition, screening can influence the trajectory of care, increasing the frequency of consultations and referrals without robust evidence that this practice effectively improves clinical outcome” - **This feels dangerous to include and not robustly supported. Literature points to the importance of early intervention within first three years of illness onset as instrumental to positive clinical outcomes.**

The above two statements and the section in general needs to be conceptually merged with this stronger point “Additionally, another critical point concerns the cultural biases present in screening instruments, which tend to privilege cultural stereotypes of gender, race, and social class, for example, the profile of white women from the middle to upper classes, as opposed to the neglect of populations with greater vulnerability, such as those in situations of food insecurity [106].”
