# Supplementary material for: Validated strategies for screening for eating disorders in primary health care: A scoping review with a focus on adolescents and adults
Source: PLoS One. 2026 Aug 3;21(8):e0347184. doi: 10.1371/journal.pone.0347184 (PMC13432121; doi:10.1371/journal.pone.0347184)
Supplement: Attachment — Submitted filename: Response_to_Reviwes_auresp_2.docx [file pone.0347184.s009.docx]

**Response to Reviewers – Manuscript PONE-D-25-28582**

Dear Editors and Reviewer,

We would like to thank you for the careful evaluation of our manuscript entitled *“Validated strategies for screening for eating disorders in primary health care: a scoping review with a focus on adolescents and adults”* (Manuscript ID: PONE-D-25-28582). We appreciate the constructive and detailed comments, which substantially contributed to improving the clarity, transparency, and overall quality of the manuscript.

We carefully addressed all comments raised by the reviewer and fulfilled the journal requirements. All changes are highlighted in the revised manuscript using the track changes feature. Below, we present a detailed point-by-point response.

**Responses to Reviewer #1**

We thank the reviewer for the positive evaluation of the manuscript and for the valuable suggestions. All comments were carefully analyzed and addressed as follows:

**Comment 1:** Line 154: The text currently refers to “a fifth reviewer.” Should this instead be “a third reviewer”? Please clarify the intended number of reviewers.

**Response:** As suggested, the term “a fifth reviewer” was corrected to “a third reviewer” to accurately reflect the review process.

**L153-154:** *Any disagreement between each pair was resolved through discussion or, if necessary, with the mediation of a third reviewer.*

**Comment 2:** Use of Covidence: There is no need to write out the full name of Covidence. The short form “Covidence” is sufficient throughout.

**Response:** As suggested, the manuscript was revised and the platform is now referred to only as “Covidence” throughout the text.

**Comment 3:** Percentages and Study Counts: For clarity, please present percentages and the corresponding number of studies within the same parentheses (e.g., “25% (n = X)”).

**Response:** As suggested, the presentation of percentages and study counts was revised throughout the manuscript to improve clarity and readability while avoiding excessive use of parentheses.

**Comment 4:** Line 250: Please expand the acronym VA‑BES at first use for clarity.

**Response:** As requested, the acronym VA-BES was expanded at first mention in the manuscript.
*L267-247: The number of items in the instruments ranged from one item in the Veterans Affairs Binge Eating Screening (VA-BES) (...)*

**Comment 5:** Table 3: The value PPV = 5 for the SCOFF questionnaire appears very low. Please verify this value and correct it if necessary.

**Response:** Thank you for identifying this issue. The value was reviewed and corrected in Table 3.

**Commet 6:** Line 300 and similar headings: Please adjust the formatting of this heading (and other similar headings) to clearly distinguish it from regular body text.

**Response:** As suggested, the formatting of this heading and similar headings throughout the manuscript was revised to improve distinction from the main body text.

L300: *“3.3.1.3 Feasibility of screening (RQ4)”*

**Comment 7:** Table 4: The table formatting needs revision—several horizontal lines appear to be missing. Please ensure consistent and complete table borders.

**Response:** As requested, Table 4 formatting was revised and the horizontal borders were standardized to ensure consistency throughout the table. Additionally, the table was reorganized to present barriers and facilitators according to the prevalence of studies reporting them, improving clarity and data presentation.

**Comment 8:** Line 348: Please move reference [73] so that it appears at the end of the sentence.

**Response:** As suggested, reference [73] was moved to the end of the sentence.

L346-347: *(...) and the existence of a multidisciplinary team for treatment, which were considered potent facilitators in one study (Table 4 and Table S1) [73].*

**Comment 9:** Line 353: Please replace “and one was scoping” with “one was a scoping review.”

Response: As suggested, the sentence was revised for grammatical accuracy and clarity.

L352: *(...) one was a scoping review.*

**Responses to Reviewer #2**

We thank the reviewer for the positive evaluation of the manuscript and for the valuable suggestions. All comments were carefully analyzed and addressed as follows:

Reviewer #2:

We thank the reviewer for the careful reading of the manuscript and for the constructive suggestions. We appreciate the recognition of the relevance of this review to ED researchers, medical educators, and health professionals. All comments were carefully considered, and the manuscript was revised accordingly.

**Comment 1:** This review is sure to be helpful to ED researchers, medical educators, and practicing health professionals. I was not a first-round reviewer but appreciate your edits in response to Reviewer 1. My recommendation is for a minor revision, primarily focused on consistency and clarity in how descriptions of the studies are framed throughout the paper, as it is difficult to track within the scope of how many total studies were included, once in the thick of the paper. “Among the 32 studies that included PHC professionals...” - Needs better contextualization within the total number of studies included, rather than the number of studies that included professionals within the total number of studies.

General language example: Of the X total of studies that met inclusion criteria, Y mentioned ____. Of these, ___ percent or ___ number had these traits.
**Response:** We thank the reviewer for this important observation. The manuscript was revised throughout to improve consistency, clarity, and readability in the presentation of study characteristics and findings. The Results section was standardized to better contextualize subgroup analyses, frequencies, and proportions within the total number of included studies whenever appropriate.

*Examples:*

*L227-228: On the studies including PHC users, four revealed that the majority of users were married [49,50,52,54].”*

*L231-232: Of the studies included, 32 involved PHC professionals. Among these, 16 studies predominantly involved physicians, while other health professional categories were underrepresented [58,63,69,72,74–81,83–86].*

*L234-237: Regarding study settings, most studies (n=45 were conducted exclusively in PHC, while nine studies in environments generalizable to PHC, such as university-affiliated services and hospital-affiliated services, and two combined both contexts. In terms of location, 31 studies were conducted in urban and metropolitan areas (Table S1).*

**Comment 2:** Table 4 - Main barriers and facilitators to screening reported by professionals – Can enhance clarity by nesting frequency within total number of relevant articles.
**Response:** As suggested, Table 4 was revised to improve clarity by contextualizing the frequency of barriers and facilitators within the total number of relevant studies. Additionally, the table was reorganized according to the prevalence of studies reporting each barrier and facilitator.

**Comment 3**: Line 228: “Four studies revealed that the majority of users were married...”
Response: As suggested, the sentence was revised to improve clarity and readability regarding the sociodemographic characteristics reported across studies.

*L227-229: (…) On the studies including PHC users, four revealed that the majority of users were married [49,50,52,54].*

**Comment 4:** Line 98 “What are the characteristics of the validated strategies for at screening and their application...” - The language “for at” is unclear and potentially a typo.
**Response:** Thank you for identifying this issue. The typographical error was corrected in the revised manuscript.

*L96-97: What are the characteristics of the validated strategies for ED screening and their application…*

**Comment 5**: Make sure all “eating disorders” are abbreviated to ED (e.g., line 393).
**Response:** As suggested, the manuscript was revised to ensure consistency in the use of the abbreviation “ED” throughout the text.

**Comment 6:** Line 327 - “Female professionals showed a greater tendency to adopt the screening questionnaire...” Can add existing citations that this is consistent with literature showing that males are less likely to screen.
**Response:** As suggested, the sentence in the Results section was revised for greater clarity.
*L327-329: In one study, female professionals showed a greater tendency to adopt the screening questionnaire to the detriment of the evaluation of clinical signs [80].*

Additionally, literature was incorporated into the Discussion to contextualize this finding. A study on substance use screening during pregnancy was included, showing that female obstetricians/gynecologists were more likely to believe in the effectiveness of screening and to adopt standardized screening tools in clinical practice [111].
*L435-442: Gender differences in attitudes toward the use of screening questionnaires were observed in the study by Boulé McSherry (2002). The findings suggest that female professionals were more likely than their male counterparts to adopt screening strategies based on standardized instruments [80]. However, the factors underlying these differences remain unclear. Similar findings have been reported in other areas of mental health-related screening. A study on substance use screening during pregnancy found that female obstetricians/gynecologists were more likely to believe in the effectiveness of screening and to adopt standardized screening tools in clinical practice [111].*

**Comment 7:** While I can infer as a reviewer, please provide a rationale for excluding ARFID.
**Response:** Thank you for this important observation. A clarification regarding the exclusion of ARFID was added to the manuscript. ARFID was excluded from this review because the preliminary search did not identify studies evaluating validated screening strategies for ARFID in adolescents and adults within the scope of primary health care. As the objective of this review was to map existing screening strategies applicable to primary health care, only eating disorders for which screening approaches had been identified in the literature were included.

**Comment 8:** Lines 401 to 408 can use another review and tighter editing.

**Comment 9:** Line 401: “Even so, these tools have limitations in terms of diagnostic accuracy...” - I recommend rephrasing to “being subject to both false positives and missed capture” if keeping.

**Response: As** suggested, the paragraph was revised to acknowledge both false positives and missed cases, improving conceptual accuracy and balance in the discussion.
*L400-402: Even so, these tools have limitations in terms of diagnostic accuracy, being subject to false positives and missed cases [21]. While false positives may lead to unnecessary referrals, false negatives may delay identification and timely access to care.*

**Comment 10:** “In addition, screening can influence the trajectory of care...” - This feels dangerous to include and not robustly supported. Literature points to the importance of early intervention within first three years of illness onset as instrumental to positive clinical outcomes.

**Response:** Thank you for this valuable observation. We agree that the statement regarding the influence of screening on the trajectory of care was not sufficiently supported by the available evidence and could be interpreted beyond the scope of the findings discussed in this review. Therefore, we have removed this statement from the manuscript

**Comment 11:** The above two statements and the section in general needs to be conceptually merged with this stronger point regarding cultural biases in screening instruments.
**Response:** As suggested, the section was revised to improve conceptual integration between the limitations of screening instruments and cultural biases that may affect the identification of underrepresented populations.

*L402-406: Additionally, another critical issue concerns the cultural biases present in screening instruments, which tend to privilege cultural stereotypes of gender, race, and social class, for example, the profile of white women from the middle and upper class background while a neglecting populations experiencing greater social vulnerability, such as individuals in situations of food insecurity [106].*

**Additional note:** We identified an inconsistency in the reference list and corrected it in the revised manuscript. Reference 110 was inadvertently omitted, and the reference previously numbered as 110 corresponded to reference 111. In addition, a new reference (reference 112) was included in response to the reviewers’ suggestion to incorporate additional relevant studies in the Discussion section. The numbering has been updated accordingly throughout the manuscript.

**We sincerely thank the reviewer and the editors for their valuable feedback. We believe that these revisions have substantially strengthened the manuscript and hope that it is now suitable for publication.**

**Sincerely,**

**Lisane da Silva Oliveira**
