# Supplementary material for: Validated strategies for screening for eating disorders in primary health care: A scoping review with a focus on adolescents and adults
Source: PLoS One. 2026 Aug 3;21(8):e0347184. doi: 10.1371/journal.pone.0347184 (PMC13432121; doi:10.1371/journal.pone.0347184)
Supplement: Attachment — Submitted filename: Response to Reviwes.docx [file pone.0347184.s008.docx]

**Response to Reviewers – Manuscript PONE-D-25-28582**

Dear Editors and Reviewer,

We would like to thank you for the careful evaluation of our manuscript entitled *“Validated strategies for screening for eating disorders in primary health care: a scoping review with a focus on adolescents and adults”* (Manuscript ID: PONE-D-25-28582). We appreciate the constructive and detailed comments, which substantially contributed to improving the clarity, transparency, and overall quality of the manuscript.

We carefully addressed all comments raised by the reviewer and fulfilled the journal requirements. All changes are highlighted in the revised manuscript using the track changes feature. Below, we present a detailed point-by-point response.

**Responses to Journal Requirements**

**Requirement 1 – PLOS ONE style and formatting**

The manuscript was thoroughly reviewed to comply with the PLOS ONE formatting and style guidelines.

**Requirement 2 – Role of the funders**

We confirm that the funders had no role in study design, data collection and analysis, decision to publish, or preparation of the manuscript. The following statement was included in the cover letter, as requested:

*“The funders had no role in study design, data collection and analysis, decision to publish, or preparation of the manuscript.”*

**Requirement 3 – Funding information in the Acknowledgments**

All funding-related information was removed from the Acknowledgments section. The Funding Statement was maintained exclusively in the appropriate section of the online submission system, as requested.

L624-626: *“The authors acknowledge the support provided by the Coordination for the Improvement of Higher Education Personnel (CAPES), Brazil, Code 001. We would also like to thank librarian Gesner Francisco Xavier for his assistance in the development of the preliminary search strategy.”*

**Requirement 4 – Copyright issues related to Figure 3**

Figure 3 was removed and replaced with a new figure developed by the authors using public domain data, fully compatible with the CC BY 4.0 license. The figure caption was updated to comply with PLOS ONE attribution and licensing requirements (CC BY 4.0).

**Requirement 5 – Figure quality**

High-resolution versions of both figures (Fig 1 and Fig 2) were submitted in accordance with the PLOS ONE figure preparation guidelines. In response to the reviewer’s comments, the former Figure 1 was removed and its content was incorporated into a table. The former Figure 2 was resubmitted and renumbered as Figure 1. The former Figure 3 was replaced and renumbered as Figure 2. Figure 4 was retained and renumbered as Figure 3. Former Figures 5 and 6 were removed from the manuscript.

**Requirement 6 – Evaluation of citation recommendations**

The reviewer comments did not include explicit recommendations to cite specific previously published works. Nevertheless, the authors critically reviewed the relevant literature to ensure that the cited studies remain current, relevant, and aligned with the objectives of the review.

**Requirement 7 – Review of the reference list**

The reference list was carefully reviewed to ensure its completeness, accuracy, and currency. No retracted articles were identified among the cited references. The references remain consistent with the scope, objectives, and methodological rigor of the study.

**Responses to Reviewer #1**

We thank the reviewer for the positive evaluation of the manuscript and for the valuable suggestions. All comments were carefully analyzed and addressed as follows:

**Abstract**

**Comment 1:** Replace “investigate” with a more precise verb such as “provide an overview” or “map”.

**Response:** As suggested, the verb “investigate” was replaced with “map” to better reflect the objectives of a scoping review.

L33–35: *“This scoping review aimed to map the available evidence on validated strategies for screening for eating disorders in adolescents and adults in primary care.”*

Additionally, we clarified the aim of the review in the Introduction:

L93-94: *“The aim of this review is to map the extent of the available evidence on validated strategies for ED screening in adolescents and adults assisted by PHC.”*

**Comment 2:** Add the OSF registration number in the abstract.

**Response:** As requested, the OSF registration number was included in the abstract.

L35-37: “The study followed the Preferred Reporting Items for Systematic reviews and Meta-Analyses extension for Scoping Reviews, with a protocol registered in the Open Science Framework (doi: 10.17605/OSF.IO/498FU).”

**Comment 3:** Cite the six databases used.

**Response:** As suggested, the six databases were specified in the abstract:

L37-39: *“Searches were conducted in the databases MEDLINE, Embase, Latin American and Caribbean Health Sciences Literature, Web of Science, PsycINFO, and Cumulative Index to Nursing and Allied Health Literature.”*

**Comment 4:** Expand on the identified gaps to offer more insight.

**Response:** The abstract was revised as suggested to provide greater clarity and a more detailed description of the main evidence gaps identified. To comply with the 300-word limit, conciseness and rephrasing adjustments were made without loss of relevant content.

L50-55: *“Despite the potential of several instruments, robust evidence on their validity, reliability, and applicability remains limited, particularly in low- and middle-income settings and among underrepresented and adolescent populations. Structural and training barriers hinder the effective implementation of screening, highlighting the need for articulated actions between professionals, services, and public policies.”*

**Comment 5:** Ensure consistency between the abstract submitted to the system and the one included in the main manuscript.

**Response:** Full consistency between the abstract submitted to the system and the abstract included in the main manuscript was ensured.

**Introduction**

**Comment (Line 63):** Replace “indicate” with “estimate”. The Global Burden of Disease data provides estimates, not indications.

**Response:** As suggested, the term was corrected to “estimate,” in accordance with the nature of Global Burden of Disease data.

L61-63: *“Although underreported, data from the 2019 Global Burden of Disease study estimate that approximately 55.5 million people in the worldwide lived with EDs that year [4].”*

**Methods**

**Comment (Section 2.2, Line 124):** The phrase “from October 10–14, 2024” suggests a fixed time range; please clarify if this is meant to indicate the end date (“until October 14, 2024”).

**Response:** As suggested, Section 2.2 was revised to explicitly clarify the search period.

L122: *“(ProQuest Dissertations and Theses Database and Google Scholar) until October 2024.”*

**Results**

**Comment (Section 3.2):** Present the number of studies rather than percentages.

**Response:** As suggested, absolute numbers of studies were added throughout Sections 3.2 and 3.3. Percentages were retained only when informative and are always accompanied by their respective denominators.

**Comment (Line 222):** There is no clear rationale for aggregating the total number of participants across all studies.  This approach is more appropriate in a meta-analysis, which is not the case here.

**Response:** We thank the reviewer for this important observation. The manuscript was revised to clarify the rationale for presenting the total number of participants. Although this aggregation does not imply quantitative synthesis or meta-analytic inference, it was included to provide a descriptive overview of the populations represented in the included studies, in line with the exploratory and mapping objectives of a scoping review. We explicitly clarified that this aggregation is purely descriptive in nature and does not represent pooled analysis or effect estimation.

**Comment (Line 227):** Please rephrase “another included those with sexual orientation” for clarity. The current phrasing is vague—everyone has a sexual orientation, so please specify the intended meaning.

**Response:** The sentence was revised to clarify that sexual orientation was reported as a sociodemographic characteristic of participants, improving clarity and precision in the description of the sociodemographic variables presented in the study.

L225-226: *“One study included transgender people [46], and another explicitly reported participants’ sexual orientation [105].”*

**Comment (Line 230):** Clarify whether the income figures in brackets refer to monthly income.

**Response:** The text was revised to specify that the income figures refer to monthly income.

L229-230: *“(monthly income between $1000 and $2600 USD) [37,41,45,54].”*

**Comment (Line 231):** Replace “schooling time” with “years of education” or “educational attainment”, as the former suggests daily school hours.

**Response:** The term was replaced with “years of education” to ensure terminological precision.

L230-231: “*whereas three studies reported that the most participants had nine or more years of education”*

**Comment (Line 233):** The sentence “PHC professionals, 233 sixteen revealed that most professionals had medical training” is unclear. Please rephrase for clarity and logical coherence. It also seems obvious that most PHC professionals have medical training—what specific point is being made?

**Response:** The sentence was rewritten for greater clarity and precision, explicitly specifying the professional categories represented.

L231-233: *“Among the 32 studies that included PHC professionals, sixteen predominantly involved physicians, while other health professional categories were underrepresented [58,63,69,72,74–81,83–86].”*

**Comment (Line 243 and similar):** Add the number of studies referenced in brackets to help readers who may not want to count the references manually.

**Response:** The number of studies was added in parentheses throughout the Results section to improve transparency and readability.

**Comment (Line 246):** Provide the full name of each screening tool followed by its acronym in parentheses on first mention.

**Response:** All screening tools are now presented with their full names followed by their respective acronyms at first mention.

**Tables and Figures**

**Comment (Table 2):** Use points rather than commas for decimal formatting.

**Response:** Decimal formatting was standardized using points instead of commas in Table 2.

**Comment (Figure 1):** Consider presenting this as a table instead.

**Response:** As suggested by the reviewer, Figure 1 was converted into a table, ensuring greater clarity, legibility, and alignment with scoping review reporting standards.

**Comment:** The resolution is too low and the text is blurred. Please upload high-quality versions.

**Response:** All figures were replaced with high-resolution versions.

**Comment:** These do not add substantive value to the paper. I recommend removing them and retaining only the tables.

**Response:** The word clouds were removed from the manuscript, and the relevant information was retained in tabular format to improve scientific clarity and interpretability.

**Other important changes made by the authors**

**Title**

In accordance with journal guidelines, the title was revised to sentence case, with capitalization adjusted so that only the first word of the title and subtitle, as well as proper nouns, begin with a capital letter.

Thus, where it previously read: “Validated strategies for screening for eating disorders in primary health care: a scoping review with a focus on adolescents and adults,” it has been revised to read: “Validated strategies for screening for eating disorders in primary health care: *A scoping review with a focus on adolescents and adults*.”

**Affiliations**

According to the journal submission guideline (“Do not include ZIP or Postal Codes, street addresses, or building/office numbers”), the postal code was removed.

L17: *“School of Nutrition, Federal University of Bahia, Salvador, Bahia, Brazil.”*

**Methodology**

The Methods section was updated to reflect the software used in the preparation of analyses and figures following the removal and inclusion of new figures.

The text now reads:

L177–178: *“The analyses and figures were prepared with the support of Microsoft Office (*[*www.office.com*](http://www.office.com/)*), CorelDraw (*[*www.coreldraw.com*](http://www.coreldraw.com/)*), QGIS (*[*https://qgis.org/*](https://qgis.org/)*), and Canva (*[*www.canva.com).”*](http://www.canva.com).xn--ivg/)

This modification does not alter the results or interpretation of the study but ensures greater methodological precision and transparency.

**Correction of section numbering**

During the final revision of the manuscript, we identified inconsistencies in the numbering of sections in the Results. Section 3.3 was incorrectly labeled as 3.1; subsection 3.3.1 as 3.1.1; subsection 3.3.1.1 as 3.1.2; and subsection 3.3.1.2 as 3.1.1. These inconsistencies were corrected to restore proper numbering and logical hierarchy.

**Tables**

The structure of Table 3 (formerly Table 2) was revised for greater clarity. The “criterion validity” column was removed, retaining only the psychometric indices (Se, Sp, PPV, NPV, and AUC). This change improves interpretability without altering the presented data.

**Update of instrument validity description**

The text in lines 279-284 was revised to correctly reflect the type of validity evidence reported. After methodological reassessment, both studies were found to present evidence based on the internal structure of the instrument. One study employed Principal Component Analysis (PCA), demonstrating a strong correlation between the identified components and the total score (r = 0.98, P < 0.001) [54]. The other used Confirmatory Factor Analysis (CFA), supporting the factorial structure of the SCOFF and indicating that the instrument can measure both a single construct and specific dimensions [56].

According to the *Standards for Educational and Psychological Testing*, evidence derived from factor and component analyses corresponds to construct validity based on internal structure, whereas content validity is established through expert judgment regarding item relevance and representativeness. Accordingly, the manuscript and Supplementary Table 1 were revised to properly classify these studies as evidence of construct validity based on internal structure.

Table Suplementar I: “*RQ3: The validity of the SCOFF was evaluated by confirmatory factor analysis”.*

Manucript (lines 292–296): *“Of these, two analyzed the internal structure using Confirmatory Factor Analysis (CFA) and Principal Component Analysis (PCA), whereas eight focused on criterion validity, using the cutoff point = 2. The CFA demonstrated an excellent correlation between the identified factors and the overall score (r = 0.98, P < 0.001) [54]. PCA, on the other hand, concludes that SCOFF can measure both a single construct and specific dimensions [56].”*

**Discussion**

The following sentence was added to the Discussion section for clarity:

L431-433: *“Furthermore, the SCOFF was the only instrument whose internal structure was evaluated through confirmatory factor analysis and principal component analysis [54,56].”*

This addition clarifies that the SCOFF was the only instrument whose internal structure was evaluated using CFA and PCA, providing evidence of construct validity based on internal structure.

**Conclusion**

A new paragraph was added to the Conclusion section to highlight practical contributions and outline recommendations for future research:

L488-499: *“Although this review does not allow for direct clinical recommendations, the findings provide important insights to guide future practice and research. Among these contributions, the potential use of brief, self-administered instruments—such as the SCOFF and the EDE-Q—is noteworthy, particularly in high-demand, resource-limited primary care settings. The adoption of strategies that involve professional training, integration of digital tools, and well-defined referral pathways may enhance the early detection of eating disorders. A research agenda is therefore recommended, focusing on: (i) cross-cultural validation in diverse population groups; (ii) evaluation of the acceptability and applicability of the instruments across different clinical contexts; and (iii) development of training interventions for interdisciplinary teams. Emphasis is placed on the importance of incorporating eating disorder screening as a qualified and ongoing practice in primary care, grounded in validated instruments that are sensitive to local specificities and integrated with mental health care networks.”*

This addition strengthens the practical relevance of the review without altering the original findings or interpretations.

We sincerely thank the reviewer and the editors for their valuable feedback. We believe that these revisions have substantially strengthened the manuscript and hope that it is now suitable for publication.

Sincerely,

**Lisane da Silva Oliveira**
