# Supplementary material for: Validated strategies for screening for eating disorders in primary health care: A scoping review with a focus on adolescents and adults
Source: PLoS One. 2026 Aug 3;21(8):e0347184. doi: 10.1371/journal.pone.0347184 (PMC13432121; doi:10.1371/journal.pone.0347184)
Supplement: Peer review history

# Peer review: Decision Letter 0

**DOI:** 10.1371/journal.pone.0347184.r001

**Authors:** Steve Zimmerman

15 Jan 2026

PONE-D-25-28582

Validated strategies for screening for eating disorders in primary health care: a scoping review with a focus on adolescents and adults

PLOS One

Dear Dr. Oliveira,

Thank you for submitting your manuscript to PLOS ONE. After careful consideration, we feel that it has merit but does not fully meet PLOS ONE’s publication criteria as it currently stands. Therefore, we invite you to submit a revised version of the manuscript that addresses the points raised during the review process.

Please note that we have only been able to secure a single reviewer to assess your manuscript. We are issuing a decision on your manuscript at this point to prevent further delays in the evaluation of your manuscript. Please be aware that the editor who handles your revised manuscript might find it necessary to invite additional reviewers to assess this work once the revised manuscript is submitted.

The reviewer’s comments can be found below. Could you please revise the manuscript to carefully address the concerns raised?

Please submit your revised manuscript by Mar 01 2026 11:59PM. If you will need more time than this to complete your revisions, please reply to this message or contact the journal office at plosone@plos.org. When you’re ready to submit your revision, log on to https://www.editorialmanager.com/pone/ and select the 'Submissions Needing Revision' folder to locate your manuscript file.

Please include the following items when submitting your revised manuscript:

- A letter that responds to each point raised by the academic editor and reviewer(s). You should upload this letter as a separate file labeled 'Response to Reviewers'.
- A marked-up copy of your manuscript that highlights changes made to the original version. You should upload this as a separate file labeled 'Revised Manuscript with Track Changes'.
- An unmarked version of your revised paper without tracked changes. You should upload this as a separate file labeled 'Manuscript'.

-->If you would like to make changes to your financial disclosure, please include your updated statement in your cover letter. Guidelines for resubmitting your figure files are available below the reviewer comments at the end of this letter.

If applicable, we recommend that you deposit your laboratory protocols in protocols.io to enhance the reproducibility of your results. Protocols.io assigns your protocol its own identifier (DOI) so that it can be cited independently in the future. For instructions see: https://journals.plos.org/plosone/s/submission-guidelines#loc-laboratory-protocols. Additionally, PLOS ONE offers an option for publishing peer-reviewed Lab Protocol articles, which describe protocols hosted on protocols.io. Read more information on sharing protocols at https://plos.org/protocols?utm_medium=editorial-email&utm_source=authorletters&utm_campaign=protocols.

We look forward to receiving your revised manuscript.

Kind regards,

Steve Zimmerman, PhD

Senior Editor, PLOS One

**Journal Requirements:**

When submitting your revision, we need you to address these additional requirements.

1. Please ensure that your manuscript meets PLOS ONE's style requirements, including those for file naming. The PLOS ONE style templates can be found at

https://journals.plos.org/plosone/s/file?id=wjVg/PLOSOne_formatting_sample_main_body.pdf and

https://journals.plos.org/plosone/s/file?id=ba62/PLOSOne_formatting_sample_title_authors_affiliations.pdf

2. Thank you for stating the following financial disclosure:

“This work was supported by the National Council for Scientific and Technological Development (CNPq) and the Department of Science and Technology of the Secretariat of Science, Technology, Innovation and Strategic Inputs in Health of the Ministry of Health (Decit/SCTIE/MS). Process number: 408315/2022–9.”

Please state what role the funders took in the study.  If the funders had no role, please state: "The funders had no role in study design, data collection and analysis, decision to publish, or preparation of the manuscript."

If this statement is not correct you must amend it as needed.

Please include this amended Role of Funder statement in your cover letter; we will change the online submission form on your behalf.

3. Thank you for stating the following in the Acknowledgments Section of your manuscript:

“The authors acknowledge the financial support provided by the Coordination for the Improvement of Higher Education Personnel (CAPES), Brazil, Code 001.”

We note that you have provided funding information that is not currently declared in your Funding Statement. However, funding information should not appear in the Acknowledgments section or other areas of your manuscript. We will only publish funding information present in the Funding Statement section of the online submission form.

Please remove any funding-related text from the manuscript and let us know how you would like to update your Funding Statement. Currently, your Funding Statement reads as follows:

“This work was supported by the National Council for Scientific and Technological Development (CNPq) and the Department of Science and Technology of the Secretariat of Science, Technology, Innovation and Strategic Inputs in Health of the Ministry of Health (Decit/SCTIE/MS). Process number: 408315/2022–9.”

Please include your amended statements within your cover letter; we will change the online submission form on your behalf.

4. We note that Figure 3 in your submission contain [map/satellite] images which may be copyrighted. All PLOS content is published under the Creative Commons Attribution License (CC BY 4.0), which means that the manuscript, images, and Supporting Information files will be freely available online, and any third party is permitted to access, download, copy, distribute, and use these materials in any way, even commercially, with proper attribution. For these reasons, we cannot publish previously copyrighted maps or satellite images created using proprietary data, such as Google software (Google Maps, Street View, and Earth). For more information, see our copyright guidelines: http://journals.plos.org/plosone/s/licenses-and-copyright.

We require you to either (1) present written permission from the copyright holder to publish these figures specifically under the CC BY 4.0 license, or (2) remove the figures from your submission:

1. You may seek permission from the original copyright holder of Figure 3 to publish the content specifically under the CC BY 4.0 license.

We recommend that you contact the original copyright holder with the Content Permission Form (http://journals.plos.org/plosone/s/file?id=7c09/content-permission-form.pdf) and the following text:

“I request permission for the open-access journal PLOS ONE to publish XXX under the Creative Commons Attribution License (CCAL) CC BY 4.0 (http://creativecommons.org/licenses/by/4.0/). Please be aware that this license allows unrestricted use and distribution, even commercially, by third parties. Please reply and provide explicit written permission to publish XXX under a CC BY license and complete the attached form.”

Please upload the completed Content Permission Form or other proof of granted permissions as an "Other" file with your submission.

In the figure caption of the copyrighted figure, please include the following text: “Reprinted from [ref] under a CC BY license, with permission from [name of publisher], original copyright [original copyright year].”

2. If you are unable to obtain permission from the original copyright holder to publish these figures under the CC BY 4.0 license or if the copyright holder’s requirements are incompatible with the CC BY 4.0 license, please either i) remove the figure or ii) supply a replacement figure that complies with the CC BY 4.0 license. Please check copyright information on all replacement figures and update the figure caption with source information. If applicable, please specify in the figure caption text when a figure is similar but not identical to the original image and is therefore for illustrative purposes only.

The following resources for replacing copyrighted map figures may be helpful:

USGS National Map Viewer (public domain): http://viewer.nationalmap.gov/viewer/

The Gateway to Astronaut Photography of Earth (public domain): http://eol.jsc.nasa.gov/sseop/clickmap/

Maps at the CIA (public domain): https://www.cia.gov/library/publications/the-world-factbook/index.html and https://www.cia.gov/library/publications/cia-maps-publications/index.html

NASA Earth Observatory (public domain): http://earthobservatory.nasa.gov/

Landsat: http://landsat.visibleearth.nasa.gov/

USGS EROS (Earth Resources Observatory and Science (EROS) Center) (public domain): http://eros.usgs.gov/#

Natural Earth (public domain): http://www.naturalearthdata.com/

5. Please upload a new copy of Figures 1, 2, 3, 5, and 6 as the detail is not clear. Please follow the link for more information:  https://journals.plos.org/plosone/s/figures

6. If the reviewer comments include a recommendation to cite specific previously published works, please review and evaluate these publications to determine whether they are relevant and should be cited. There is no requirement to cite these works unless the editor has indicated otherwise.

7. Please review your reference list to ensure that it is complete and correct. If you have cited papers that have been retracted, please include the rationale for doing so in the manuscript text, or remove these references and replace them with relevant current references. Any changes to the reference list should be mentioned in the rebuttal letter that accompanies your revised manuscript. If you need to cite a retracted article, indicate the article’s retracted status in the References list and also include a citation and full reference for the retraction notice.

[Note: HTML markup is below. Please do not edit.]

Reviewers' comments:

Reviewer's Responses to Questions

**Comments to the Author**

1. Is the manuscript technically sound, and do the data support the conclusions?

The manuscript must describe a technically sound piece of scientific research with data that supports the conclusions. Experiments must have been conducted rigorously, with appropriate controls, replication, and sample sizes. The conclusions must be drawn appropriately based on the data presented.

Reviewer #1: Yes

**********

2. Has the statistical analysis been performed appropriately and rigorously?

Reviewer #1: N/A

**********

3. Have the authors made all data underlying the findings in their manuscript fully available?

The PLOS Data policy requires authors to make all data underlying the findings described in their manuscript fully available without restriction, with rare exception (please refer to the Data Availability Statement in the manuscript PDF file). The data should be provided as part of the manuscript or its supporting information, or deposited to a public repository. For example, in addition to summary statistics, the data points behind means, medians and variance measures should be available. If there are restrictions on publicly sharing data—e.g. participant privacy or use of data from a third party—those must be specified.

Reviewer #1: Yes

**********

4. Is the manuscript presented in an intelligible fashion and written in standard English?

PLOS ONE does not copyedit accepted manuscripts, so the language in submitted articles must be clear, correct, and unambiguous. Any typographical or grammatical errors should be corrected at revision, so please note any specific errors here.

Reviewer #1: Yes

**********

5. Review Comments to the Author

Please use the space provided to explain your answers to the questions above. You may also include additional comments for the author, including concerns about dual publication, research ethics, or publication ethics. (Please upload your review as an attachment if it exceeds 20,000 characters)

Reviewer #1: I thank the Editor for the opportunity to review the paper titled « Validated strategies for screening for eating disorders in primary health care: a scoping review with a focus on adolescents and adults » by da Silva Oliveira.

The paper is well-written. I have only minor revisions mainly for clarification.

Abstract

Replace “investigate” with a more precise verb such as “provide an overview” or “map”.

Add the OSF registration number in the abstract.

Cite the six databases used.

Expand on the identified gaps to offer more insight.

Ensure consistency between the abstract submitted to the system and the one included in the main manuscript.

Introduction

Line 63: Replace “indicate” with “estimate”. The Global Burden of Disease data provides estimates, not indications.

Method

Section 2.2, Line 124: The phrase “from October 10–14, 2024” suggests a fixed time range; please clarify if this is meant to indicate the end date (“until October 14, 2024”).

Results

Section 3.2: Present the number of studies rather than percentages. The total number of included studies is not always given, which makes interpreting percentages (e.g., 50%, 30.4%) difficult.

Line 222: There is no clear rationale for aggregating the total number of participants across all studies. This approach is more appropriate in a meta-analysis, which is not the case here.

Line 227: Please rephrase “another included those with sexual orientation” for clarity. The current phrasing is vague—everyone has a sexual orientation, so please specify the intended meaning.

Line 230: Clarify whether the income figures in brackets refer to monthly income.

Line 231: Replace “schooling time” with “years of education” or “educational attainment”, as the former suggests daily school hours.

Line 233: The sentence “PHC professionals, 233 sixteen revealed that most professionals had medical training” is unclear. Please rephrase for clarity and logical coherence. It also seems obvious that most PHC professionals have medical training—what specific point is being made?

Line 243 (and similar sections): Add the number of studies referenced in brackets to help readers who may not want to count the references manually.

Line 246: Provide the full name of each screening tool followed by its acronym in parentheses on first mention.

Table 2: Use points rather than commas for decimal formatting (e.g., 0.7 instead of 0,7).

Figure 1: Consider presenting this as a table instead.

All figures: The resolution is too low and the text is blurred. Please upload high-quality versions.

Word clouds: These do not add substantive value to the paper. I recommend removing them and retaining only the tables.

**********

6. PLOS authors have the option to publish the peer review history of their article (what does this mean?). If published, this will include your full peer review and any attached files.

If you choose “no”, your identity will remain anonymous but your review may still be made public.

**Do you want your identity to be public for this peer review?**  For information about this choice, including consent withdrawal, please see our Privacy Policy.

Reviewer #1: **Yes:** Sophie Pilleron

**********

[NOTE: If reviewer comments were submitted as an attachment file, they will be attached to this email and accessible via the submission site. Please log into your account, locate the manuscript record, and check for the action link "View Attachments". If this link does not appear, there are no attachment files.]

To ensure your figures meet our technical requirements, please review our figure guidelines: https://journals.plos.org/plosone/s/figures

You may also use PLOS’s free figure tool, NAAS, to help you prepare publication quality figures: https://journals.plos.org/plosone/s/figures#loc-tools-for-figure-preparation.

NAAS will assess whether your figures meet our technical requirements by comparing each figure against our figure specifications.

# Author comment: Author response to Decision Letter 1

**DOI:** 10.1371/journal.pone.0347184.r002

1 Mar 2026

Response to Reviewers – Manuscript PONE-D-25-28582

Dear Editors and Reviewer,

We would like to thank you for the careful evaluation of our manuscript entitled “Validated strategies for screening for eating disorders in primary health care: a scoping review with a focus on adolescents and adults” (Manuscript ID: PONE-D-25-28582). We appreciate the constructive and detailed comments, which substantially contributed to improving the clarity, transparency, and overall quality of the manuscript.

We carefully addressed all comments raised by the reviewer and fulfilled the journal requirements. All changes are highlighted in the revised manuscript using the track changes feature. Below, we present a detailed point-by-point response.

Responses to Journal Requirements

Requirement 1 – PLOS ONE style and formatting

The manuscript was thoroughly reviewed to comply with the PLOS ONE formatting and style guidelines.

Requirement 2 – Role of the funders

We confirm that the funders had no role in study design, data collection and analysis, decision to publish, or preparation of the manuscript. The following statement was included in the cover letter, as requested:

“The funders had no role in study design, data collection and analysis, decision to publish, or preparation of the manuscript.”

Requirement 3 – Funding information in the Acknowledgments

All funding-related information was removed from the Acknowledgments section. The Funding Statement was maintained exclusively in the appropriate section of the online submission system, as requested.

L624-626: “The authors acknowledge the support provided by the Coordination for the Improvement of Higher Education Personnel (CAPES), Brazil, Code 001. We would also like to thank librarian Gesner Francisco Xavier for his assistance in the development of the preliminary search strategy.”

Requirement 4 – Copyright issues related to Figure 3

Figure 3 was removed and replaced with a new figure developed by the authors using public domain data, fully compatible with the CC BY 4.0 license. The figure caption was updated to comply with PLOS ONE attribution and licensing requirements (CC BY 4.0).

Requirement 5 – Figure quality

High-resolution versions of both figures (Fig 1 and Fig 2) were submitted in accordance with the PLOS ONE figure preparation guidelines. In response to the reviewer’s comments, the former Figure 1 was removed and its content was incorporated into a table. The former Figure 2 was resubmitted and renumbered as Figure 1. The former Figure 3 was replaced and renumbered as Figure 2. Figure 4 was retained and renumbered as Figure 3. Former Figures 5 and 6 were removed from the manuscript.

Requirement 6 – Evaluation of citation recommendations

The reviewer comments did not include explicit recommendations to cite specific previously published works. Nevertheless, the authors critically reviewed the relevant literature to ensure that the cited studies remain current, relevant, and aligned with the objectives of the review.

Requirement 7 – Review of the reference list

The reference list was carefully reviewed to ensure its completeness, accuracy, and currency. No retracted articles were identified among the cited references. The references remain consistent with the scope, objectives, and methodological rigor of the study.

Responses to Reviewer #1

We thank the reviewer for the positive evaluation of the manuscript and for the valuable suggestions. All comments were carefully analyzed and addressed as follows:

Abstract

Comment 1: Replace “investigate” with a more precise verb such as “provide an overview” or “map”.

Response: As suggested, the verb “investigate” was replaced with “map” to better reflect the objectives of a scoping review.

L33–35: “This scoping review aimed to map the available evidence on validated strategies for screening for eating disorders in adolescents and adults in primary care.”

Additionally, we clarified the aim of the review in the Introduction:

L93-94: “The aim of this review is to map the extent of the available evidence on validated strategies for ED screening in adolescents and adults assisted by PHC.”

Comment 2: Add the OSF registration number in the abstract.

Response: As requested, the OSF registration number was included in the abstract.

L35-37: “The study followed the Preferred Reporting Items for Systematic reviews and Meta-Analyses extension for Scoping Reviews, with a protocol registered in the Open Science Framework (doi: 10.17605/OSF.IO/498FU).”

Comment 3: Cite the six databases used.

Response: As suggested, the six databases were specified in the abstract:

L37-39: “Searches were conducted in the databases MEDLINE, Embase, Latin American and Caribbean Health Sciences Literature, Web of Science, PsycINFO, and Cumulative Index to Nursing and Allied Health Literature.”

Comment 4: Expand on the identified gaps to offer more insight.

Response: The abstract was revised as suggested to provide greater clarity and a more detailed description of the main evidence gaps identified. To comply with the 300-word limit, conciseness and rephrasing adjustments were made without loss of relevant content.

L50-55: “Despite the potential of several instruments, robust evidence on their validity, reliability, and applicability remains limited, particularly in low- and middle-income settings and among underrepresented and adolescent populations. Structural and training barriers hinder the effective implementation of screening, highlighting the need for articulated actions between professionals, services, and public policies.”

Comment 5: Ensure consistency between the abstract submitted to the system and the one included in the main manuscript.

Response: Full consistency between the abstract submitted to the system and the abstract included in the main manuscript was ensured.

Introduction

Comment (Line 63): Replace “indicate” with “estimate”. The Global Burden of Disease data provides estimates, not indications.

Response: As suggested, the term was corrected to “estimate,” in accordance with the nature of Global Burden of Disease data.

L61-63: “Although underreported, data from the 2019 Global Burden of Disease study estimate that approximately 55.5 million people in the worldwide lived with EDs that year [4].”

Methods

Comment (Section 2.2, Line 124): The phrase “from October 10–14, 2024” suggests a fixed time range; please clarify if this is meant to indicate the end date (“until October 14, 2024”).

Response: As suggested, Section 2.2 was revised to explicitly clarify the search period.

L122: “(ProQuest Dissertations and Theses Database and Google Scholar) until October 2024.”

Results

Comment (Section 3.2): Present the number of studies rather than percentages.

Response: As suggested, absolute numbers of studies were added throughout Sections 3.2 and 3.3. Percentages were retained only when informative and are always accompanied by their respective denominators.

Comment (Line 222): There is no clear rationale for aggregating the total number of participants across all studies. This approach is more appropriate in a meta-analysis, which is not the case here.

Response: We thank the reviewer for this important observation. The manuscript was revised to clarify the rationale for presenting the total number of participants. Although this aggregation does not imply quantitative synthesis or meta-analytic inference, it was included to provide a descriptive overview of the populations represented in the included studies, in line with the exploratory and mapping objectives of a scoping review. We explicitly clarified that this aggregation is purely descriptive in nature and does not represent pooled analysis or effect estimation.

Comment (Line 227): Please rephrase “another included those with sexual orientation” for clarity. The current phrasing is vague—everyone has a sexual orientation, so please specify the intended meaning.

Response: The sentence was revised to clarify that sexual orientation was reported as a sociodemographic characteristic of participants, improving clarity and precision in the description of the sociodemographic variables presented in the study.

L225-226: “One study included transgender people [46], and another explicitly reported participants’ sexual orientation [105].”

Comment (Line 230): Clarify whether the income figures in brackets refer to monthly income.

Response: The text was revised to specify that the income figures refer to monthly income.

L229-230: “(monthly income between $1000 and $2600 USD) [37,41,45,54].”

Comment (Line 231): Replace “schooling time” with “years of education” or “educational attainment”, as the former suggests daily school hours.

Response: The term was replaced with “years of education” to ensure terminological precision.

L230-231: “whereas three studies reported that the most participants had nine or more years of education”

Comment (Line 233): The sentence “PHC professionals, 233 sixteen revealed that most professionals had medical training” is unclear. Please rephrase for clarity and logical coherence. It also seems obvious that most PHC professionals have medical training—what specific point is being made?

Response: The sentence was rewritten for greater clarity and precision, explicitly specifying the professional categories represented.

L231-233: “Among the 32 studies that included PHC professionals, sixteen predominantly involved physicians, while other health professional categories were underrepresented [58,63,69,72,74–81,83–86].”

Comment (Line 243 and similar): Add the number of studies referenced in brackets to help readers who may not want to count the references manually.

Response: The number of studies was added in parentheses throughout the Results section to improve transparency and readability.

Comment (Line 246): Provide the full name of each screening tool followed by its acronym in parentheses on first mention.

Response: All screening tools are now presented with their full names followed by their respective acronyms at first mention.

Tables and Figures

Comment (Table 2): Use points rather than commas for decimal formatting.

Response: Decimal formatting was standardized using points instead of commas in Table 2.

Comment (Figure 1): Consider presenting this as a table instead.

Response: As suggested by the reviewer, Figure 1 was converted into a table, ensuring greater clarity, legibility, and alignment with scoping review reporting standards.

Comment: The resolution is too low and the text is blurred. Please upload high-quality versions.

Response: All figures were replaced with high-resolution versions.

Comment: These do not add substantive value to the paper. I recommend removing them and retaining only the tables.

Response: The word clouds were removed from the manuscript, and the relevant information was retained in tabular format to improve scientific clarity and interpretability.

Other important changes made by the authors

Title

In accordance with journal guidelines, the title was revised to sentence case, with capitalization adjusted so that only the first word of the title and subtitle, as well as proper nouns, begin with a capital letter.

Thus, where it previously read: “Validated strategies for screening for eating disorders in primary health care: a scoping review with a focus on adolescents and adults,” it has been revised to read: “Validated strategies for screening for eating disorders in primary health care: A scoping review with a focus on adolescents and adults.”

Affiliations

According to the journal submission guideline (“Do not include ZIP or Postal Codes, street addresses, or building/office numbers”), the postal code was removed.

L17: “School of Nutrition, Federal University of Bahia, Salvador, Bahia, Brazil.”

Methodology

The Methods section was updated to reflect the software used in the preparation of analyses and figures following the removal and inclusion of new figures.

The text now reads:

L177–178: “The analyses and figures were prepared with the support of Microsoft Office (www.office.com), CorelDraw (www.coreldraw.com), QGIS (https://qgis.org/), and Canva (www.canva.com).”

This modification does not alter the results or interpretation of the study but ensures greater methodological precision and transparency.

Correction of section numbering

During the final revision of the manuscript, we identified inconsistencies in the numbering of sections in the Results. Section 3.3 was incorrectly labeled as 3.1; subsection 3.3.1 as 3.1.1; subsection 3.3.1.1 as 3.1.2; and subsection 3.3.1.2 as 3.1.1. These inconsistencies were corrected to restore proper numbering and logical hierarchy.

Tables

The structure of Table 3 (formerly Table 2) was revised for greater clarity. The “criterion validity” column was removed, retaining only the psychometric indices (Se, Sp, PPV, NPV, and AUC). This change improves interpretability without altering the presented data.

Update of instrument validity description

The text in lines 279-284 was revised to correctly reflect the type of validity evidence reported. After methodological reassessment, both studies were found to present evidence based on the internal structure of the instrument. One study employed Principal Component Analysis (PCA), demonstrating a strong correlation between the identified components and the total score (r = 0.98, P < 0.001) [54]. The other used Confirmatory Factor Analysis (CFA), supporting the factorial structure of the SCOFF and indicating that the instrument can measure both a single construct and specific dimensions [56].

According to the Standards for Educational and Psychological Testing, evidence derived from factor and component analyses corresponds to construct validity based on internal structure, whereas content validity is established through expert judgment regarding item relevance and representativeness. Accordingly, the manuscript and Supplementary Table 1 were revised to properly classify these studies as evidence of construct validity based on internal structure.

Table Suplementar I: “RQ3: The validity of the SCOFF was evaluated by confirmatory factor analysis”.

Manucript (lines 292–296): “Of these, two analyzed the internal structure using Confirmatory Factor Analysis (CFA) and Principal Component Analysis (PCA), whereas eight focused on criterion validity, using the cutoff point = 2. The CFA demonstrated an excellent correlation between the identified factors and the overall score (r = 0.98, P < 0.001) [54]. PCA, on the other hand, concludes that SCOFF can measure both a single construct and specific dimensions [56].”

Discussion

The following sentence was added to the Discussion section for clarity:

L431-433: “Furthermore, the SCOFF was the only instrument whose internal structure was evaluated through confirmatory factor analysis and principal component analysis [54,56].”

This addition clarifies that the SCOFF was the only instrument whose internal structure was evaluated using CFA and PCA, providing evidence of construct validity based on internal structure.

Conclusion

A new paragraph was added to the Conclusion section to highlight practical contributions and outline recommendations for future research:

L488-499: “Although this review does not allow for direct clinical recommendations, the findings provide important insights to guide future practice and research. Among these contributions, the potential use of brief, self-administered instruments—such as the SCOFF and the EDE-Q—is noteworthy, particularly in high-demand, resource-limited primary care settings. The adoption of strategies that involve professional training, integration of digital tools, and well-defined referral pathways may enhance the early detection of eating disorders. A research agenda is therefore recommended, focusing on: (i) cross-cultural validation in diverse population groups; (ii) evaluation of the acceptability and applicability of the instruments across different clinical contexts; and (iii) development of training interventions for interdisciplinary teams. Emphasis is placed on the importance of incorporating eating disorder screening as a qualified and ongoing practice in primary care, grounded in val

Attachment Submitted filename: Response to Reviwes.docx

# Peer review: Decision Letter 1

**DOI:** 10.1371/journal.pone.0347184.r003

**Authors:** Ilse Bloom

20 Apr 2026

<div>

PONE-D-25-28582R1

Validated strategies for screening for eating disorders in primary health care: A scoping review with a focus on adolescents and adults

PLOS One

Dear Dr. Oliveira,

Thank you for submitting your manuscript to PLOS ONE. After careful consideration, we feel that it has merit but does not fully meet PLOS ONE’s publication criteria as it currently stands. Therefore, we invite you to submit a revised version of the manuscript that addresses the points raised during the review process.

The manuscript has been further evaluated by two reviewers, and their comments are available below.

Could you please carefully revise the manuscript to address all comments raised?

Please submit your revised manuscript by Jun 04 2026 11:59PM. If you will need more time than this to complete your revisions, please reply to this message or contact the journal office at plosone@plos.org. When you're ready to submit your revision, log on to https://www.editorialmanager.com/pone/ and select the 'Submissions Needing Revision' folder to locate your manuscript file.

Please include the following items when submitting your revised manuscript:

- A letter that responds to each point raised by the academic editor and reviewer(s). You should upload this letter as a separate file labeled 'Response to Reviewers'.
- A marked-up copy of your manuscript that highlights changes made to the original version. You should upload this as a separate file labeled 'Revised Manuscript with Track Changes'.
- An unmarked version of your revised paper without tracked changes. You should upload this as a separate file labeled 'Manuscript'.

If you would like to make changes to your financial disclosure, please include your updated statement in your cover letter. Guidelines for resubmitting your figure files are available below the reviewer comments at the end of this letter.

If applicable, we recommend that you deposit your laboratory protocols in protocols.io to enhance the reproducibility of your results. Protocols.io assigns your protocol its own identifier (DOI) so that it can be cited independently in the future. For instructions see: https://journals.plos.org/plosone/s/submission-guidelines#loc-laboratory-protocols. Additionally, PLOS ONE offers an option for publishing peer-reviewed Lab Protocol articles, which describe protocols hosted on protocols.io. Read more information on sharing protocols at https://plos.org/protocols?utm_medium=editorial-email&utm_source=authorletters&utm_campaign=protocols.

As the corresponding author, your ORCID iD is verified in the submission system and will appear in the published article. PLOS supports the use of ORCID, and we encourage all coauthors to register for an ORCID iD and use it as well. Please encourage your coauthors to verify their ORCID iD within the submission system before final acceptance, as unverified ORCID iDs will not appear in the published article. *Only*  the individual author can complete the verification step; PLOS staff *cannot*  verify ORCID iDs on behalf of authors.

We look forward to receiving your revised manuscript.

Kind regards,

Ilse Bloom

Staff Editor

PLOS One

**Journal Requirements:**

1. If the reviewer comments include a recommendation to cite specific previously published works, please review and evaluate these publications to determine whether they are relevant and should be cited. There is no requirement to cite these works unless the editor has indicated otherwise.

2. Please review your reference list to ensure that it is complete and correct. If you have cited papers that have been retracted, please include the rationale for doing so in the manuscript text, or remove these references and replace them with relevant current references. Any changes to the reference list should be mentioned in the rebuttal letter that accompanies your revised manuscript. If you need to cite a retracted article, indicate the article’s retracted status in the References list and also include a citation and full reference for the retraction notice.

[Note: HTML markup is below. Please do not edit.]

Reviewers' comments:

Reviewer's Responses to Questions

**Comments to the Author**

1. If the authors have adequately addressed your comments raised in a previous round of review and you feel that this manuscript is now acceptable for publication, you may indicate that here to bypass the “Comments to the Author” section, enter your conflict of interest statement in the “Confidential to Editor” section, and submit your "Accept" recommendation.

Reviewer #1: All comments have been addressed

Reviewer #2: (No Response)

**********

2. Is the manuscript technically sound, and do the data support the conclusions?

The manuscript must describe a technically sound piece of scientific research with data that supports the conclusions. Experiments must have been conducted rigorously, with appropriate controls, replication, and sample sizes. The conclusions must be drawn appropriately based on the data presented.

Reviewer #1: Yes

Reviewer #2: Yes

**********

3. Has the statistical analysis been performed appropriately and rigorously?

Reviewer #1: N/A

Reviewer #2: N/A

**********

4. Have the authors made all data underlying the findings in their manuscript fully available?

The PLOS Data policy requires authors to make all data underlying the findings described in their manuscript fully available without restriction, with rare exception (please refer to the Data Availability Statement in the manuscript PDF file). The data should be provided as part of the manuscript or its supporting information, or deposited to a public repository. For example, in addition to summary statistics, the data points behind means, medians and variance measures should be available. If there are restrictions on publicly sharing data—e.g. participant privacy or use of data from a third party—those must be specified.

Reviewer #1: Yes

Reviewer #2: Yes

**********

5. Is the manuscript presented in an intelligible fashion and written in standard English?

PLOS ONE does not copyedit accepted manuscripts, so the language in submitted articles must be clear, correct, and unambiguous. Any typographical or grammatical errors should be corrected at revision, so please note any specific errors here.

Reviewer #1: Yes

Reviewer #2: Yes

**********

6. Review Comments to the Author

Please use the space provided to explain your answers to the questions above. You may also include additional comments for the author, including concerns about dual publication, research ethics, or publication ethics. (Please upload your review as an attachment if it exceeds 20,000 characters)

Reviewer #1: I thank the authors for taking into account my previous comments. I have only minor comments. Congratulations.

Line 154: The text currently refers to “a fifth reviewer.” Should this instead be “a third reviewer”? Please clarify the intended number of reviewers.

Use of Covidence: There is no need to write out the full name of Covidence. The short form “Covidence” is sufficient throughout.

Percentages and Study Counts: For clarity, please present percentages and the corresponding number of studies within the same parentheses (e.g., “25% (n = X)”).

Line 250: Please expand the acronym VA‑BES at first use for clarity.

Table 3: The value PPV = 5 for the SCOFF questionnaire appears very low. Please verify this value and correct it if necessary.

Line 300 and similar headings: Please adjust the formatting of this heading (and other similar headings) to clearly distinguish it from regular body text.

Table 4: The table formatting needs revision—several horizontal lines appear to be missing. Please ensure consistent and complete table borders.

Line 348: Please move reference [73] so that it appears at the end of the sentence.

Line 353: Please replace “and one was scoping” with “one was a scoping review.”

Reviewer #2: To the authors,

This review is sure to be helpful to ED researchers, medical educators, and practicing health professionals. I was not a first-round reviewer but appreciate your edits in response to Reviewer 1. My recommendation is for a minor revision, primarily focused on consistency and clarity in how descriptions of the studies are framed throughout the paper, as it is difficult to track within the scope of how many total studies were included, once in the thick of the paper.

Specific examples of where clarity can be improved:

“Among the 32 studies that included PHC professionals, sixteen predominantly involved physicians, while other health professional categories were underrepresented. For more details on demographic and socioeconomic characteristics, see Table S1. Forty-five studies were conducted exclusively in PHC, nine studies in environments generalizable to PHC, such as university-affiliated services and hospital-affiliated services, and two combined both contexts. Of these, 31 studies were conducted in urban and metropolitan areas.” - Needs better contextualization within the total number of studies included, rather than the number of studies that included professionals within the total number of studies.

General language example: Of the X total of studies that met inclusion criteria, Y mentioned ____. Of these, ___ percent or ___ number had these traits.

Table 4 - Table 4 - Main barriers and facilitators to screening reported by professionals – Can enhance clarity by nesting frequency within total number of relevant articles.

Line 228: “Four studies revealed that the majority of users were married and had low to intermediate family income (monthly income between $1000 and $2600 USD) whereas three studies reported that the most participants had nine or more years of education”

Minor edits:

Line 98 “What are the characteristics of the validated strategies for at screening and their application...” - The language “for at” is unclear and potentially a typo.

Make sure all “eating disorders” are abbreviated to ED (e.g., line 393)

Line 327 - “Female professionals showed a greater tendency to adopt the screening questionnaire...” Can add existing citations that this is consistent with literature showing that males are less likely to screen.

Conceptual Explanations:

While I can infer as a reviewer, please provide a rationale for excluding ARFID.

Other Comments:

Lines 401 to 408 can use another review and tighter editing.

Line 401: “Even so, these tools have limitations in terms of diagnostic accuracy, being subject to false positives and, therefore, generating unnecessary referrals” - This feels a bit narrow and not robustly supported. I recommend rephrasing to “being subject to both false positives and missed capture” if keeping.

“In addition, screening can influence the trajectory of care, increasing the frequency of consultations and referrals without robust evidence that this practice effectively improves clinical outcome” - This feels dangerous to include and not robustly supported. Literature points to the importance of early intervention within first three years of illness onset as instrumental to positive clinical outcomes.

The above two statements and the section in general needs to be conceptually merged with this stronger point “Additionally, another critical point concerns the cultural biases present in screening instruments, which tend to privilege cultural stereotypes of gender, race, and social class, for example, the profile of white women from the middle to upper classes, as opposed to the neglect of populations with greater vulnerability, such as those in situations of food insecurity [106].”

**********

7. PLOS authors have the option to publish the peer review history of their article (what does this mean?). If published, this will include your full peer review and any attached files.

If you choose “no”, your identity will remain anonymous but your review may still be made public.

**Do you want your identity to be public for this peer review?**  For information about this choice, including consent withdrawal, please see our Privacy Policy.

Reviewer #1: **Yes:** Sophie PILLERON

Reviewer #2: **Yes:** Scout Silverstein

**********

[NOTE: If reviewer comments were submitted as an attachment file, they will be attached to this email and accessible via the submission site. Please log into your account, locate the manuscript record, and check for the action link "View Attachments". If this link does not appear, there are no attachment files.]

To ensure your figures meet our technical requirements, please review our figure guidelines: https://journals.plos.org/plosone/s/figures

You may also use PLOS’s free figure tool, NAAS, to help you prepare publication quality figures: https://journals.plos.org/plosone/s/figures#loc-tools-for-figure-preparation.

NAAS will assess whether your figures meet our technical requirements by comparing each figure against our figure specifications.

Attachment Submitted filename: Peer Review PLoS One.pdf

# Author comment: Author response to Decision Letter 2

**DOI:** 10.1371/journal.pone.0347184.r004

1 Jun 2026

Response to Reviewers – Manuscript PONE-D-25-28582

Dear Editors and Reviewer,

We would like to thank you for the careful evaluation of our manuscript entitled “Validated strategies for screening for eating disorders in primary health care: a scoping review with a focus on adolescents and adults” (Manuscript ID: PONE-D-25-28582). We appreciate the constructive and detailed comments, which substantially contributed to improving the clarity, transparency, and overall quality of the manuscript.

We carefully addressed all comments raised by the reviewer and fulfilled the journal requirements. All changes are highlighted in the revised manuscript using the track changes feature. Below, we present a detailed point-by-point response.

Responses to Reviewer #1

We thank the reviewer for the positive evaluation of the manuscript and for the valuable suggestions. All comments were carefully analyzed and addressed as follows:

Comment 1: Line 154: The text currently refers to “a fifth reviewer.” Should this instead be “a third reviewer”? Please clarify the intended number of reviewers.

Response: As suggested, the term “a fifth reviewer” was corrected to “a third reviewer” to accurately reflect the review process.

L153-154: Any disagreement between each pair was resolved through discussion or, if necessary, with the mediation of a third reviewer.

Comment 2: Use of Covidence: There is no need to write out the full name of Covidence. The short form “Covidence” is sufficient throughout.

Response: As suggested, the manuscript was revised and the platform is now referred to only as “Covidence” throughout the text.

Comment 3: Percentages and Study Counts: For clarity, please present percentages and the corresponding number of studies within the same parentheses (e.g., “25% (n = X)”).

Response: As suggested, the presentation of percentages and study counts was revised throughout the manuscript to improve clarity and readability while avoiding excessive use of parentheses.

Comment 4: Line 250: Please expand the acronym VA‑BES at first use for clarity.

Response: As requested, the acronym VA-BES was expanded at first mention in the manuscript.

L267-247: The number of items in the instruments ranged from one item in the Veterans Affairs Binge Eating Screening (VA-BES) (...)

Comment 5: Table 3: The value PPV = 5 for the SCOFF questionnaire appears very low. Please verify this value and correct it if necessary.

Response: Thank you for identifying this issue. The value was reviewed and corrected in Table 3.

Commet 6: Line 300 and similar headings: Please adjust the formatting of this heading (and other similar headings) to clearly distinguish it from regular body text.

Response: As suggested, the formatting of this heading and similar headings throughout the manuscript was revised to improve distinction from the main body text.

L300: “3.3.1.3 Feasibility of screening (RQ4)”

Comment 7: Table 4: The table formatting needs revision—several horizontal lines appear to be missing. Please ensure consistent and complete table borders.

Response: As requested, Table 4 formatting was revised and the horizontal borders were standardized to ensure consistency throughout the table. Additionally, the table was reorganized to present barriers and facilitators according to the prevalence of studies reporting them, improving clarity and data presentation.

Comment 8: Line 348: Please move reference [73] so that it appears at the end of the sentence.

Response: As suggested, reference [73] was moved to the end of the sentence.

L346-347: (...) and the existence of a multidisciplinary team for treatment, which were considered potent facilitators in one study (Table 4 and Table S1) [73].

Comment 9: Line 353: Please replace “and one was scoping” with “one was a scoping review.”

Response: As suggested, the sentence was revised for grammatical accuracy and clarity.

L352: (...) one was a scoping review.

Responses to Reviewer #2

We thank the reviewer for the positive evaluation of the manuscript and for the valuable suggestions. All comments were carefully analyzed and addressed as follows:

Reviewer #2:

We thank the reviewer for the careful reading of the manuscript and for the constructive suggestions. We appreciate the recognition of the relevance of this review to ED researchers, medical educators, and health professionals. All comments were carefully considered, and the manuscript was revised accordingly.

Comment 1: This review is sure to be helpful to ED researchers, medical educators, and practicing health professionals. I was not a first-round reviewer but appreciate your edits in response to Reviewer 1. My recommendation is for a minor revision, primarily focused on consistency and clarity in how descriptions of the studies are framed throughout the paper, as it is difficult to track within the scope of how many total studies were included, once in the thick of the paper. “Among the 32 studies that included PHC professionals...” - Needs better contextualization within the total number of studies included, rather than the number of studies that included professionals within the total number of studies.

General language example: Of the X total of studies that met inclusion criteria, Y mentioned ____. Of these, ___ percent or ___ number had these traits.

Response: We thank the reviewer for this important observation. The manuscript was revised throughout to improve consistency, clarity, and readability in the presentation of study characteristics and findings. The Results section was standardized to better contextualize subgroup analyses, frequencies, and proportions within the total number of included studies whenever appropriate.

Examples:

L227-228: On the studies including PHC users, four revealed that the majority of users were married [49,50,52,54].”

L231-232: Of the studies included, 32 involved PHC professionals. Among these, 16 studies predominantly involved physicians, while other health professional categories were underrepresented [58,63,69,72,74–81,83–86].

L234-237: Regarding study settings, most studies (n=45 were conducted exclusively in PHC, while nine studies in environments generalizable to PHC, such as university-affiliated services and hospital-affiliated services, and two combined both contexts. In terms of location, 31 studies were conducted in urban and metropolitan areas (Table S1).

Comment 2: Table 4 - Main barriers and facilitators to screening reported by professionals – Can enhance clarity by nesting frequency within total number of relevant articles.

Response: As suggested, Table 4 was revised to improve clarity by contextualizing the frequency of barriers and facilitators within the total number of relevant studies. Additionally, the table was reorganized according to the prevalence of studies reporting each barrier and facilitator.

Comment 3: Line 228: “Four studies revealed that the majority of users were married...”

Response: As suggested, the sentence was revised to improve clarity and readability regarding the sociodemographic characteristics reported across studies.

L227-229: (…) On the studies including PHC users, four revealed that the majority of users were married [49,50,52,54].

Comment 4: Line 98 “What are the characteristics of the validated strategies for at screening and their application...” - The language “for at” is unclear and potentially a typo.

Response: Thank you for identifying this issue. The typographical error was corrected in the revised manuscript.

L96-97: What are the characteristics of the validated strategies for ED screening and their application…

Comment 5: Make sure all “eating disorders” are abbreviated to ED (e.g., line 393).

Response: As suggested, the manuscript was revised to ensure consistency in the use of the abbreviation “ED” throughout the text.

Comment 6: Line 327 - “Female professionals showed a greater tendency to adopt the screening questionnaire...” Can add existing citations that this is consistent with literature showing that males are less likely to screen.

Response: As suggested, the sentence in the Results section was revised for greater clarity.

L327-329: In one study, female professionals showed a greater tendency to adopt the screening questionnaire to the detriment of the evaluation of clinical signs [80].

Additionally, literature was incorporated into the Discussion to contextualize this finding. A study on substance use screening during pregnancy was included, showing that female obstetricians/gynecologists were more likely to believe in the effectiveness of screening and to adopt standardized screening tools in clinical practice [111].

L435-442: Gender differences in attitudes toward the use of screening questionnaires were observed in the study by Boulé McSherry (2002). The findings suggest that female professionals were more likely than their male counterparts to adopt screening strategies based on standardized instruments [80]. However, the factors underlying these differences remain unclear. Similar findings have been reported in other areas of mental health-related screening. A study on substance use screening during pregnancy found that female obstetricians/gynecologists were more likely to believe in the effectiveness of screening and to adopt standardized screening tools in clinical practice [111].

Comment 7: While I can infer as a reviewer, please provide a rationale for excluding ARFID.

Response: Thank you for this important observation. A clarification regarding the exclusion of ARFID was added to the manuscript. ARFID was excluded from this review because the preliminary search did not identify studies evaluating validated screening strategies for ARFID in adolescents and adults within the scope of primary health care. As the objective of this review was to map existing screening strategies applicable to primary health care, only eating disorders for which screening approaches had been identified in the literature were included.

Comment 8: Lines 401 to 408 can use another review and tighter editing.

Comment 9: Line 401: “Even so, these tools have limitations in terms of diagnostic accuracy...” - I recommend rephrasing to “being subject to both false positives and missed capture” if keeping.

Response: As suggested, the paragraph was revised to acknowledge both false positives and missed cases, improving conceptual accuracy and balance in the discussion.

L400-402: Even so, these tools have limitations in terms of diagnostic accuracy, being subject to false positives and missed cases [21]. While false positives may lead to unnecessary referrals, false negatives may delay identification and timely access to care.

Comment 10: “In addition, screening can influence the trajectory of care...” - This feels dangerous to include and not robustly supported. Literature points to the importance of early intervention within first three years of illness onset as instrumental to positive clinical outcomes.

Response: Thank you for this valuable observation. We agree that the statement regarding the influence of screening on the trajectory of care was not sufficiently supported by the available evidence and could be interpreted beyond the scope of the findings discussed in this review. Therefore, we have removed this statement from the manuscript

Comment 11: The above two statements and the section in general needs to be conceptually merged with this stronger point regarding cultural biases in screening instruments.

Response: As suggested, the section was revised to improve conceptual integration between the limitations of screening instruments and cultural biases that may affect the identification of underrepresented populations.

L402-406: Additionally, another critical issue concerns the cultural biases present in screening instruments, which tend to privilege cultural stereotypes of gender, race, and social class, for example, the profile of white women from the middle and upper class background while a neglecting populations experiencing greater social vulnerability, such as individuals in situations of food insecurity [106].

Additional note: We identified an inconsistency in the reference list and corrected it in the revised manuscript. Reference 110 was inadvertently omitted, and the reference previously numbered as 110 corresponded to reference 111. In addition, a new reference (reference 112) was included in response to the reviewers’ suggestion to incorporate additional relevant studies in the Discussion section. The numbering has been updated accordingly throughout the manuscript.

We sincerely thank the reviewer and the editors for their valuable feedback. We believe that these revisions have substantially strengthened the manuscript and hope that it is now suitable for publication.

Sincerely,

Lisane da Silva Oliveira

Attachment Submitted filename: Response_to_Reviwes_auresp_2.docx

# Peer review: Decision Letter 2

**DOI:** 10.1371/journal.pone.0347184.r005

**Authors:** Zehra Batu

2 Jul 2026

Validated strategies for screening for eating disorders in primary health care: A scoping review with a focus on adolescents and adults

PONE-D-25-28582R2

Dear Dr. Lisane da Silva Oliveira;

We’re pleased to inform you that your manuscript has been judged scientifically suitable for publication and will be formally accepted for publication once it meets all outstanding technical requirements.

Within one week, you’ll receive an e-mail detailing the required amendments. When these have been addressed, you’ll receive a formal acceptance letter and your manuscript will be scheduled for publication.

An invoice will be generated when your article is formally accepted. Please note, if your institution has a publishing partnership with PLOS and your article meets the relevant criteria, all or part of your publication costs will be covered. Please make sure your user information is up-to-date by logging into Editorial Manager at Editorial Manager® and clicking the ‘Update My Information' link at the top of the page. For questions related to billing, please contact billing support.

If your institution or institutions have a press office, please notify them about your upcoming paper to help maximize its impact. If they’ll be preparing press materials, please inform our press team as soon as possible -- no later than 48 hours after receiving the formal acceptance. Your manuscript will remain under strict press embargo until 2 pm Eastern Time on the date of publication. For more information, please contact onepress@plos.org.

Kind regards,

Zehra Batu

Academic Editor

PLOS One

Additional Editor Comments (optional):

Reviewers' comments:

Reviewer's Responses to Questions

**Comments to the Author**

1. If the authors have adequately addressed your comments raised in a previous round of review and you feel that this manuscript is now acceptable for publication, you may indicate that here to bypass the “Comments to the Author” section, enter your conflict of interest statement in the “Confidential to Editor” section, and submit your "Accept" recommendation.

Reviewer #1: All comments have been addressed

Reviewer #2: All comments have been addressed

**********

2. Is the manuscript technically sound, and do the data support the conclusions?

The manuscript must describe a technically sound piece of scientific research with data that supports the conclusions. Experiments must have been conducted rigorously, with appropriate controls, replication, and sample sizes. The conclusions must be drawn appropriately based on the data presented.

Reviewer #1: Yes

Reviewer #2: Yes

**********

3. Has the statistical analysis been performed appropriately and rigorously?

Reviewer #1: N/A

Reviewer #2: N/A

**********

4. Have the authors made all data underlying the findings in their manuscript fully available?

The PLOS Data policy requires authors to make all data underlying the findings described in their manuscript fully available without restriction, with rare exception (please refer to the Data Availability Statement in the manuscript PDF file). The data should be provided as part of the manuscript or its supporting information, or deposited to a public repository. For example, in addition to summary statistics, the data points behind means, medians and variance measures should be available. If there are restrictions on publicly sharing data—e.g. participant privacy or use of data from a third party—those must be specified.

Reviewer #1: Yes

Reviewer #2: Yes

**********

5. Is the manuscript presented in an intelligible fashion and written in standard English?

PLOS ONE does not copyedit accepted manuscripts, so the language in submitted articles must be clear, correct, and unambiguous. Any typographical or grammatical errors should be corrected at revision, so please note any specific errors here.

Reviewer #1: Yes

Reviewer #2: Yes

**********

6. Review Comments to the Author

Please use the space provided to explain your answers to the questions above. You may also include additional comments for the author, including concerns about dual publication, research ethics, or publication ethics. (Please upload your review as an attachment if it exceeds 20,000 characters)

Reviewer #1: I thank the authors for addressing all my comments. I have no further comments.

Congratulations.

Reviewer #2: Thank you for comprehensively addressing each of my comments. This paper is great! I recommend it for publication.

**********

7. PLOS authors have the option to publish the peer review history of their article (what does this mean?). If published, this will include your full peer review and any attached files.

If you choose “no”, your identity will remain anonymous but your review may still be made public.

**Do you want your identity to be public for this peer review?**  For information about this choice, including consent withdrawal, please see our Privacy Policy.

Reviewer #1: **Yes:** Sophie PILLERON

Reviewer #2: **Yes:** Scout Silverstein

**********
